# Ghrelin improves small intestinal barrier damage in sepsis by promoting miR-143/ATG2B-mediated autophagy

**DOI:** 10.1371/journal.pone.0329488

**Published:** 2025-08-07

**Authors:** Jingquan Liu, Kai Shi, Hanhui Cai, Zihao Zheng, Bin Fan, Xianghong Yang, Ziqiang Shao

**Affiliations:** 1 Emergency and Critical Care Center, Intensive Care Unit, Zhejiang Provincial People’s Hospital (Affiliated People’s Hospital), Hangzhou Medical College, Hangzhou, Zhejiang, China; 2 Department of Respiratory Medicine, The Affiliated Hospital of Hangzhou Normal University, Hangzhou, Zhejiang, China; Tabriz University of Medical Sciences, IRAN, ISLAMIC REPUBLIC OF

## Abstract

Intestinal barrier damage is crucial for the development of sepsis. Ghrelin (GHS) can restore intestinal barrier function. However, the mechanisms of GHS on intestinal barrier damage in sepsis remain unclear. We aimed to explore the mechanisms of GHS against intestinal barrier damage in sepsis. Septic models were established by cecal ligation and puncture surgery for rats and lipopolysaccharides exposure for IEC-6 cells. Furthermore, these septic models were overexpressed miR-143 and treated with GHS. *In vivo*, small intestinal pathological injury and D-lactic acid level were detected. Tight junction protein (Claudin-1, Occludin and ZO-1) expressions and autophagosome number were evaluated. *In vitro*, cell viability, autolysosome number, and relationship between miR-143 and ATG2B were determined. miR-143, ATG2B and autophagy-related protein (Beclin-1, p62 and LC3I/LC3II) levels were evaluated in rats and cells. GHS mitigated small intestinal pathological injury and decreased D-lactic acid level for septic rats. Additionally, GHS elevated tight junction protein expressions, ATG2B, Beclin-1 and LC3I/LC3II levels, and autophagosome number, but reduced miR-143 and p62 levels for septic rats. However, miR-143 overexpression presented the opposite results. Consistently, cellular experiments found that GHS increased cell viability, autolysosome number, and presented similar results for miR-143, ATG2B and autophagy-related protein levels for lipopolysaccharides-exposed cells. Additionally, ATG2B directly targeted miR-143 in IEC-6 cells. Both animal and cellular experiments found the effects of GHS on sepsis-induced small intestinal barrier damage were reversed by miR-143 overexpression. GHS may improve small intestinal barrier damage in sepsis through miR-143/ATG2B-mediated autophagy, indicating miR-143/ATG2B was an underlying therapeutic target for sepsis.

## Introduction

Sepsis is a complicated condition resulting from the host’s abnormal response to infection [1]. Despite the extensive research has been performed on potential treatments for sepsis, the mortality rate of sepsis remains high. Previous investigations have suggested the critical role of intestinal barrier damage in sepsis [2]. From a biological perspective, the intestinal barrier blocks intestinal microbes and their metabolites into the bloodstream [3]. During sepsis, hyperinflammation injures the intestinal epithelial cells, resulting in a breakdown of the intestinal barrier and facilitating the spread of microbes and toxins. These harmful substances then stimulate the host’s immune defense mechanisms, resulting in severe clinical symptoms [4]. The intestine has always been considered the ‘engine’ of sepsis. Thus, it is urgent to fund efficient therapeutic approaches for intestinal barrier damage in sepsis.

Autophagy, a common phenomenon that manages intracellular metabolism, has been reported to involve in maintaining intestinal barrier. Activating autophagy reduces paracellular permeability of the intestinal epithelium [5]. Conversely, blocking autophagy leads to the dysfunctions of tight junction proteins and an increase in barrier permeability [6]. Melatonin has been reported to attenuate sepsis-caused small intestinal barrier dysfunction by upregulating Sirt3-mediated autophagy [7]. As a target gene of miR-143, ATG2B is directly implicated in autophagy by participating in the formation of autophagosomes [8]. In addition, miR-143 is upregulated in septic patients, and upregulating ATG2B in exosomes derived from lipopolysaccharide (LPS)-exposed bone marrow mesenchymal stem cells can mitigate septic liver damage [9,10]. Thus, miR-143/ATG2B-mediated autophagy plays a key role in intestinal barrier damage in sepsis.

Ghrelin (GHS), a kind of hormone predominantly secreted by gastric mucosal cells, has been proven to have anti-inflammatory activity, immunomodulatory property, as well as the capacity to restore intestinal barrier function [11,12,13]. GHS has also been reported to play a protective role in human and experimental sepsis [11]. Although the regulation of GHS on autophagy has been proved by both animal and cellular experiments, it remains uncertain whether GHS improves small intestinal barrier damage in sepsis by promoting miR-143/ATG2B-mediated autophagy [14,15].

Thus, this study established septic models *in vivo* and *in vitro* by cecal ligation and puncture (CLP) surgery and LPS exposure, respectively. Additionally, miR-143 was overexpressed both *in vivo* and *in vitro* to further confirm the mechanisms of GHS in treating sepsis. The aim of the study was to offer an experimental basis for the potential therapeutic target of GHS in sepsis, thereby facilitating the clinical application of GHS in sepsis.

## Materials and methods

### Animals and ethical approval

Male SD rats (6–8-weeks-old) were from Shanghai SLAC Laboratory Animal Co., Ltd. The animal experiments conducted in this study received approval from the Laboratory animal management and ethics committee of Hangzhou Hunter Testing Biotechnology Co., Ltd. (Ethical approval No. IACUC/HTYJ-8201–94), and the animals were cared by the guidelines of the Institutional Animal Care and Use Committee. The rats were maintained in standard conditions (free access to food and water, temperature 22 ± 2°C, and 12 h/12 h light/dark cycles).

### Grouping and regulating miR-143 expression

The rats were allocated into the following groups randomly: sham group, CLP group, CLP + GSH group, CLP + LV-NC group, CLP + LV-miR-143 group, CLP + LV-NC + GHS group and CLP + LV-miR-143 + GHS group (n = 5).

The lentivirus vector overexpressing miR-143 (GV309, LV-miR-143) and the blank lentivirus vector (LV-NC) were sourced from GeneChem. To modulate miR-143 level, the rats were injected with LV-miR-143, LV-NC, or phosphate buffered saline every other day according to the grouping. Each rat underwent 5 injections, with the first 2 administered via intraperitoneal route, and the remaining 3 were injected intravenously through caudal vein. Each injection involved a virus dose of 1 × 10^7^ ifu [16].

### Establishment of septic rats models and GSH treatment

After finishing the last lentiviral injection, the rat septic models were established using the CLP surgery as described previously with some modifications [17]. Prior to the surgery, the rats were deprived of food for 12 h, but had unrestricted access to water. Then, anesthesia was achieved by injecting 1% sodium pentobarbital (50 mg/kg) intraperitoneally. Following general anesthesia, the cecum of the rats was exposed by laparotomy. Then, the exposed cecum was carefully ligated using a 3-gauge thread at about 3/4 of the cecal end. Then, an 18 G trocar was applied to perforate two holes in the cecum, and rice-sized feces were excreted by squeezing the punctured cecum. Following that, the cecum was returned to abdominal cavity, and the incision was closed in layers. The cecum of the rats in the sham group was exposed without requiring ligation and perforation. All surgery was performed under sodium pentobarbital anesthesia, and all animal experiments were carried out with utmost care to minimize the suffering of rats.

Following CLP surgery, the rats in the CLP + GSH, CLP + LV-NC + GHS and CLP + LV-miR-143 + GHS groups were injected with 2 nmol GHS via tail vein. Later, these rats were continuously infused with GHS via a micropump (8 mL/h) for 24 h (100 mM GSH in saline) [18]. The rats in the other groups were received with the same amount of saline using the same methods. A weight loss exceeding 20% was used as a humane endpoint. The animals’ health and behavior were monitored daily. No animals reached a humane endpoint and no animals died unexpectedly in this study. Upon reaching the humane endpoint or completing this experiment, the rats were euthanized using overdose of CO_2_. At the end of the study, blood samples of the rats were obtained through cardiac puncture, and the small intestine tissues were collected for further study.

### Hematoxylin-eosin (H&E) staining

The small intestine tissues were fixed overnight in paraformaldehyde. Subsequently, the fixed specimens were dehydrated, transparent, and followed by embedding in paraffin. Later, the samples were sliced and stained with hematoxylin (H3136, Sigma) and eosin (E4009, Sigma) to view the pathological changes in small intestine tissues using an optical microscope (Eclipse Ci-L, Nikon).

### Measurement of D-lactic acid (D-LA) level

The collected blood samples were centrifuged to obtain the serum. Then, the level of D-LA in serum was assessed using D-LA content assay kits (BC5355, Solarbio), strictly following the provided instructions.

### Transmission electron microscopy (TEM)

In short, the small intestine tissues were fixed using 2.5% glutaraldehyde and 1% osmium tetroxide in turn. Then, the sections underwent dehydration with ethanol, immersion with acetone and embedding in epoxy resin. After that, the tissues were sliced and stained by 4% uranyl acetate and 2.5% lead nitrate. Finally, the autophagosomes were viewed and counted with a transmission electron microscope (H7650, Hitachi).

### Cell culture and treatment

IEC-6 rat small intestinal epithelial cells (iCell-r016) were obtained from iCell Biotechnology Co., Ltd. A total of 1 × 10^9^ cells were inoculated into 6-well plates at a density of 4 × 10^5^ cells per well and cultured in Dulbecco’s modified eagle medium containing 10% fetal bovine serum and 1% penicillin/streptomycin. IEC-6 cells were digested and passaged using pancreatin, with those in the logarithmic phase being collected for further experiments.

Next, IEC-6 cells were separated into control, LPS, LPS + GHS, LPS + GHS + miR-NC and LPS + GHS + miR-143 groups. To regulate miR-143 levels, IEC-6 cells in the LPS + GHS + miR-143 group were transfected with miR-143 overexpression lentivirus, while those in the LPS + GHS + miR-NC group were transfected with vectors containing unrelated sequences. Then, 1 μM/L GHS was added to the cells in the GHS treatment groups for 96 h [19]. After incubation with GHS for 48 h, the cells in all groups, except for the control group, were exposed to 5 μg/mL LPS for 48 h to mimic sepsis [20,21]. The cells in the control group did not receive any treatments. Successful overexpression of miR-143 was verified by quantitative real time polymerase chain reaction (qPCR).

### qPCR

Total RNA of the small intestine tissues and IEC-6 cells was isolated by EZ-10 total RNA extraction kits (B618583-0250, BBI), then, the cDNA was synthesized using TRUEscript RT MasterMix (OneStep gDNA Removal, PC7002, Aidlab Biotechnologies Co., Ltd.). Then, the expressions of miR-143 and ATG2B mRNA in the samples were quantified through qPCR with SYBR Green qPCR Mix. The relative expressions of targeted mRNA were determined using the primers utilized in this experiment are listed in Table 1. The qPCR cycling program was as follows: denaturation at 95°C for 10 min and 40 cycles of 95°C for 15 s, and 60°C for 60 s.

**Table 1:** Primer sequence of the genes for qPCR analysis.

Gene	Forward Primer	Reverse Primer
Rat miR-143-3p	GCCGAGTGAGATGAAGCAC	CTCAACTGGTGTCGTGGA
Rat U6	CTCGCTTCGGCAGCACA	AACGCTTCACGAATTTGCGT
Rat ATG2B	ATCAAGAAGAGGGCCTGTCG	TGCCACCTAGCACGTTTGAT
Rat β-actin	GTCACCCACACTGTGCCCATCT	ACAGAGTACTTGCGCTCAGGAG

### Cell viability assay

After finishing transfection and treating for GHS for 48 h, IEC-6 cells were seeded in 96-well plates. Then, the cells were treated with LPS for 48 h, after that, 7 μL cell titer-blue reagent (G8080, Promega) was added to every well followed by incubation for 1 h. The fluorescence signal of the plates was determined at emission and excitation wavelengths of 590 nm and 560 nm, respectively.

### Western blot analysis

Small intestine tissues and IEC-6 cells were lysed by radioimmunoprecipitation assay buffer to extract the total proteins. After that, the bicinchoninic acid assay was utilized to determine the protein concentration. Then, the proteins were separated by 10% sodium dodecyl sulfate polyacrylamide gel electrophoresis, blotted into polyvinylidene difluoride membranes, and blocked in 5% skimmed milk. After that, the membranes were soaked with appropriate primary antibodies overnight at 4°C. Following washing, the secondary antibodies were added to the membranes. The electrochemiluminescence kits were applied to visualize the proteins, and ImageJ software was applied to quantify the gray value of the bands. The details of the primary antibodies are presented in Table 2.

**Table 2:** Primary antibody information for western blot analysis.

Antibody	Source	Cat No.	Dilutions
ATG2B Antibody	ABclonal	A8498	1:1000
Beclin-1 Antibody	Affinity	AF5128	1:1000
p62 Antibody	Affinity	AF5384	1:1000
LC3 Antibody	CST	4108s	1:1000
claudin-1 Antibody	Affinity	AF0127	1:1000
occludin Antibody	abcam	AB216327	1:1000
ZO-1 Antibody	Affinity	AF5145	1:1000
Anti-rabbit IgG, HRP-linked Antibody	CST	7074	1:6000
β-actin Antibody	Proteintech	81115-1-RR	1:10000

### Monitoring autophagic flux

To monitor autophagic flux, IEC-6 cells were transfected with AdPlus-mCherry-GFP-LC3B adenovirus (C3012-10 ml, Biyotime) for 48 h. Thereafter, IEC-6 cells were treated according to the specified grouping. After that, IEC-6 cells were cultured in fresh media and the fluorescence was observed under confocal microscopy (LSM880, Zeiss). Given that GFP is acid-sensitive, and its fluorescent activity will diminish in an acidic microenvironment of the autolysosome. Thus, autophagosomes convert to autolysosomes will appear as red puncta.

### Dual-luciferase reporter assay

The wild type (WT) and mutant (MUT) sequences of ATG2B were inserted into pmirGLO vectors. Then, ATG2B WT or ATG2B MUT and miR-143 mimic or corresponding control were cotransfected into IEC-6 cells with lipofectamine 3000. After transfection for 48 h, IEC-6 cells were collected and lysed, and the Firefly/Renilla dual-luciferase activity was detected with a dual-luciferase reporter assay system, with Renilla luciferase serving as a control.

### Statistical analysis

The results were analyzed with SPSS 20.0, and the values were displayed as mean±standard deviation. Comparisons between two groups were conducted by t test. For comparisons involving multiple groups, one-way analysis of variance followed by Tukey tests was performed. Furthermore, Kruskal-Wallis H test was utilized when the data didn’t meet the criteria for normal distribution, and Dunnett T3 test was employed for data with unequal variances. P < 0.05 was deemed statistically significant.

## Results

### GSH alleviated barrier damage, regulated miR-143/ATG2B expression and enhanced autophagy in small intestinal tissues for septic rats

**Fig 1:**
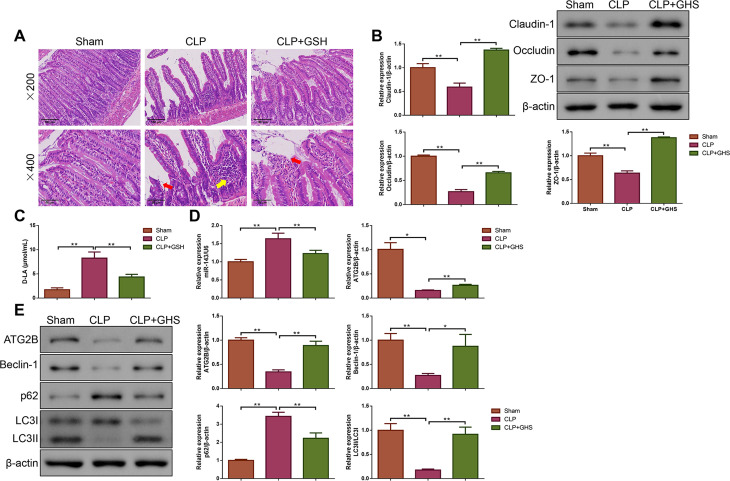
Ghrelin (GHS) improved barrier damage, modulated miR-143/ATG2B expression, and promoted autophagy in the small intestine for rats with sepsis. **(A)** The pathological injury in the small intestine were evaluated by hematoxylin-eosin (H&E) staining. Magnification×200, scale bar = 100 μm; Magnification×400, scale bar = 500 μm. Red arrows: the intestinal mucosa exhibited partial shedding; Yellow arrows: the inflammatory cell infiltration. **(B)** The expressions of the main tight junction proteins (including Claudin-1, Occludin and ZO-1) in the small intestine were detected by Western blot analysis. n = 3. **(C)** The serum D-lactic acid (D-LA) level was detected in each group. n = 5. **(D)** Quantitative real time polymerase chain reaction (qPCR) was used to test the expressions of miR-143 and ATG2B mRNA in the small intestine. n = 3. **(E)** The expressions of ATG2B and autophagy-related protein (Beclin-1, p62 and LC3I/LC3II) in the small intestine were quantified by Western blot analysis. n = 3. *P < 0.05; **P < 0.01. Results were presented as mean±standard deviation (SD).

First, the pathological changes of the small intestinal tissues revealed that in the sham group, the overall structure of the tissues was basically normal, with cells arranged closely without apparent injury. However, the small intestinal tissues of the CLP rats exhibited severe damage, with loosely arranged and severely shortened villi, sloughed epithelial cells, and notable inflammatory cell infiltration. As expected, GHS effectively mitigated the damage to the small intestinal tissues (Fig 1A). It can be observed from Figs 1B–1C that Claudin-1, Occludin and ZO-1 protein expressions were decreased, while D-LA level was increased in the rats underwent CLP surgery (P < 0.01). However, GSH intervention reversed those situations (P < 0.01). In addition, Fig 1D found the elevated miR-143 level and decreased ATG2B mRNA level caused by CLP surgery were restored after GHS treatment (P < 0.01). As exhibited in Fig 1E, CLP surgery led to the decreases of ATG2B, Beclin-1 and LC3I/LC3II protein levels, along with an increase in p62 protein level (P < 0.01). In contrast, GHS treatment enhanced ATG2B, Beclin-1 and LC3I/LC3II protein levels, but reduced p62 protein level for CLP rats (P < 0.05 or P < 0.01).

### miR-143 overexpression exacerbated barrier damage and decreased ATG2B level in small intestinal tissues for septic rats treated with GHS

**Fig 2:**
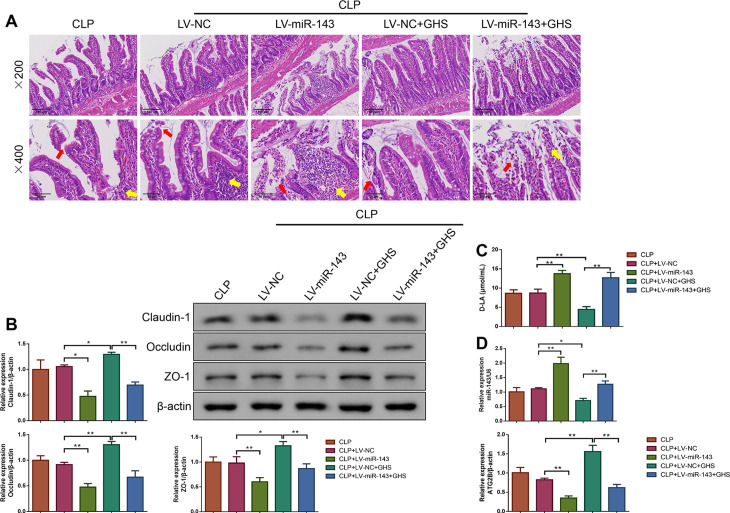
miR-143 overexpression worsened barrier damage and reduced ATG2B level in the small intestine for septic rats treated with GHS. **(A)** Small intestinal pathological injury of each group were measured by H&E staining. Magnification×200, scale bar = 100 μm; Magnification×400, scale bar = 500 μm. Red arrows: the intestinal mucosa exhibited partial shedding; yellow arrows: the inflammatory cell infiltration. **(B)** Claudin-1, Occludin and ZO-1 protein levels in small intestine tissues of each group were detected by Western blot analysis. n = 3. **(C)** The D-LA level in the serum of each group was detected. n = 5. **(D)** The levels of miR-143 and ATG2B mRNA in small intestine tissues in each group were quantified by qPCR. n = 3. *P < 0.05; **P < 0.01. Results were presented as mean±SD.

As exhibited in Fig 2A, the pathological changes of the small intestinal tissues in the CLP + LV-NC group were similar to those observed in the CLP group. Relative to the CLP + LV-NC group, miR-143 overexpression exacerbated the small intestinal injury, while GHS treatment led to an improvement. Interestingly, miR-143 overexpression worsened the small intestinal injury in the CLP + LV-NC + GHS group. Additionally, compared to the CLP + LV-NC group, the Claudin-1, Occludin and ZO-1 protein expressions were decreased, while D-LA level was increased in the CLP + LV-miR-143 group (P < 0.05 or P < 0.01), contrasting with the trends observed in the CLP + LV-NC + GHS group (P < 0.05 or P < 0.01). As expected, miR-143 overexpression decreased Claudin-1, Occludin and ZO-1 protein expressions and increased D-LA levels for the CLP + LV-NC + GHS group (Figs 2B–2C, P < 0.01). It was also noted that relative to the CLP + LV-NC group, the CLP + LV-miR-143 group had a higher miR-143 level and a lower ATG2B level (P < 0.01), while the CLP + LV-NC + GHS group exhibited an opposite trend (P < 0.05 or P < 0.01). However, miR-143 overexpression increased miR-143 level but decreased ATG2B level for the CLP + LV-NC + GHS group (Fig 2D, P < 0.01).

### miR-143 overexpression inhibited autophagy in small intestinal tissues for septic rats treated with GHS

**Fig 3:**
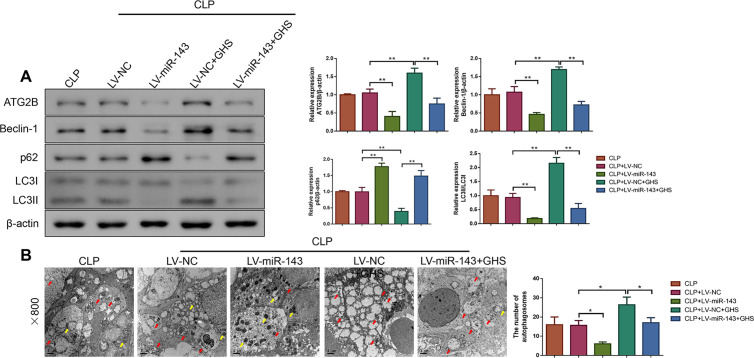
miR-143 overexpression suppressed autophagy in the small intestine for sepsis rats treated with GHS. **(A)** Western blot analysis of ATG2B and autophagy-related protein (Beclin-1, p62 and LC3I/LC3II) expressions in the small intestine of the rats. n = 3. **(B)** Transmission electron microscopy was applied to test the number of autophagosomes in the small intestine of the rats. Magnification×800, scale bar = 1 μm. n = 3. *P < 0.05; **P < 0.01. Results were presented as mean±SD.

Next, we explored the impact of miR-143 overexpression on autophagy for septic rats that received GHS treatment. It was observed from Fig 3A that relative to the CLP + LV-NC group, ATG2B, Beclin-1, and LC3I/LC3II protein levels were reduced, but p62 protein level was elevated in CLP + LV-miR-143 rats (P < 0.01); in contrast, CLP + LV-NC + GHS rats exhibited the opposite effects (P < 0.01). Of note, the increased ATG2B, Beclin-1, and LC3I/LC3II protein levels and decreased p62 level observed in CLP + LV-NC + GHS rats were counteracted by miR-143 overexpression (P < 0.01). Similarly, miR-143 overexpression led to a reduction in the number of autophagosomes (P < 0.05), while GHS treatment resulted in an increase in the number of autophagosomes in the small intestinal tissues for CLP + LV-NC rats (P < 0.05). However, the rise in autophagosome number induced by GHS treatment in the CLP + LV-NC + GHS group was reversed by miR-143 overexpression (Fig 3B, P < 0.05).

### GSH alleviated sepsis in vitro by enhancing autophagy via targeting miR-143/ATG2B

**Fig 4:**
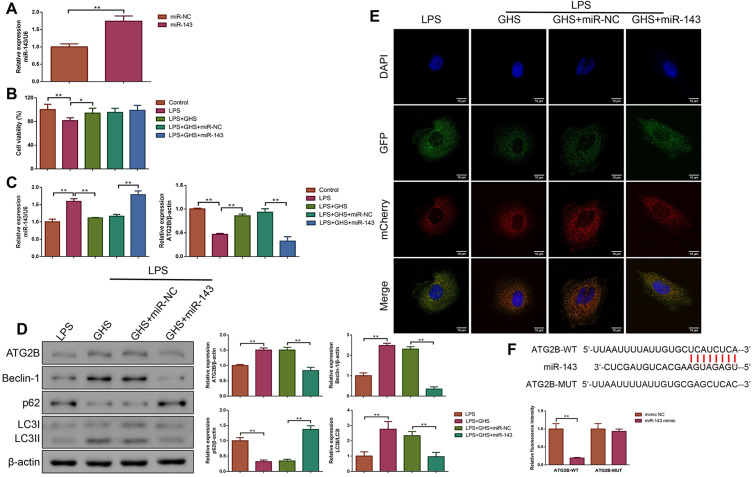
GSH improved cellular models of sepsis by promoting autophagy through the targeting of miR-143/ATG2B. **(A)** qPCR was conducted to verify miR-143 overexpression. n = 3. **(B)** The cell viability of each group was detected by cell titer-blue assay. n = 3. **(C)** The expression levels of miR-143 and ATG2B in each group were assessed by qPCR. n = 3. **(D)** The expressions of ATG2B and autophagy-related proteins (including Beclin-1, p62 and LC3I/LC3II) in the cells were tested by Western blot analysis. n = 3. **(E)** The number of autophagosomes in each group was detected using AdPlus-mCherry-GFP-LC3B adenovirus. Magnification×630, scale bar = 10 μm. n = 3. **(F)** The target relationship between miR-143 and ATG2B in IEC-6 cells was determined by dual-luciferase reporter assay. n = 3. *P < 0.05; **P < 0.01. Results were presented as mean±SD.

To further explore the roles and mechanisms of GHS in sepsis, IEC-6 cells were transfected with miR-143 overexpression lentivirus, treated with GHS and stimulated with LPS. The transfection efficiency of the miR-143 overexpression lentivirus was confirmed by qPCR, as exhibited in Fig 4A, there was a notable increase in miR-143 expression in the miR-143 group (P < 0.01). Subsequently, the cell viability of each group was detected. As exhibited in Fig 4B, there was a reduction in cell viability in the LPS group (P < 0.01). However, following GHS treatment, the viability of IEC-6 cells stimulated by LPS was elevated (P < 0.05). Moreover, LPS stimulation induced an increase in miR-143 expression, but a decrease in ATG2B mRNA for IEC-6 cells (P < 0.01). However, GHS treatment reversed these situations (P < 0.01), but miR-143 overexpression further counteracted the effects of GHS on LPS-stimulated IEC-6 cells (Fig 4C, P < 0.01). By Western blot analysis, we found that ATG2B, Beclin-1, and LC3I/LC3II protein expressions were elevated in the LPS + GHS group, while p62 protein level was reduced (P < 0.01). Nevertheless, the expressions of these proteins were reversed upon miR-143 overexpression (Fig 4D, P < 0.01). The mechanisms of GSH on the autophagy of sepsis were further confirmed with Ad-mCherry-GFP-LC3B, the results presented in Fig 4E clearly revealed that after treatment with GHS, an increase in red puncta was noted in LPS-stimulated cells, which indicated the increased autolysosomes. As expected, this effect was counteracted by miR-143 overexpression. Moreover, Fig 4F illustrated that miR-143 mimic specifically inhibited the luciferase activity of the ATG2B-WT group (P < 0.01).

## Discussion

Sepsis represents the leading cause of mortality in intensive care units, and preventing and treating sepsis are critical topics for both basic and clinical research, while intestinal barrier damage is the initial phase of the systemic inflammation triggered by sepsis [22]. Thus, identifying an effective method to prevent and improve intestinal barrier damage in sepsis is particularly crucial. This study found that GHS could improve small intestinal barrier damage in sepsis by promoting miR-143/ATG2B-mediated autophagy.

Since its first report in 1999, GHS has attracted increasing attention for its involvement in various biological processes [23]. GHS has been identified as a promising therapeutic option for treating intracerebral hemorrhage-caused intestinal barrier disruption [24]. In addition, GHS has been reported to prevent intestinal barrier dysfunction in dextran sulphate sodium-caused colitis [25]. It is also noteworthy that GHS has the potential to be developed as a drug for treating sepsis, the anti-inflammatory properties of GHS have been well demonstrated in animal septic models [26]. Wu et al. have revealed that GHS can improve the survival rate of animals undergoing CLP surgery from day 2 [27]. A study conducted by Siegl et al. has observed that, after treatment for GHS for 6 h, serum levels of IL-6, IL-10 and IL-1β are decreased, while serum level of TNF-α levels is increased in sepsis mice; after treatment for GHS for 24 h, similar trends are observed in serum levels of IL-10, IL-1β and TNF-α; after treatment for GHS for 48 h, the similar trend can still be observed in serum level of IL-1β [28]. Moreover, the combination of GHS and growth hormone has been demonstrated to protect the lungs, livers and kidneys from damage in aged septic rats [29]. Thus, this research explored the potential effects and mechanisms of GHS on sepsis-associated small intestinal barrier damage. The findings clearly revealed that GHS not only mitigated small intestinal pathological injury and barrier damage, but also enhanced autophagy in small intestinal tissues of rats with sepsis.

Autophagy is another form of programmed cell death that differs from apoptosis. Autophagy is a highly complex process, and is characterized by autophagosome formation [30]. During autophagy, autophagy-associated proteins, such as LC3 and Beclin1 are increased, and the autophagy substrate-p62 is diminished [31]. Research conducted over the last decade has highlighted the critical role for autophagy in sepsis-related diseases and intestinal barrier function [32,33]. In addition, it has been revealed that remifentanil can attenuate intestinal injury caused by sepsis through the induction of autophagy [21]. Thus, effective control of autophagy is of particular importance in improving intestinal barrier damage in sepsis.

It is well documented that miR-143 acts as an upstream regulator of ATG2B, and ATG2B plays a crucial role in the regulation of autophagy [34]. ATG2B can encode a protein necessary for autophagy, which is implicated in the formation of autophagosomes [35]. In a series of cell experiments, Zhang et al. have proved that miR-143-3p inhibits autophagy in deep vein thrombosis by targeting ATG2B [36]. Serum miR-143 levels are upregulated in septic patients, and may serve as an effective biomarker for differentiating between systemic inflammatory response syndrome and sepsis [37]. Furthermore, upregulated ATG2B in exosomes derived from LPS-exposed bone marrow mesenchymal stem cells has been shown to mitigate septic liver damage [10]. Thus, it is speculated that modulation of miR-143/ATG2B-mediated autophagy may be an effective strategy to ameliorate intestinal barrier damage in sepsis. Our research provided evidence that ATG2B directly targeted miR-143 in IEC-6 cells. Moreover, miR-143 overexpression exacerbated barrier injury, reduced ATG2B level, and inhibited autophagy in the small intestines of septic rats, confirming the prominent roles of miR-143/ATG2B-mediated autophagy in sepsis-associated small intestinal barrier damage.

GHS is an underlying candidate to protect small intestinal epithelium from sepsis-caused damage by promoting autophagy [18]. Nevertheless, the potential mechanisms of GHS on autophagy control of sepsis-associated small intestinal barrier damage are still not completely understood. The findings of this study demonstrated that GHS lowered miR-143 level but increased ATG2B expression for septic rats. More importantly, the protective roles of GHS treatment on small intestinal pathological injury and barrier damage as well as the autophagy in small intestinal tissues of septic rats were reversed by miR-143 overexpression. Consistently, *in vitro*, GHS enhanced the viability and autophagy of LPS-stimulated IEC-6 cells, whereas miR-143 overexpression partially reversed these effects, which suggested that GHS may improve small intestinal mucosal barrier damage in sepsis by promoting miR-143/ATG2B-mediated autophagy.

Nevertheless, it should be noted that the study is not without limitations, and the primary limitation is the absence of different GHS dose treatments. This means that we are unable to investigate the dose-dependent roles of GHS on sepsis-associated small intestinal barrier damage. In the future, we will verify the key findings of this study with multiple doses of GHS, thereby providing a clearer understanding of the roles and mechanisms of GHS on sepsis-associated small intestinal barrier damage.

## Conclusion

In conclusion, this research found that GHS had an improved effect on small intestinal mucosal barrier damage in sepsis, which may be achieved by promoting miR-143/ATG2B-mediated autophagy. We believe that our findings are important in understanding the potential target of miR-143/ATG2B in sepsis-associated small intestinal barrier damage and will lead to further research in this area.

## Supporting information

S1 File Raw data of the study. (XLSX)

S2 File SPSS data of the study. (XLSX)

S3 File Raw images of the study. (PDF)
