# Supplementary material for: Ghrelin improves small intestinal barrier damage in sepsis by promoting miR-143/ATG2B-mediated autophagy
Source: PLoS One. 2025 Aug 7;20(8):e0329488. doi: 10.1371/journal.pone.0329488 (PMC12331055; doi:10.1371/journal.pone.0329488)

Fig.1A for Sham group (Magnification×200)

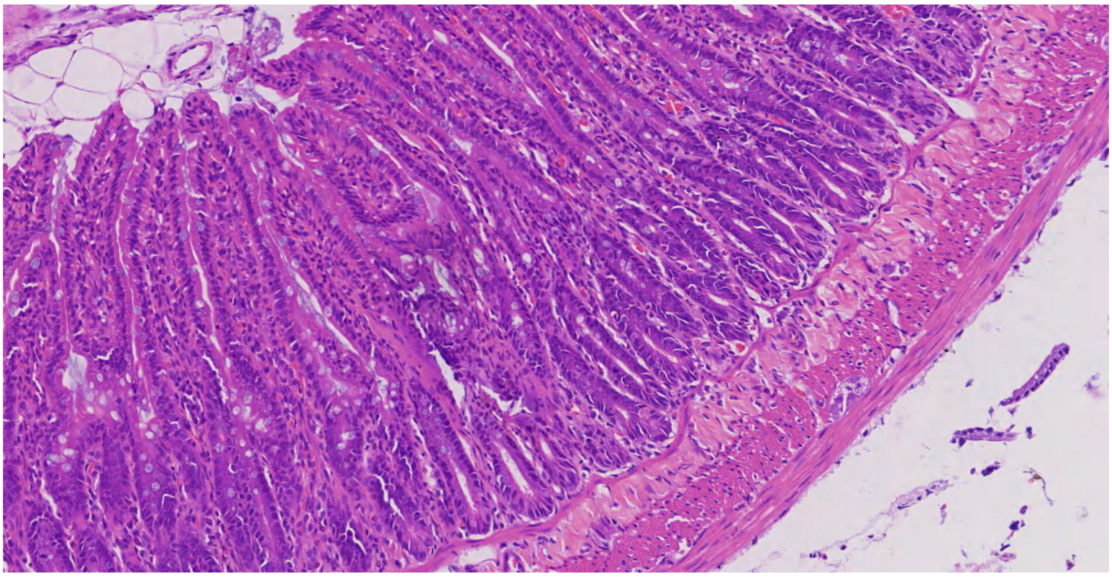

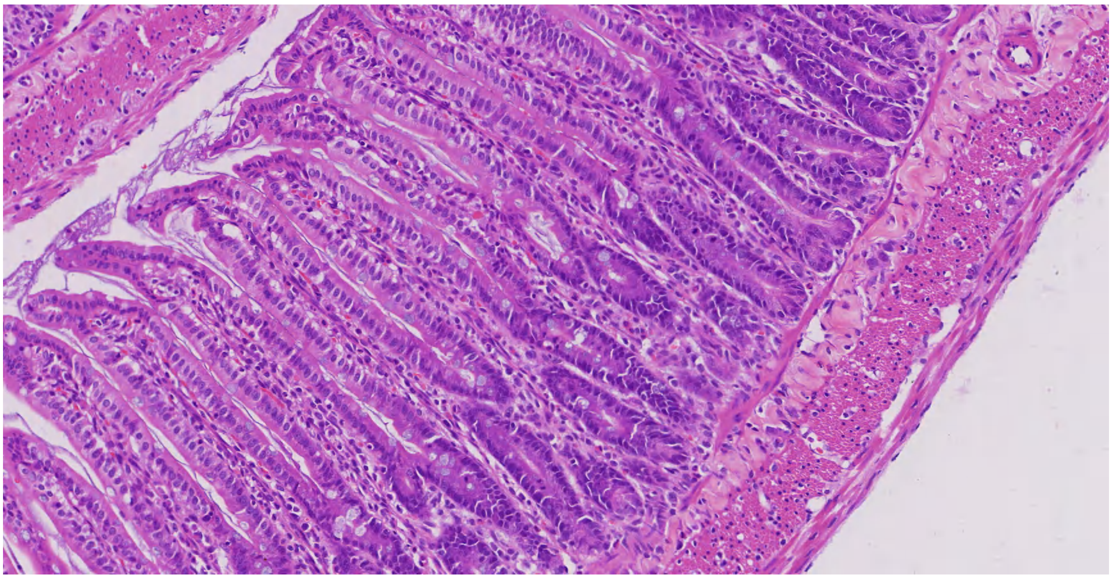

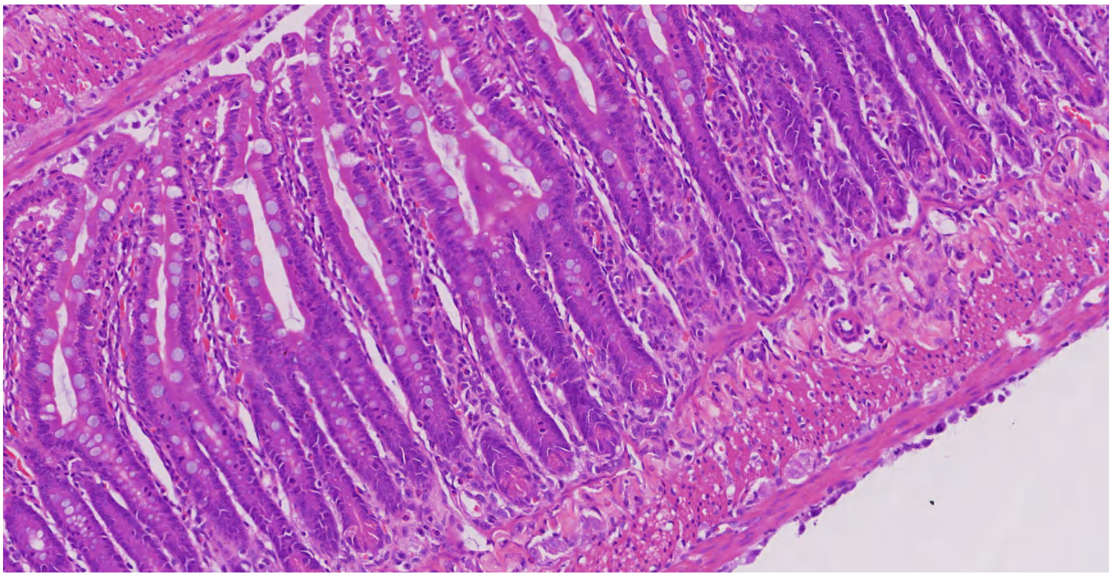

Fig.1A for Sham group (Magnification×400)

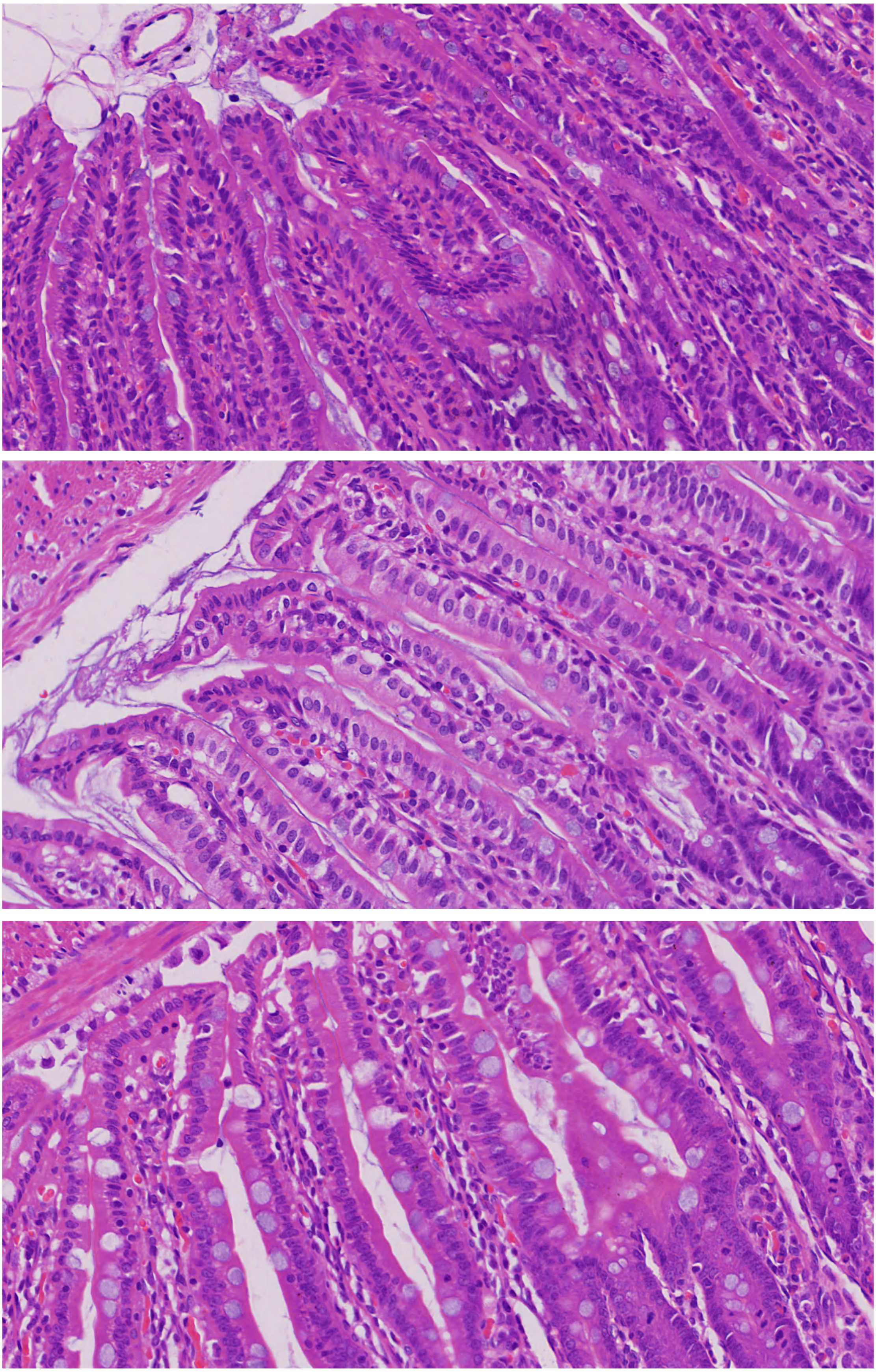

Fig.1A for CLP group (Magnification × 200)

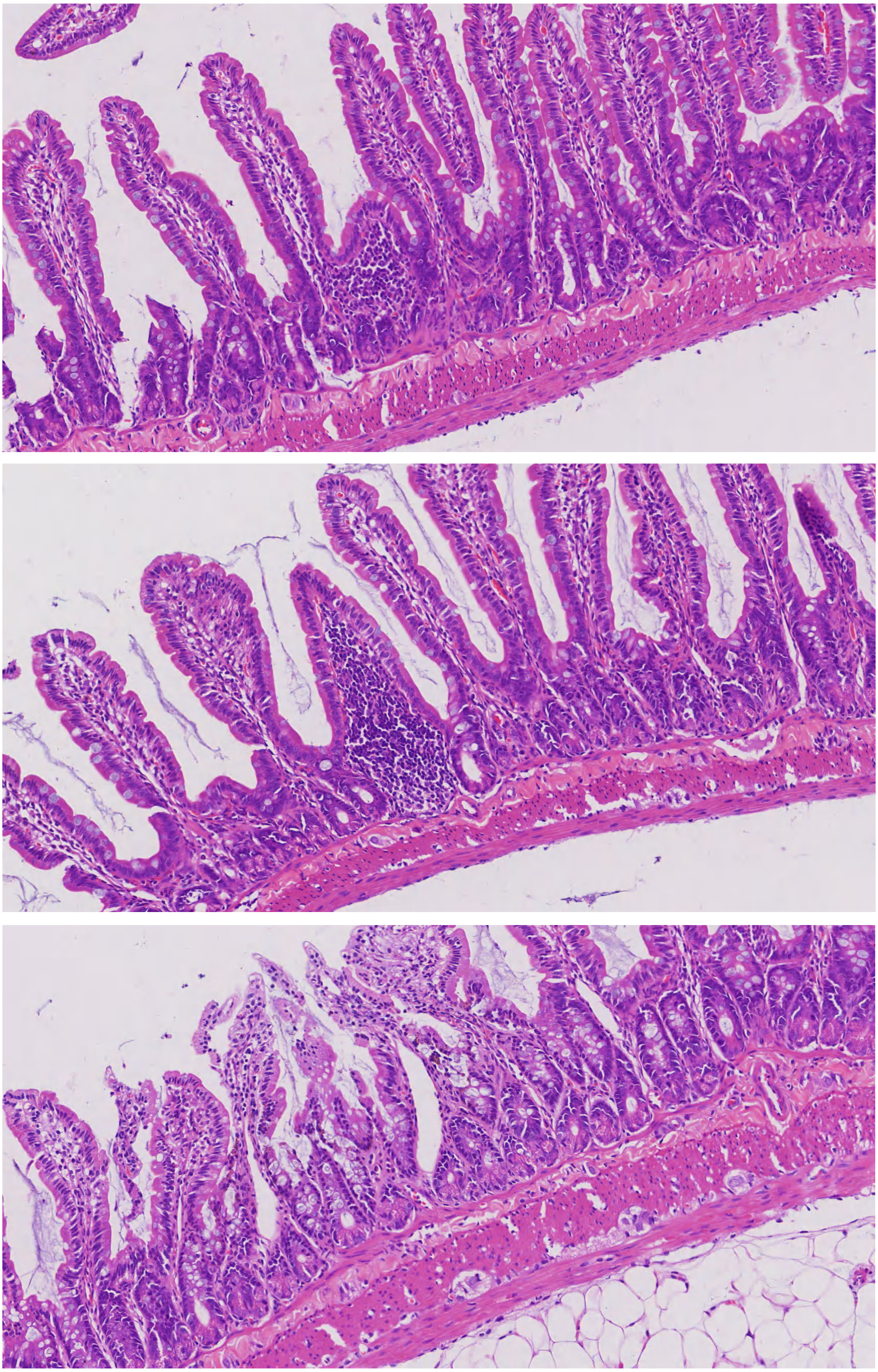

Fig.1A for CLP group (Magnification×400)

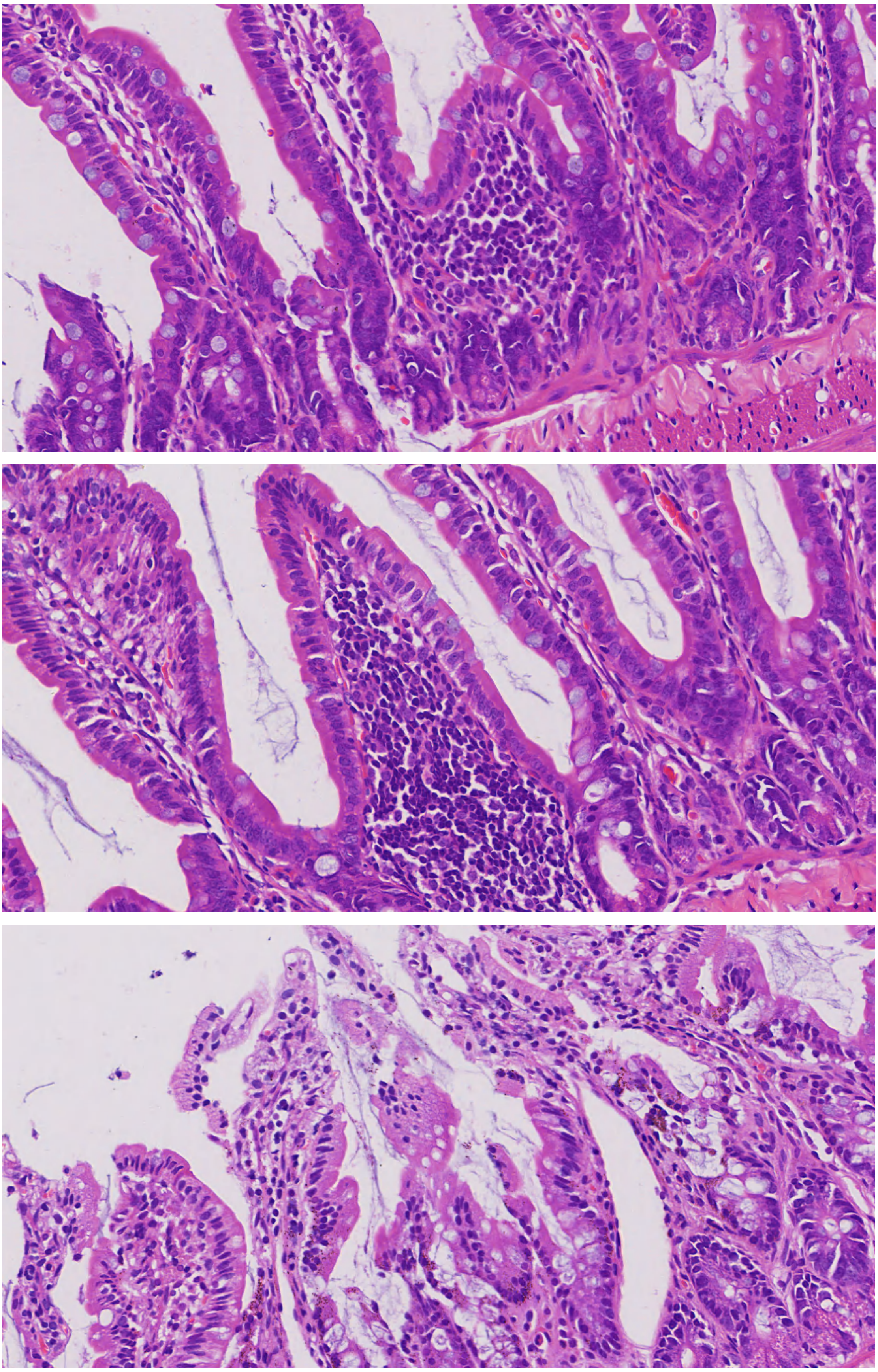

Fig.1A for CLP+GSH group (Magnification×200)

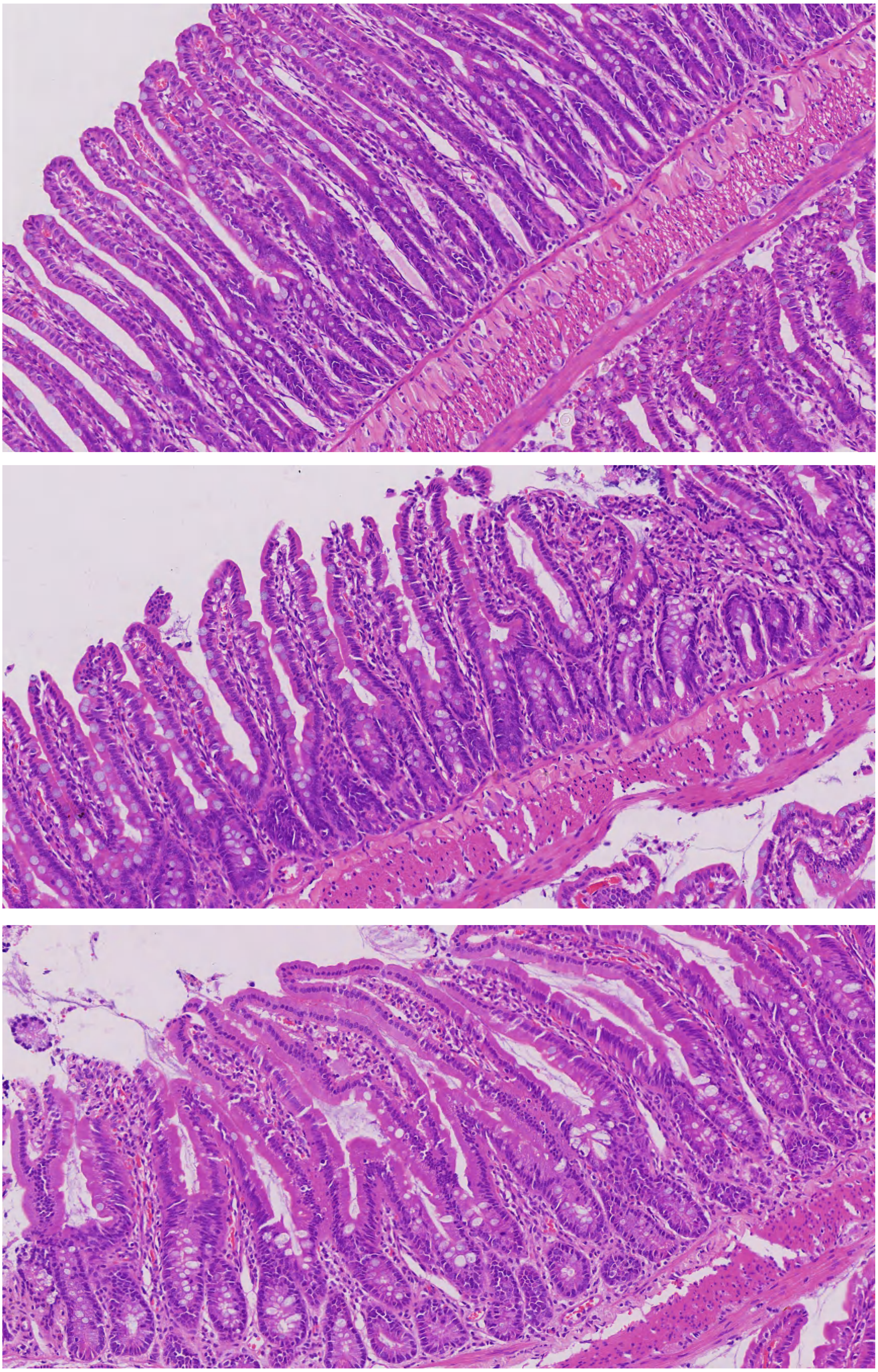

Fig.1A for CLP+GSH group (Magnification×400)

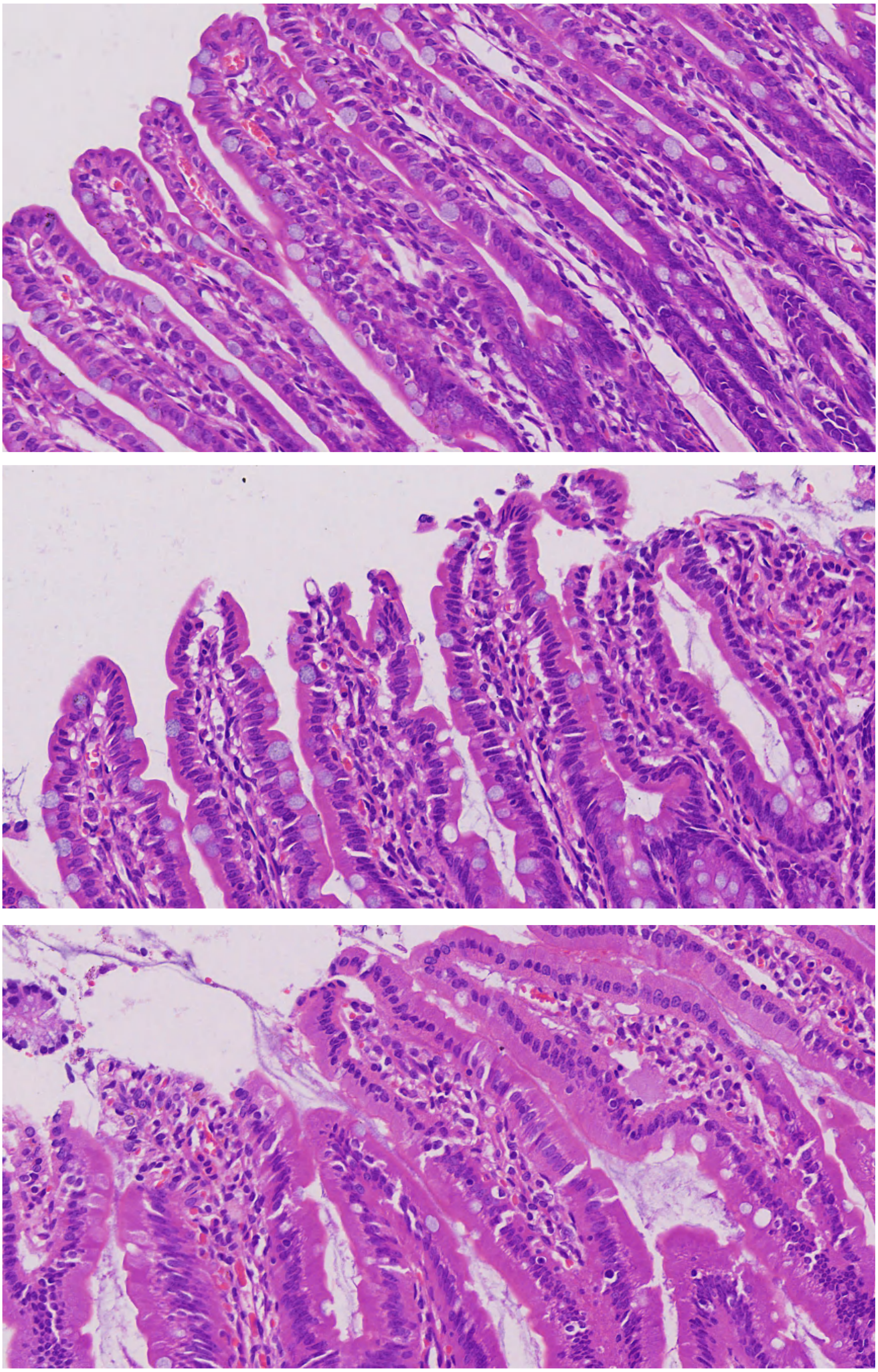

Fig.1B

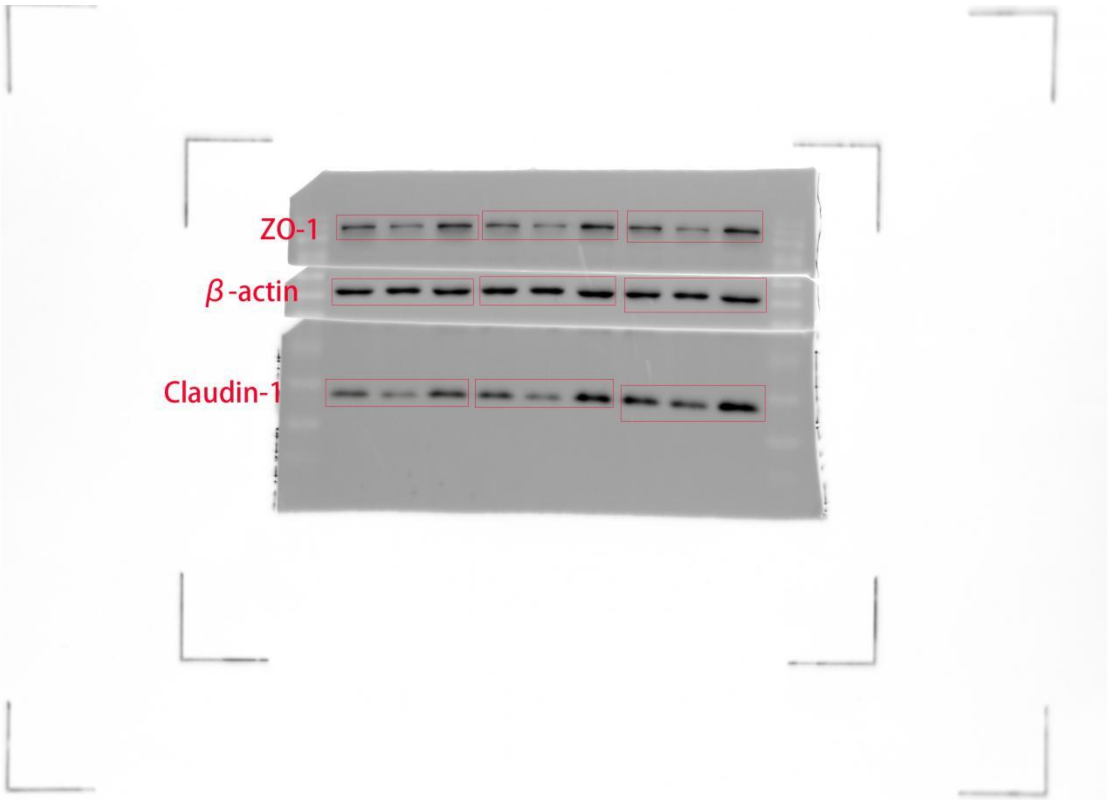
ZO-1
β-actin
Claudin-1

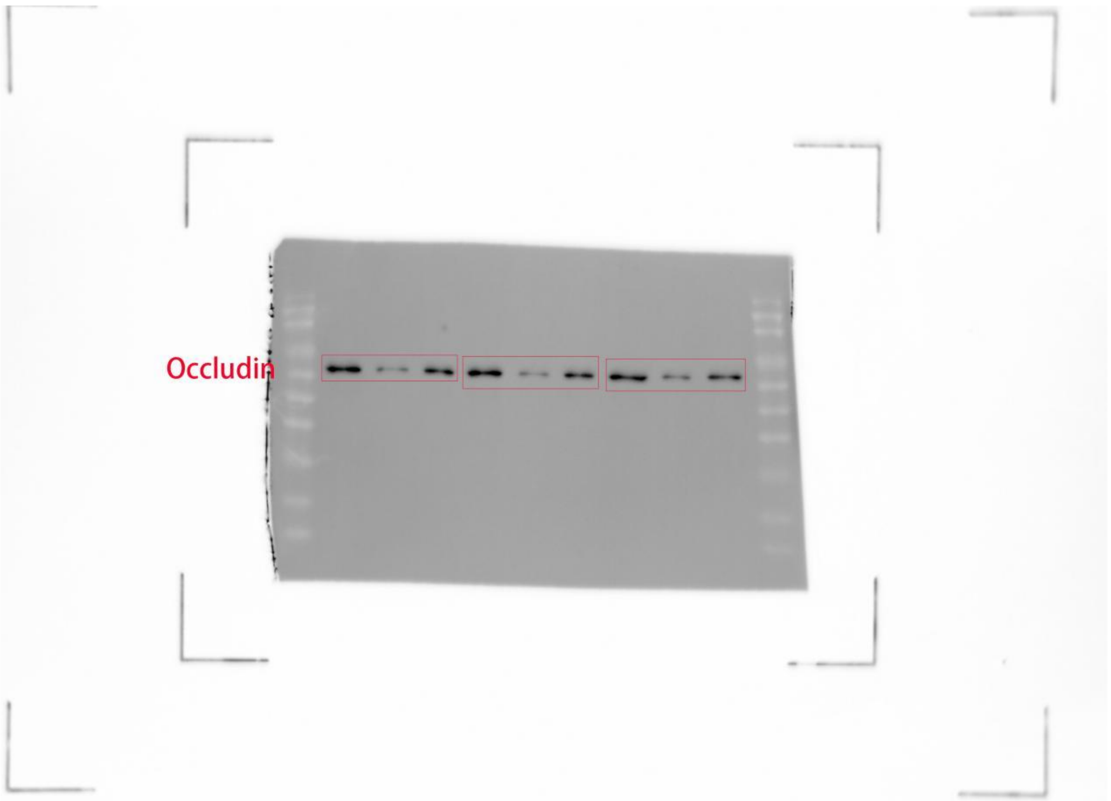
Occludin

Fig.1E

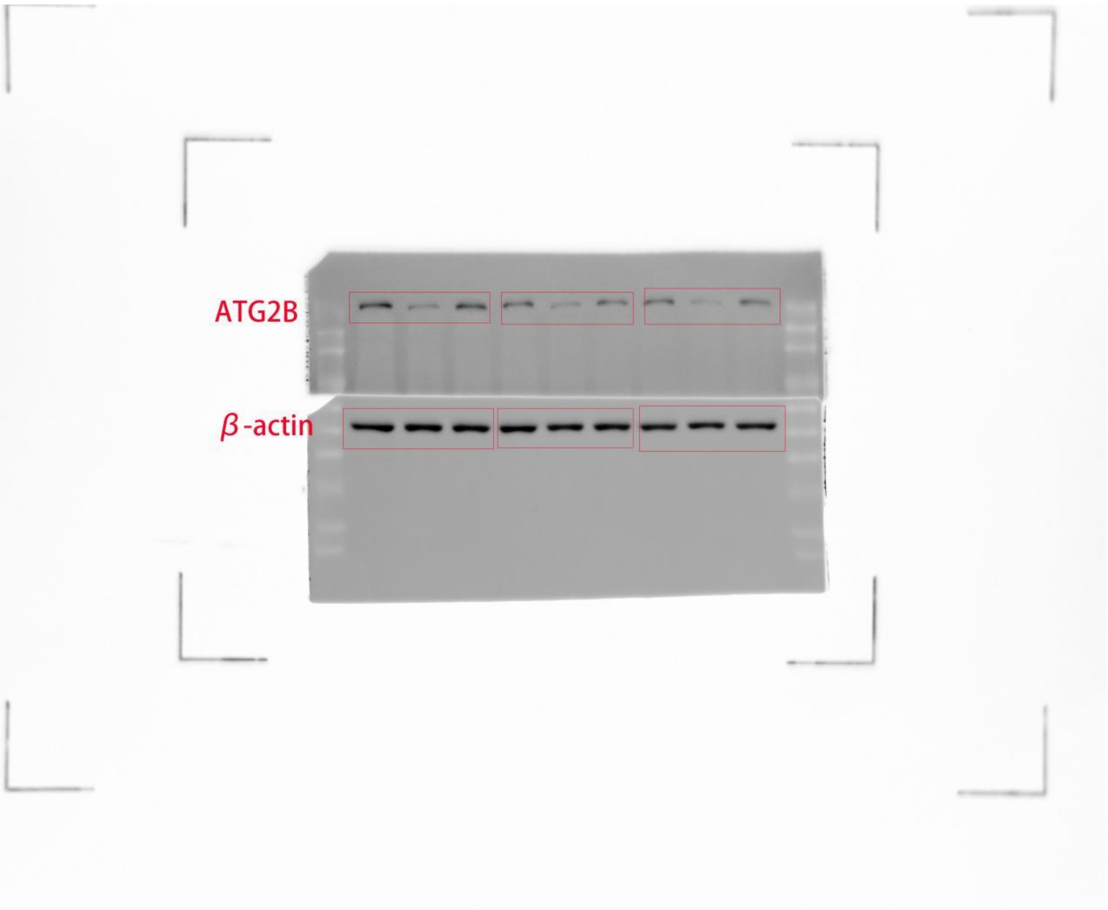

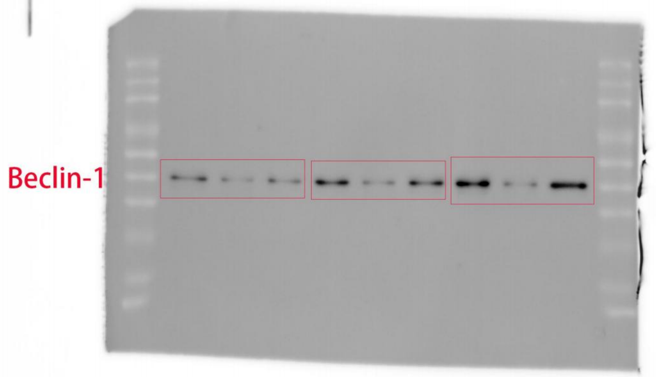
Beclin-1

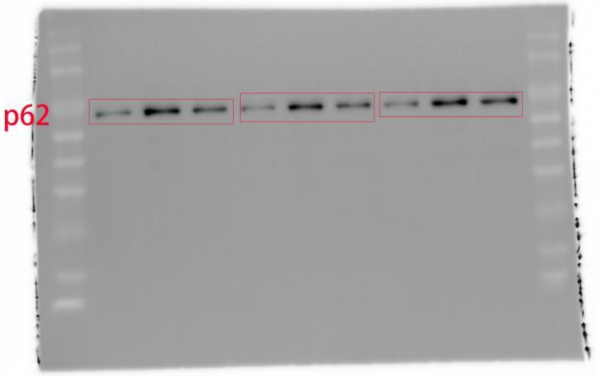
p62

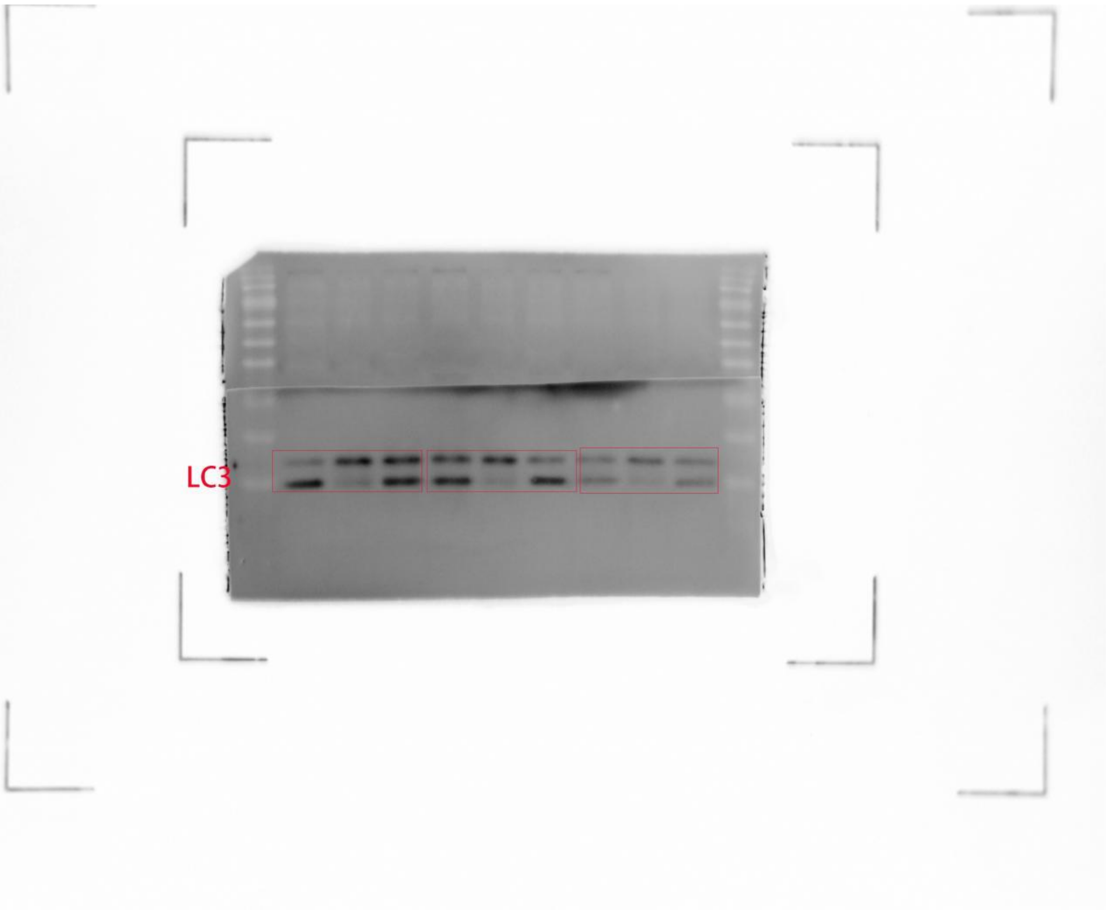

Fig.2A for CLP group (Magnification×200)

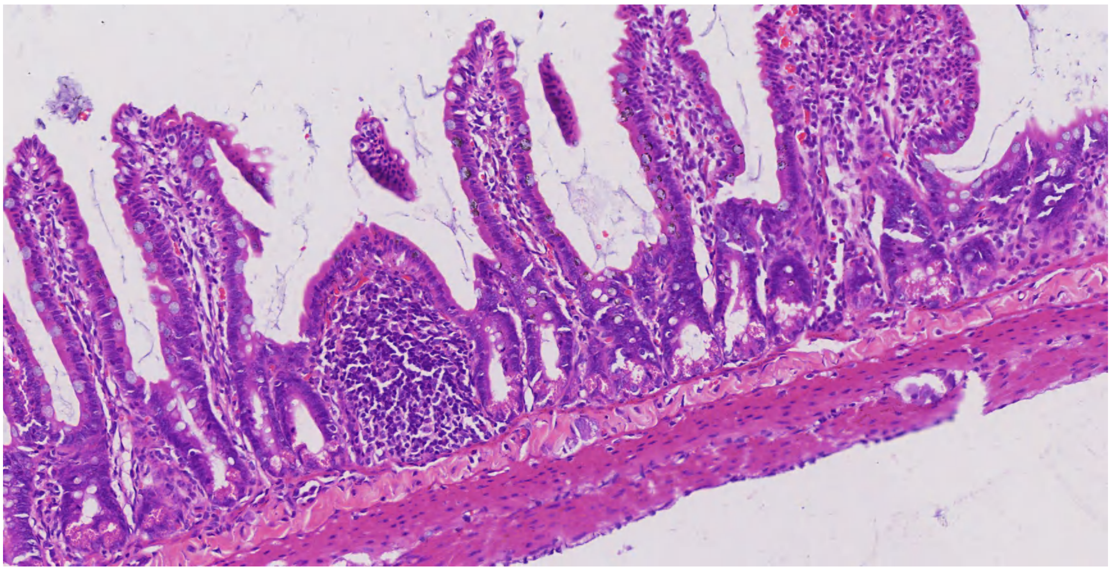

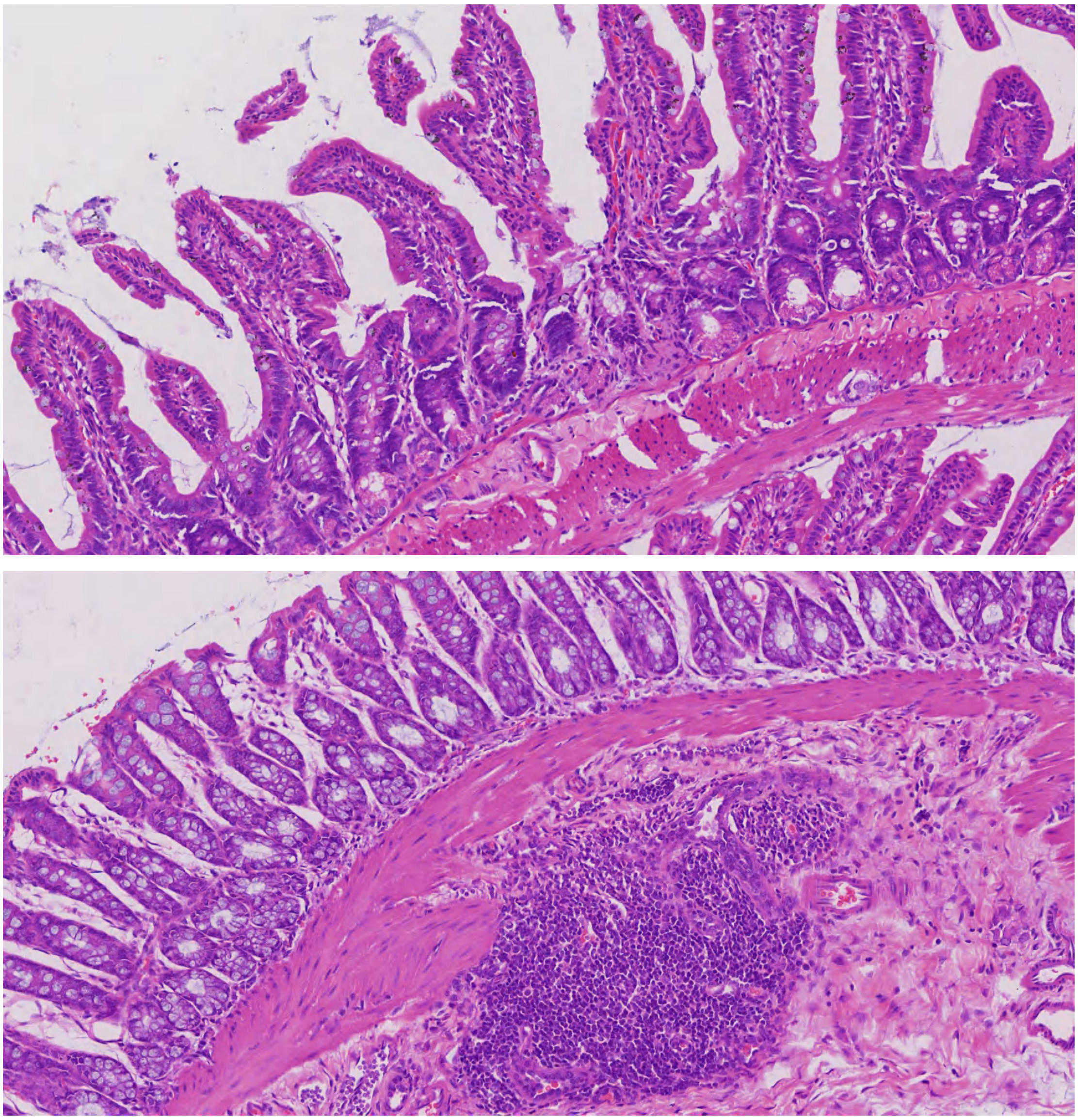

Fig.2A for CLP group (Magnification×400)

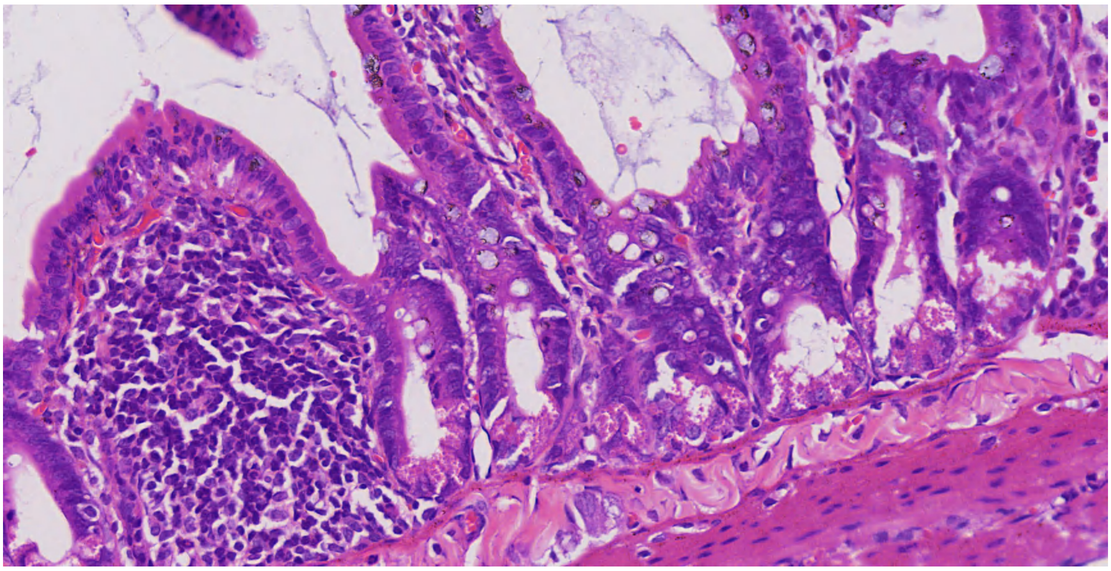

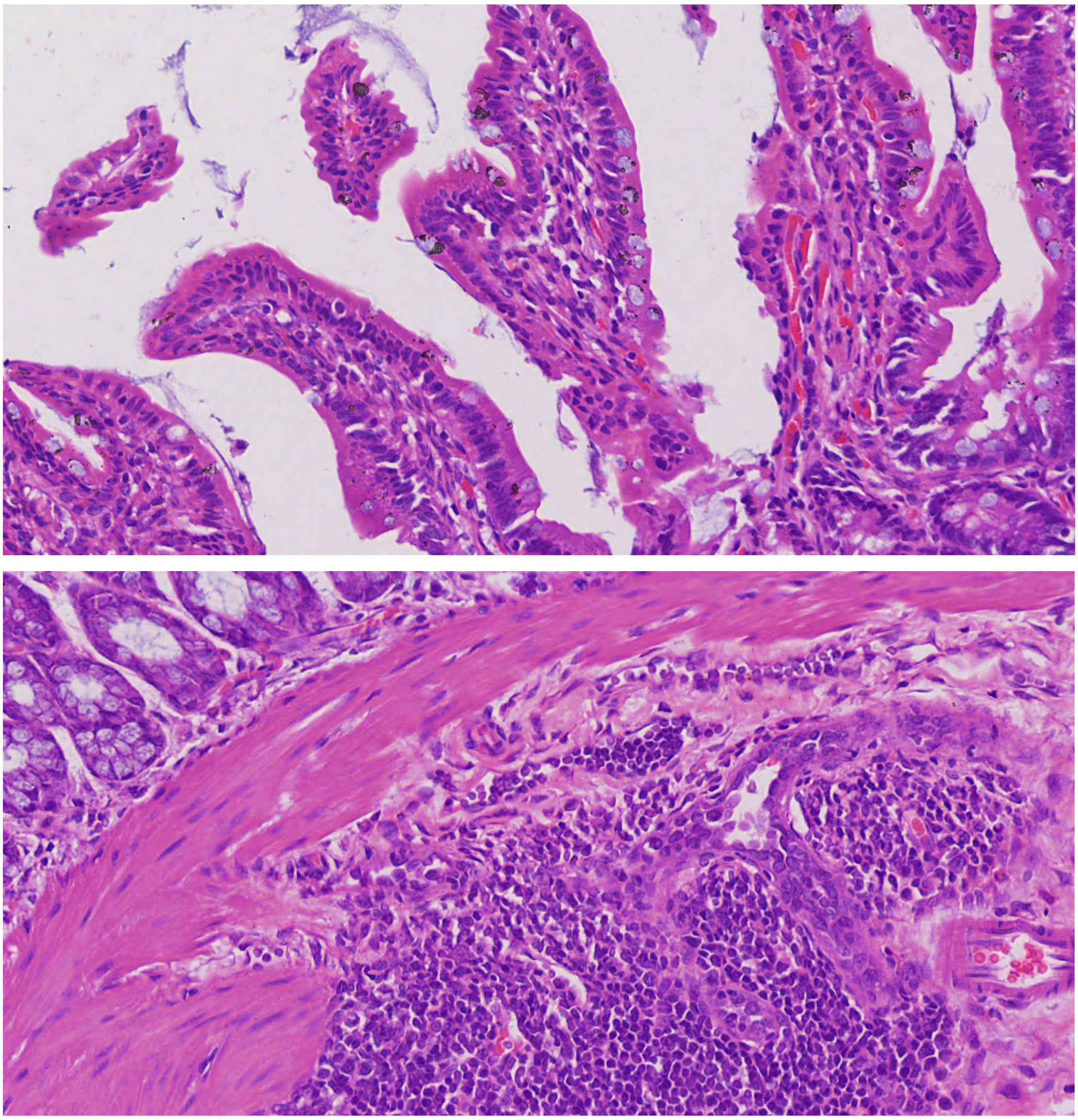

Fig.2A for CLP+LV-NC group (Magnification×200)

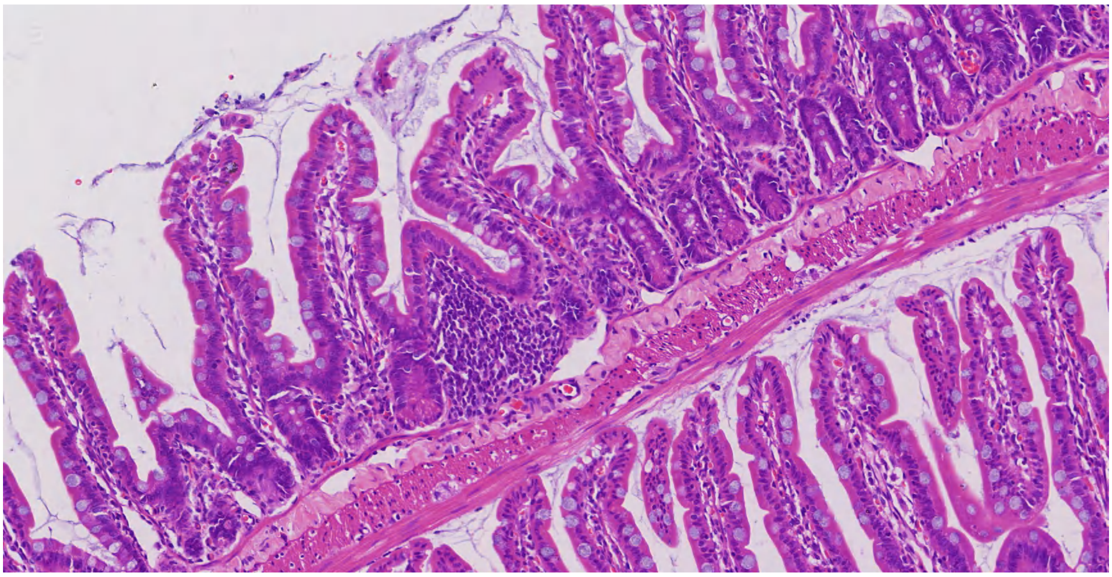

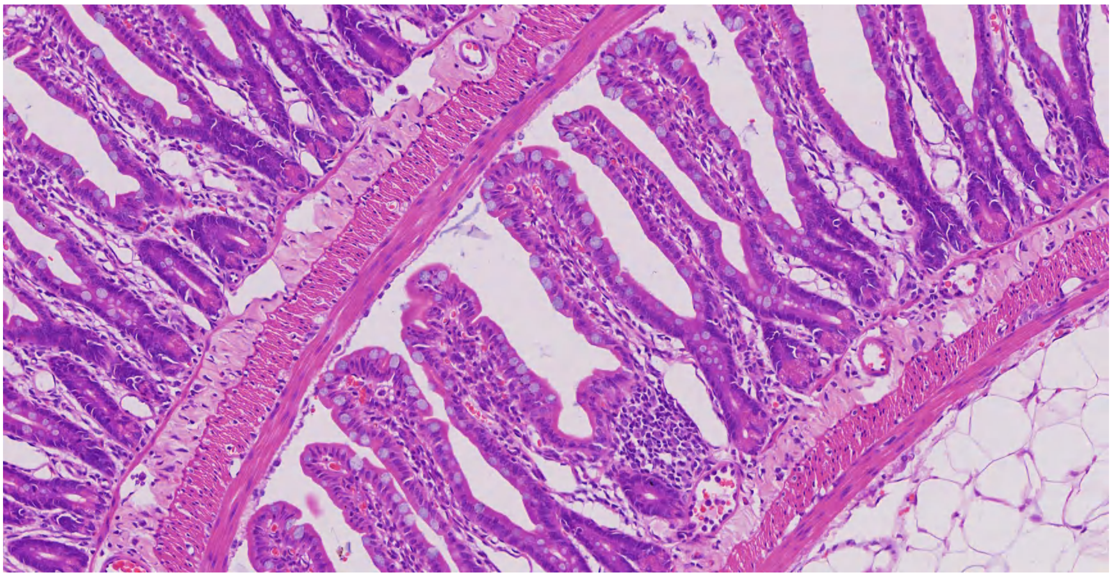

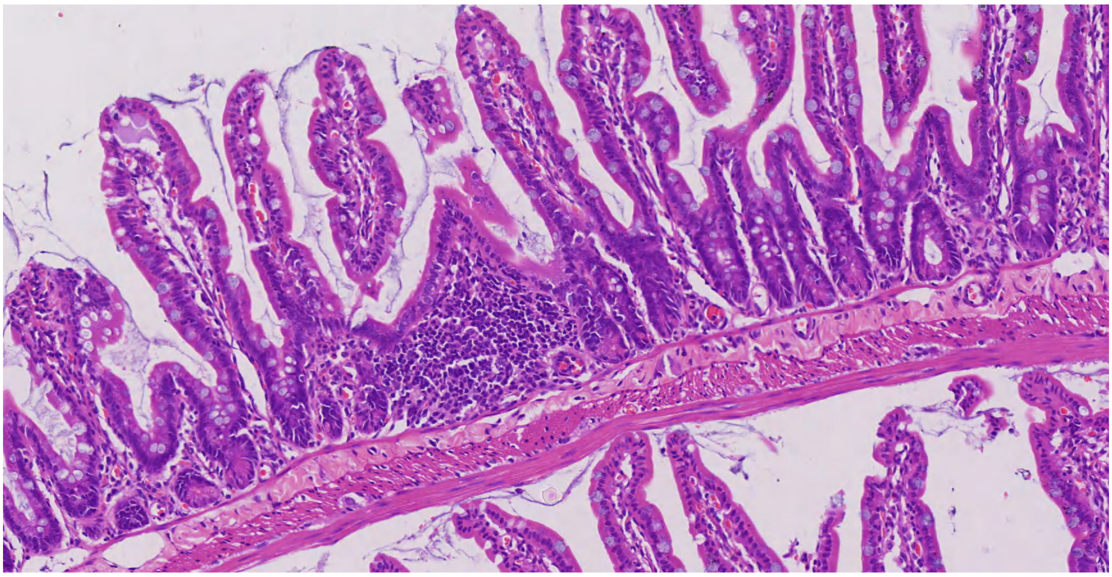

Fig.2A for CLP+LV-NC group (Magnification×400)

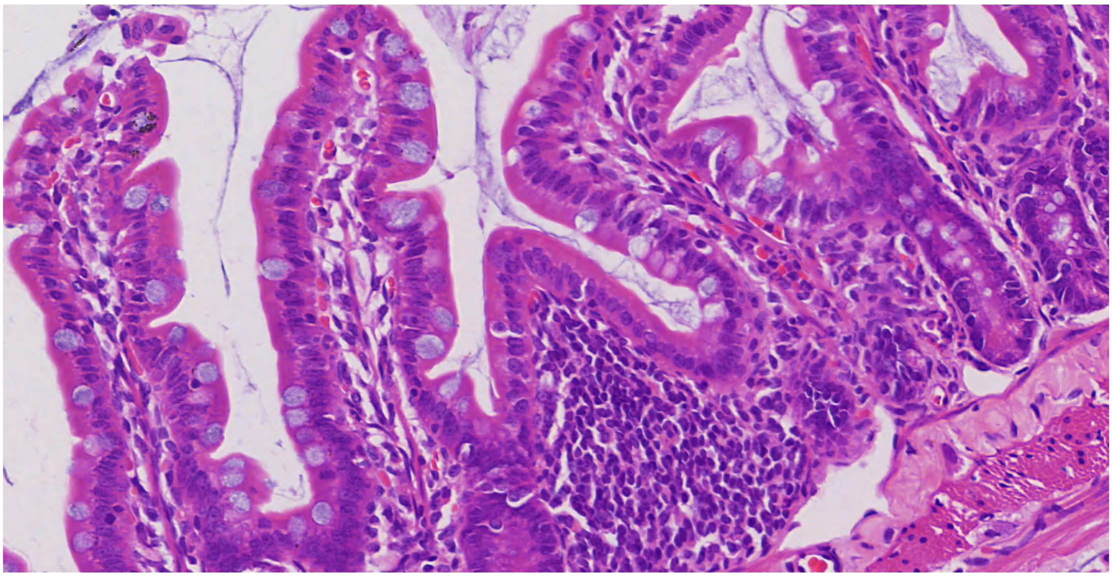

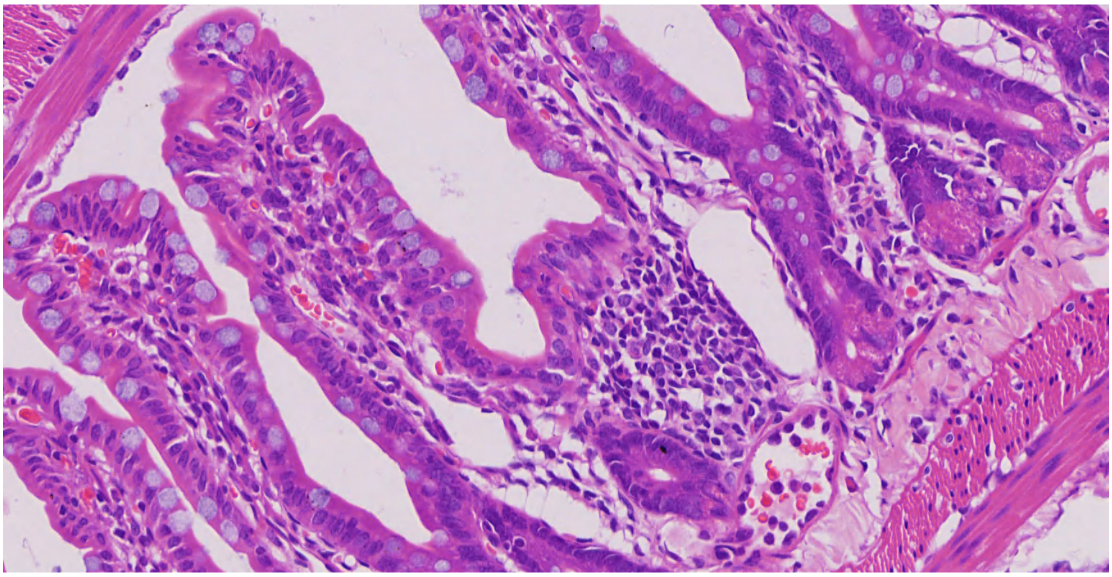

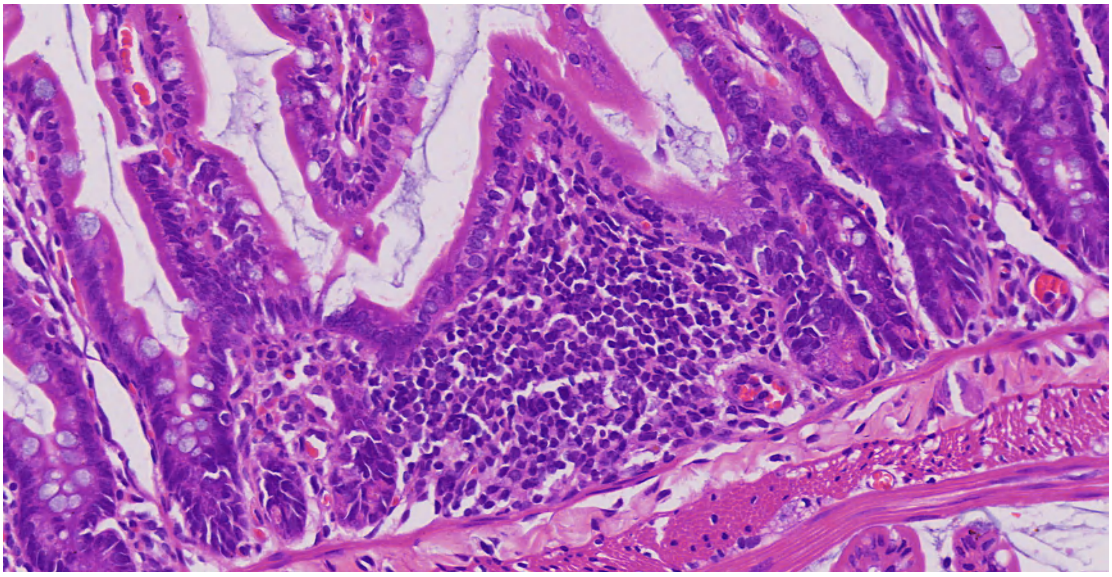

Fig.2A for CLP+LV-miR-143 group (Magnification×200)

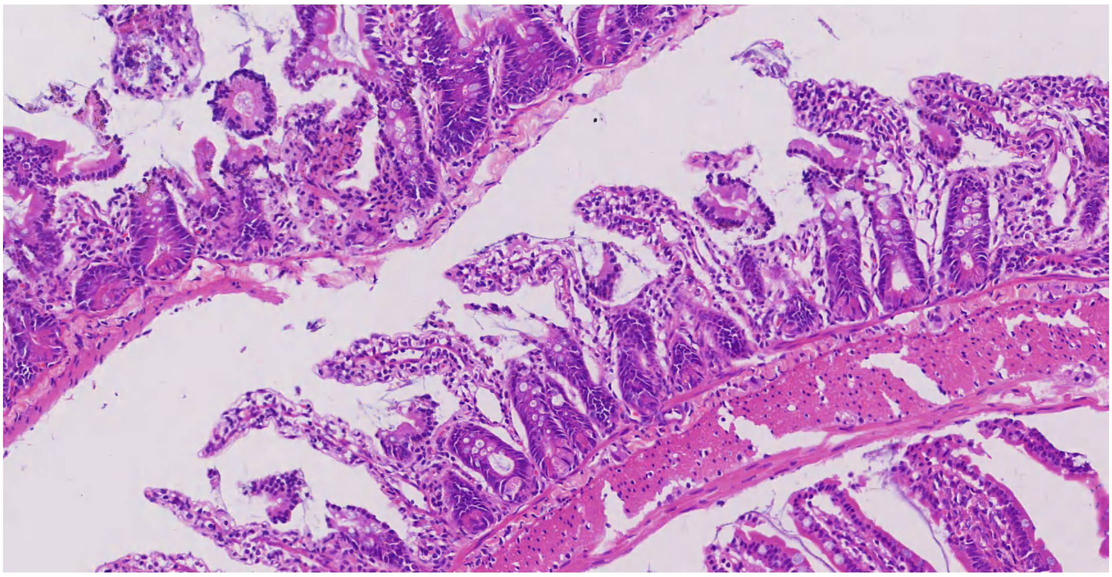

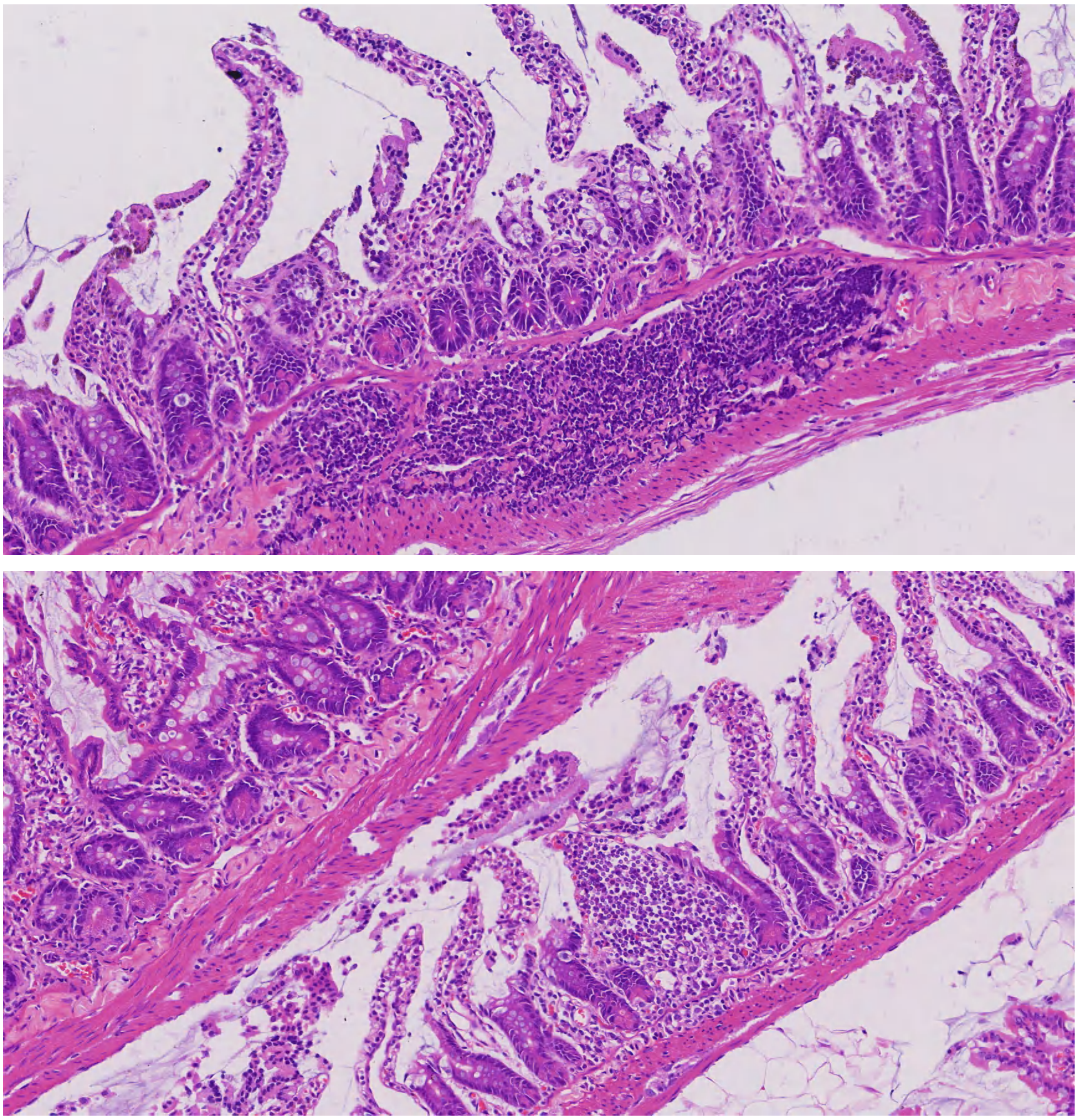

Fig.2A for CLP+LV-miR-143 group (Magnification×400)

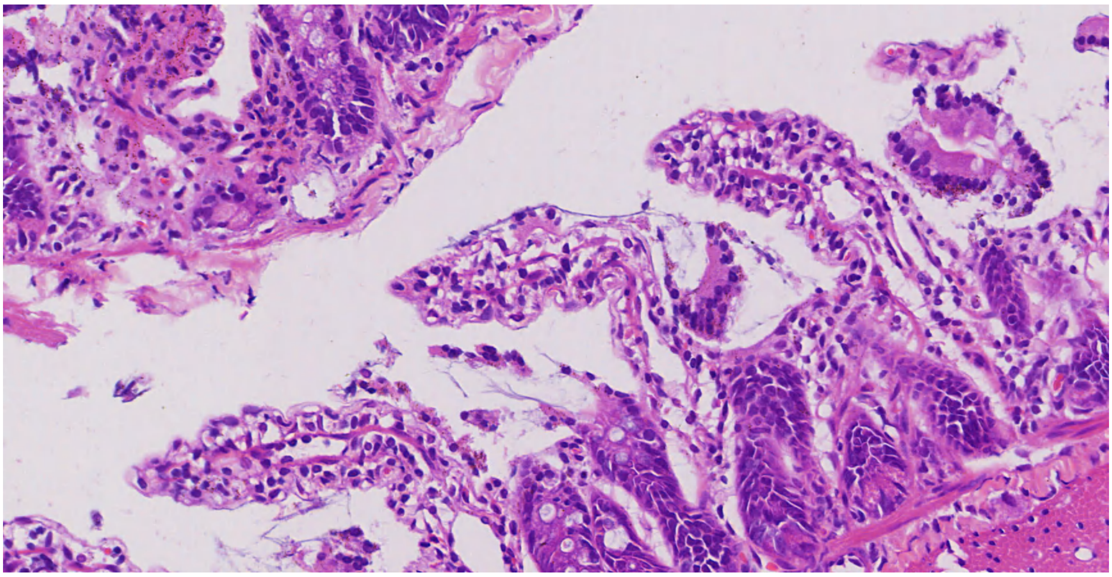

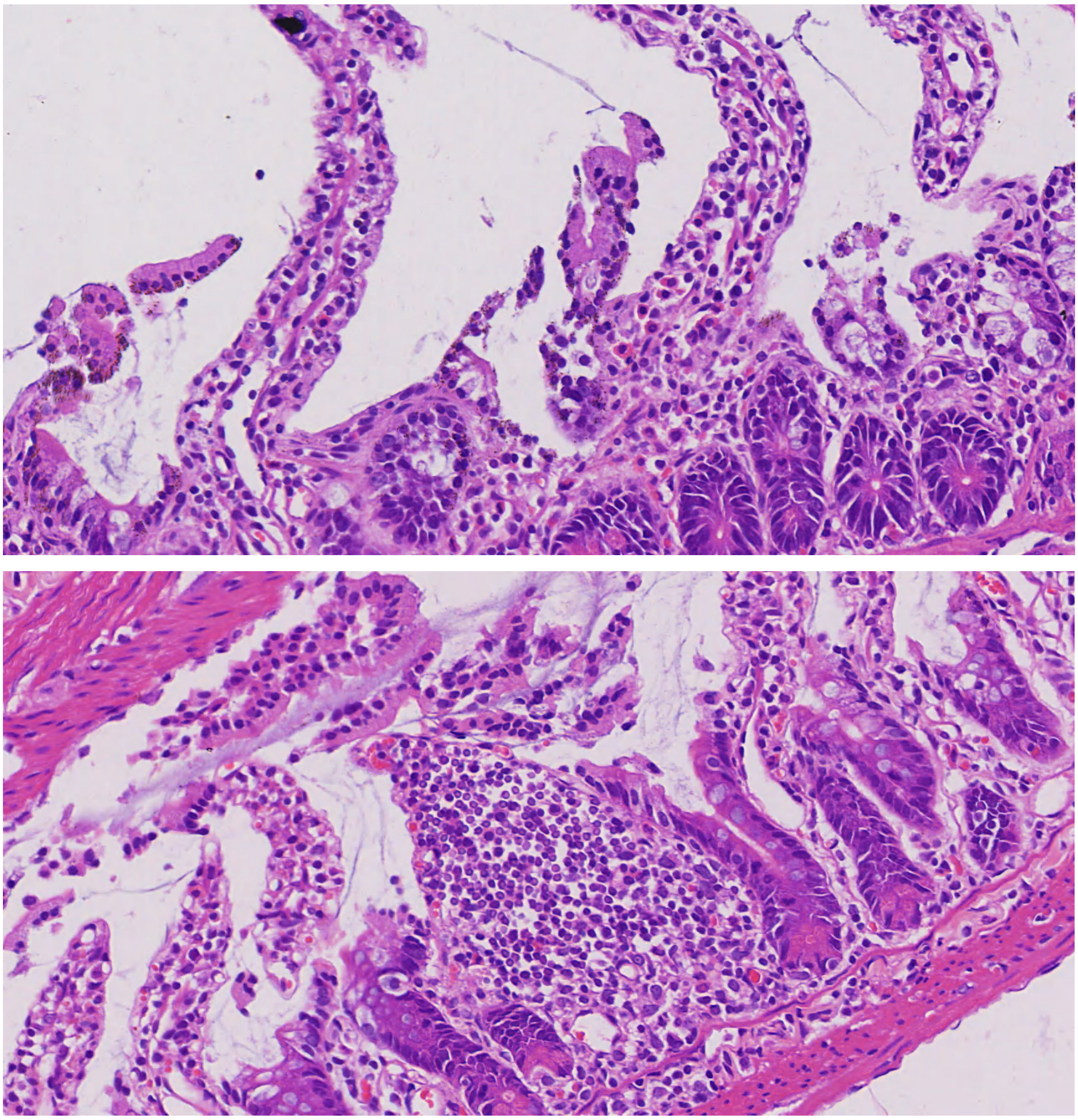

Fig.2A for CLP+LV-NC+GHS group (Magnification×200)

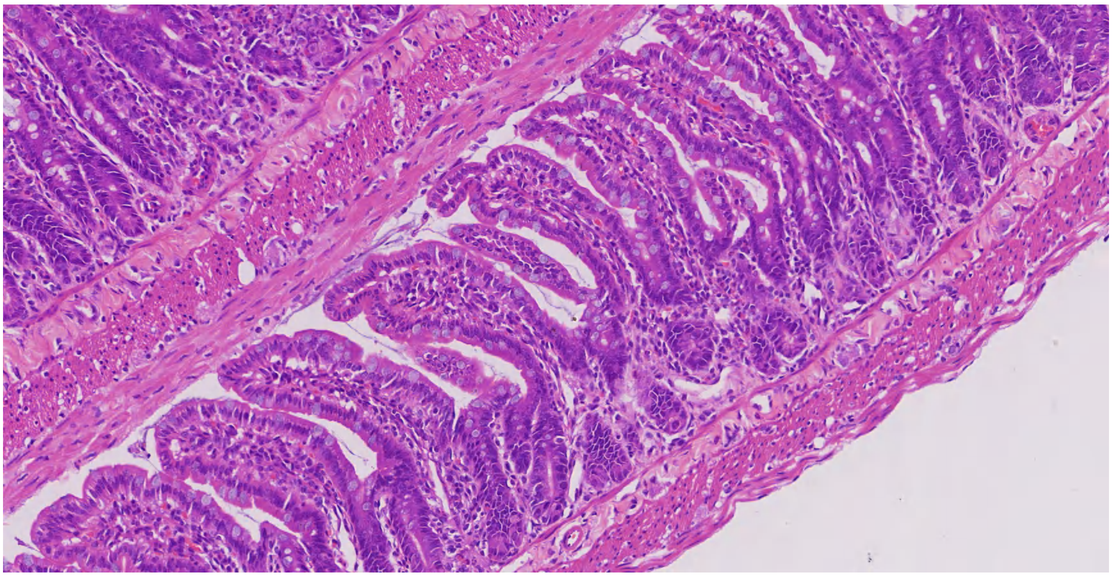

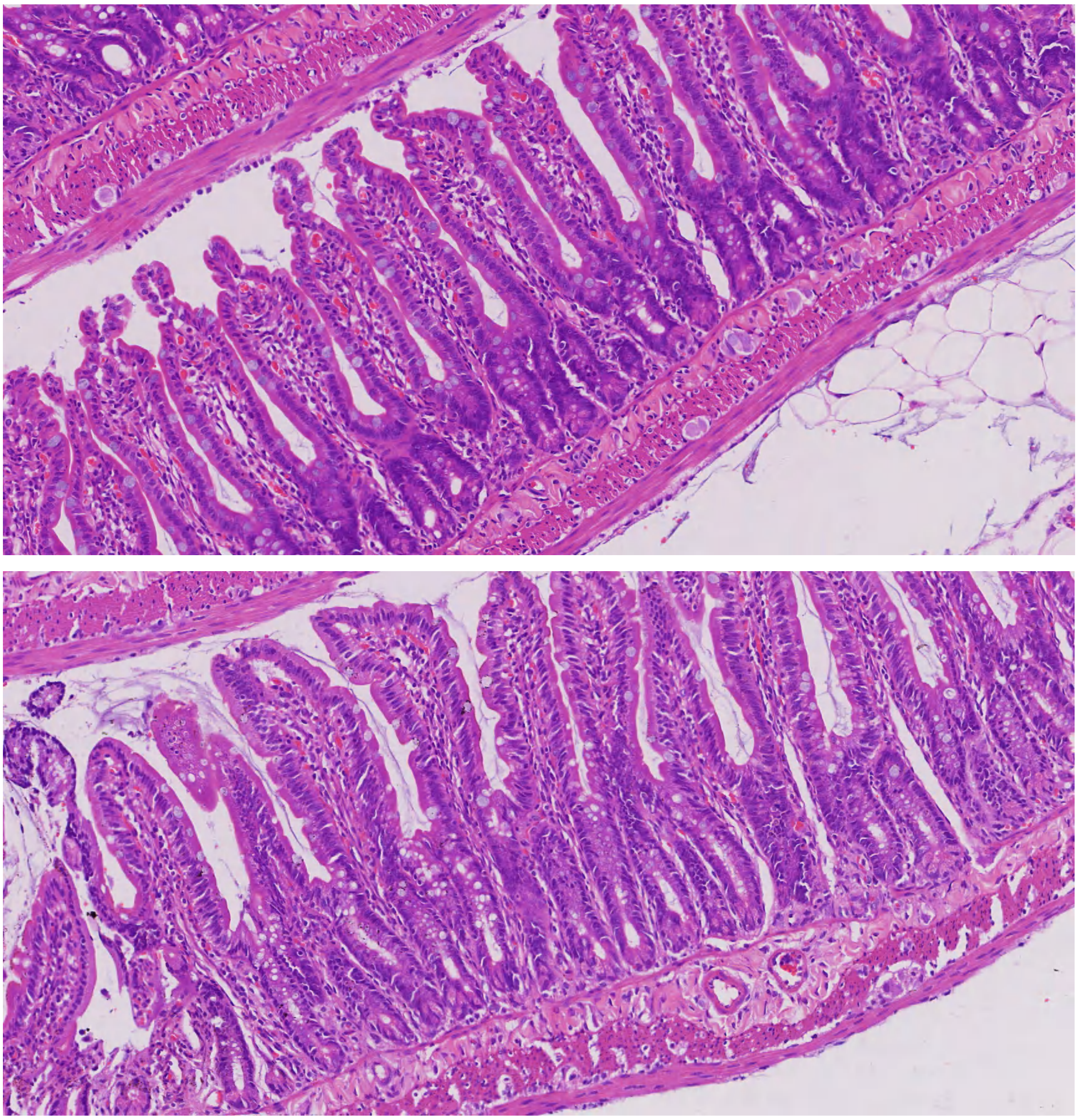

Fig.2A for CLP+LV-NC+GHS group (Magnification×400)

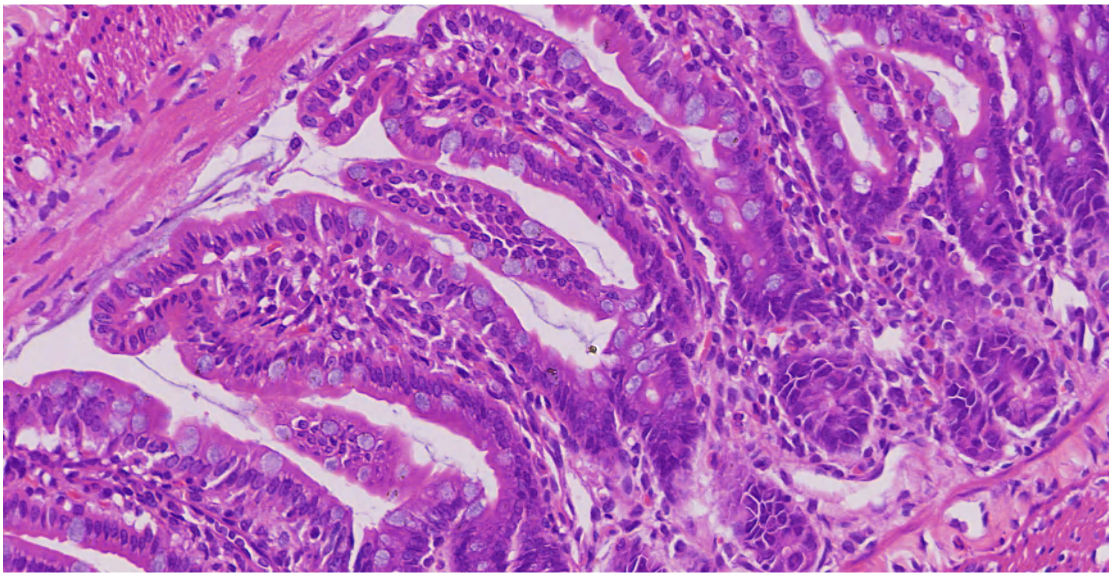

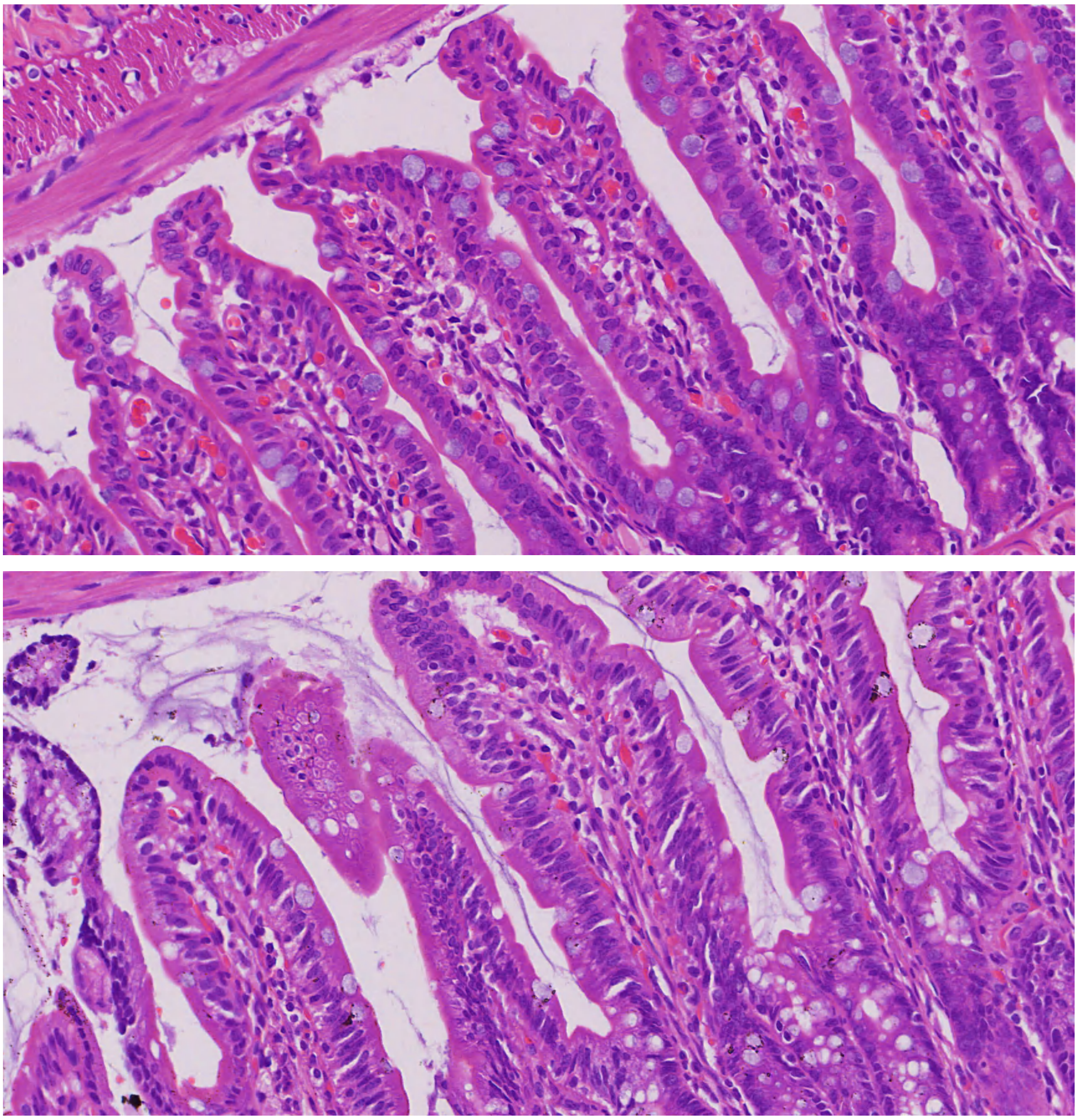

Fig.2A for CLP+LV-miR-143+GHS group (Magnification×200)

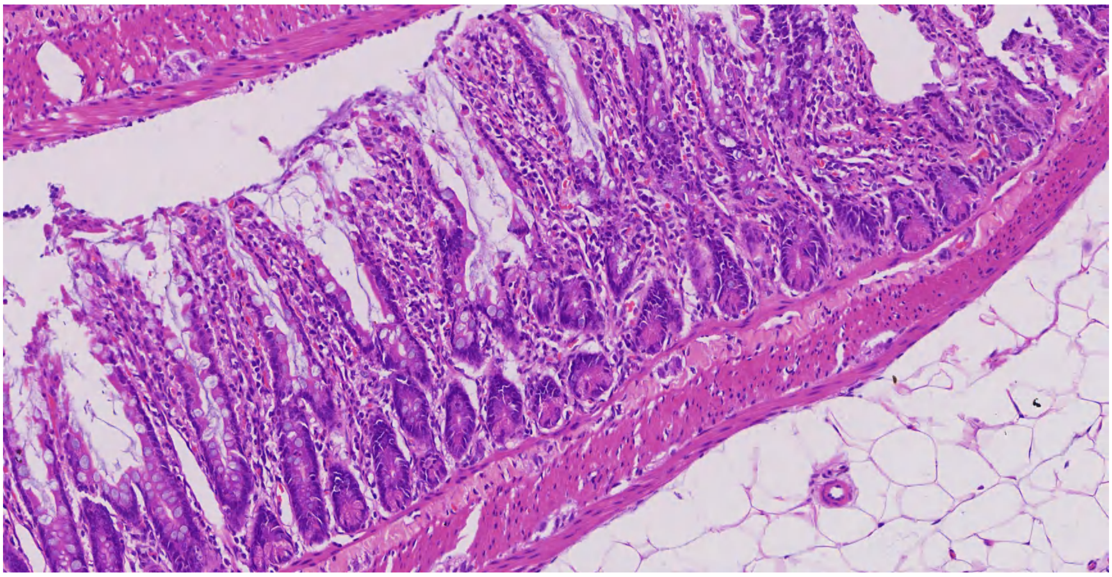

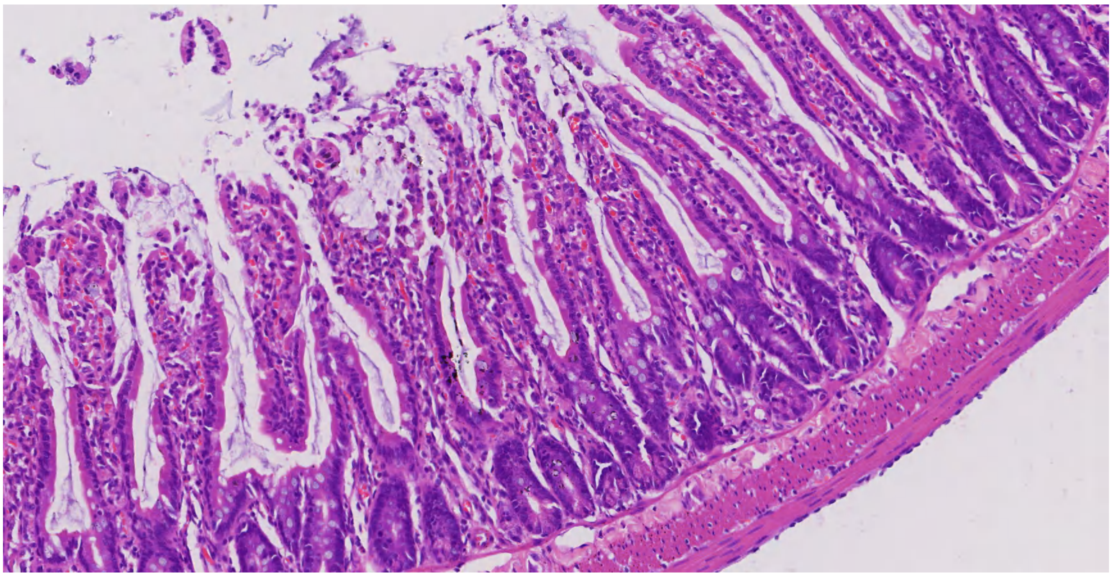

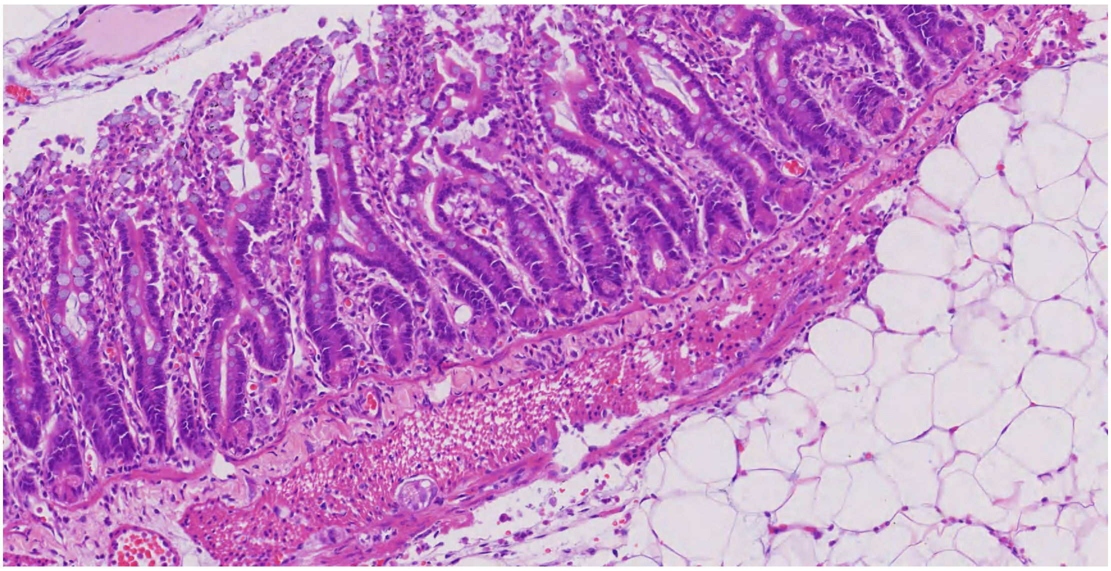

Fig.2A for CLP+LV-miR-143+GHS group (Magnification×400)

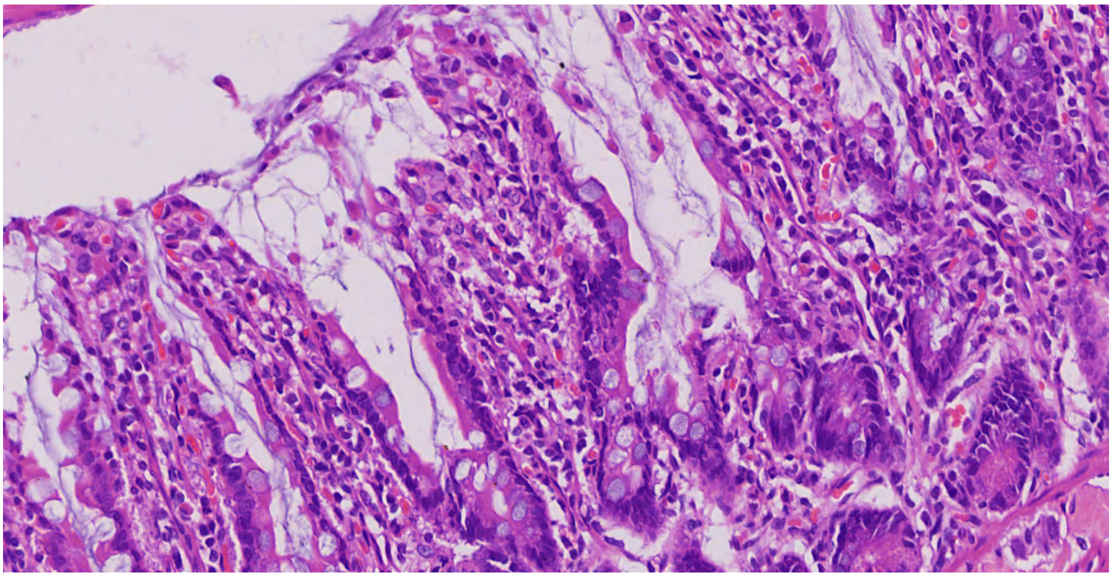

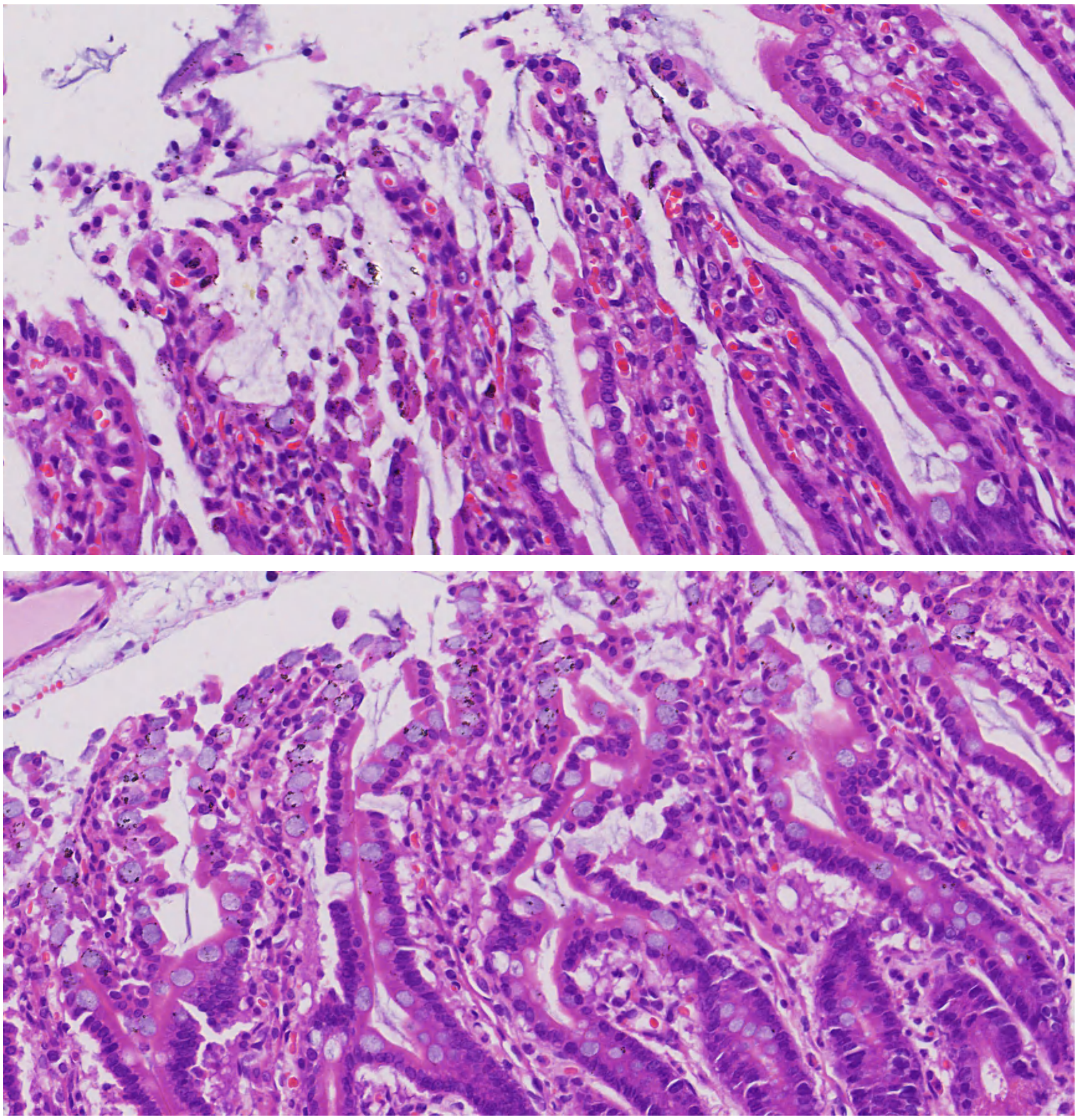

Fig.2B

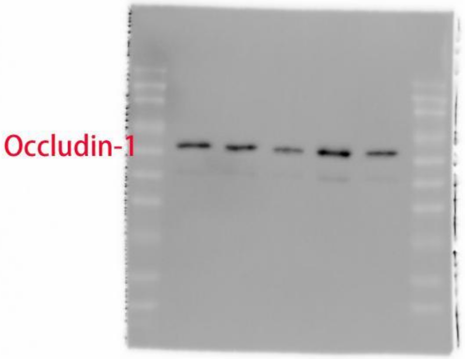
Occludin-1

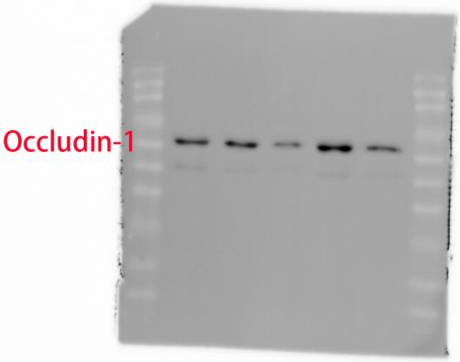
Occludin-1

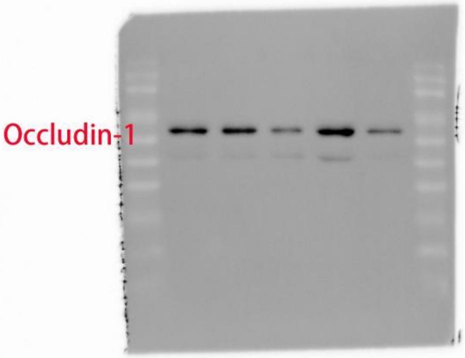
Occludin-1

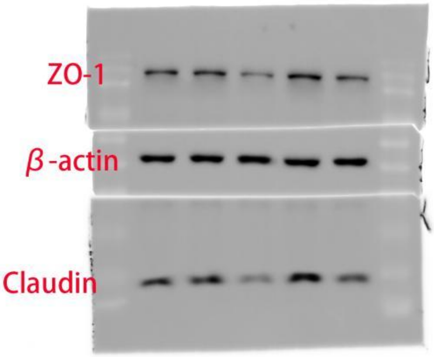
ZO-1
β-actin
Claudin

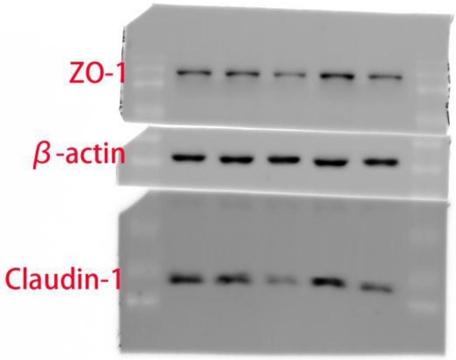
ZO-1
β-actin
Claudin-1

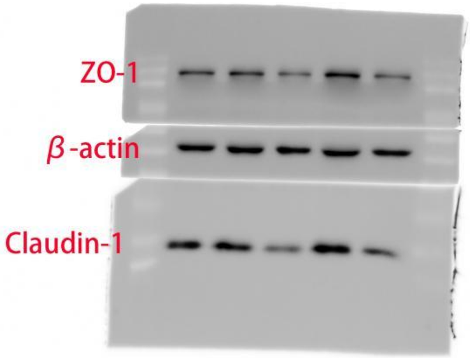
ZO-1
β-actin
Claudin-1

Fig.3A

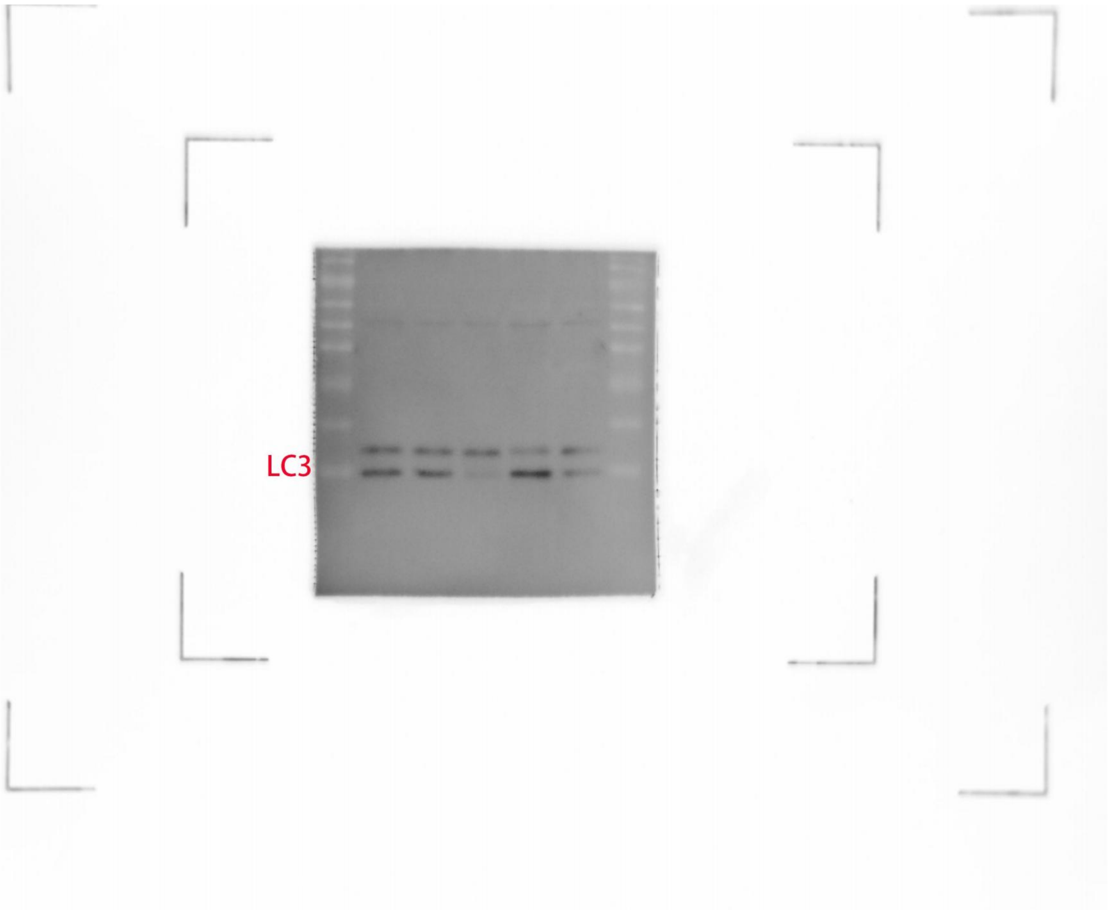

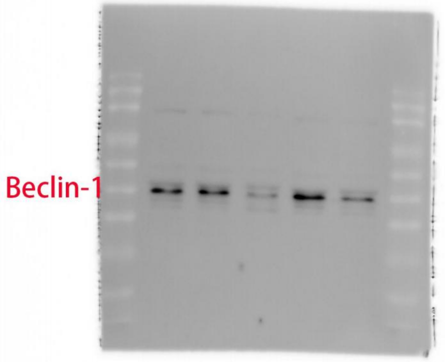
Beclin-1

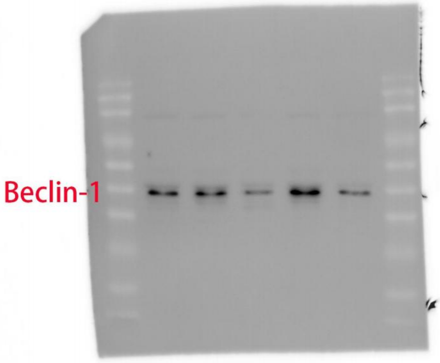
Beclin-1

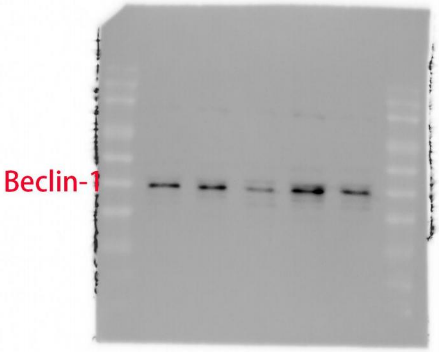
Beclin-1

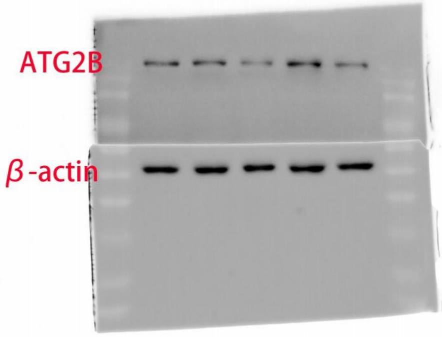
ATG2B
β-actin

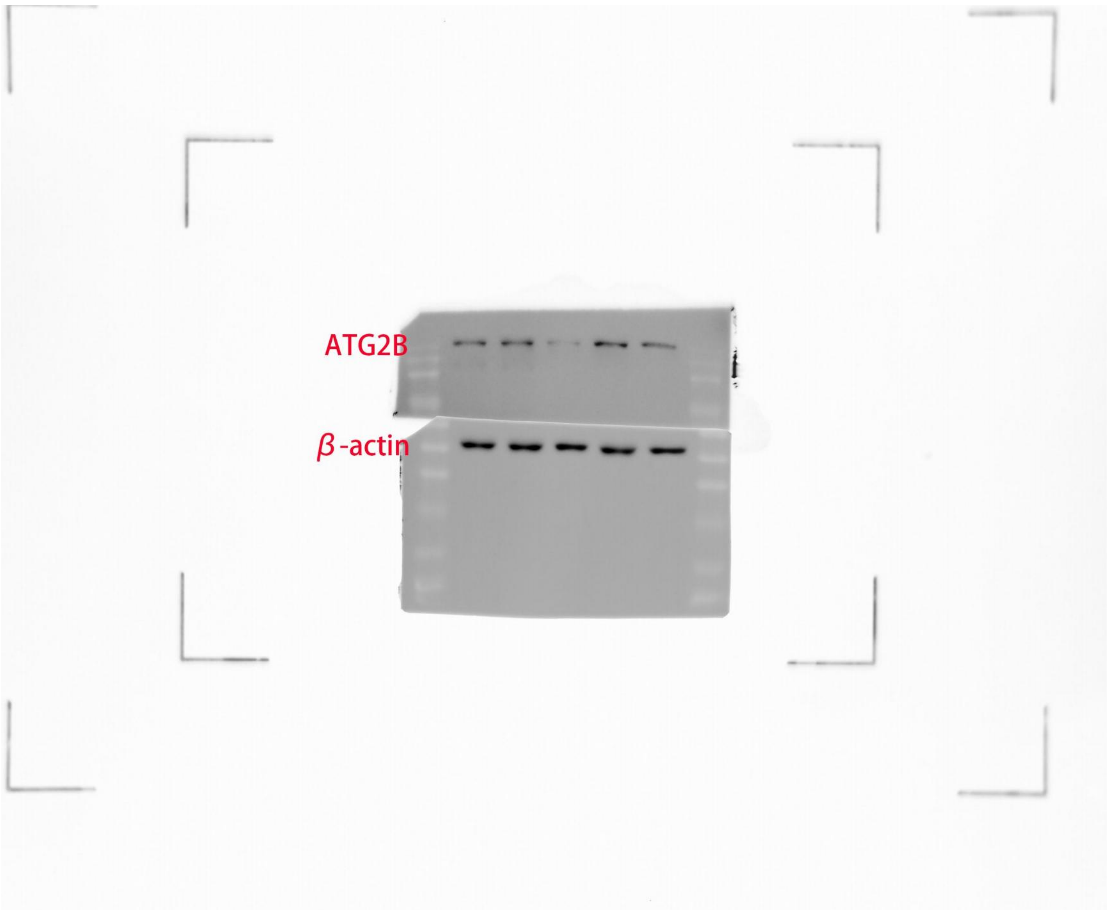
ATG2B
β-actin

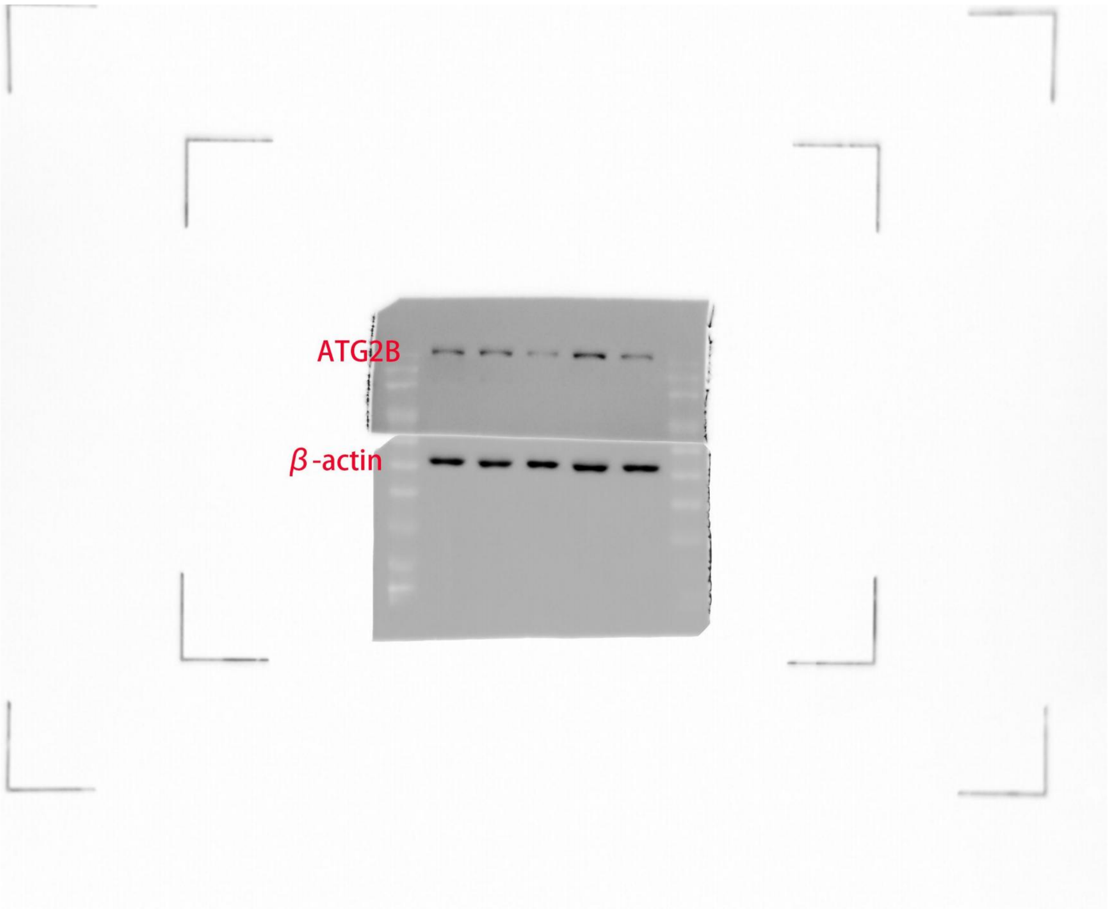
ATG2B
β-actin

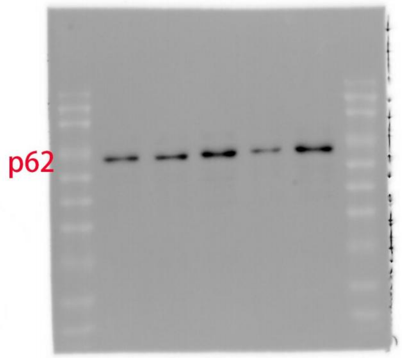
p62

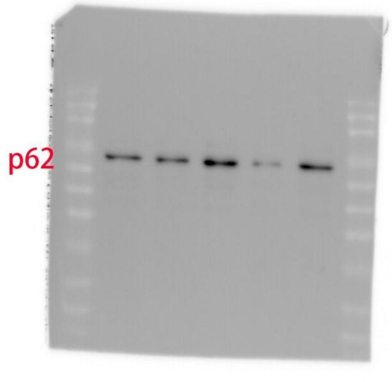
p62

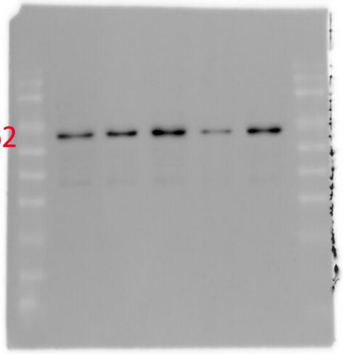
p62

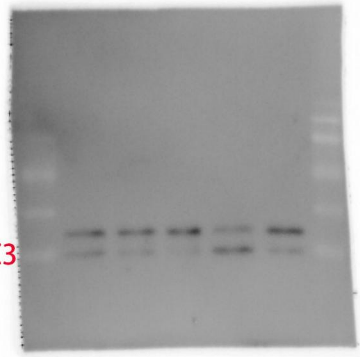
LC3

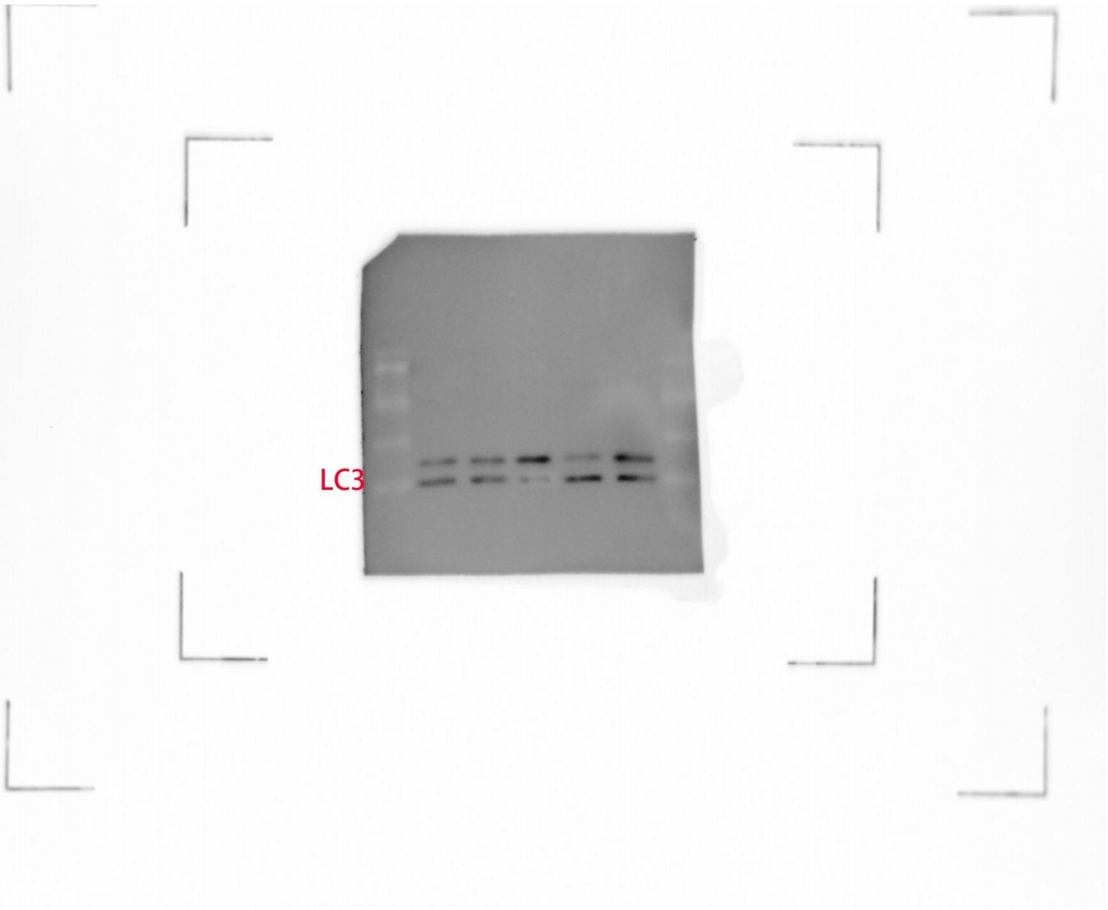

Fig.3B for the CLP group

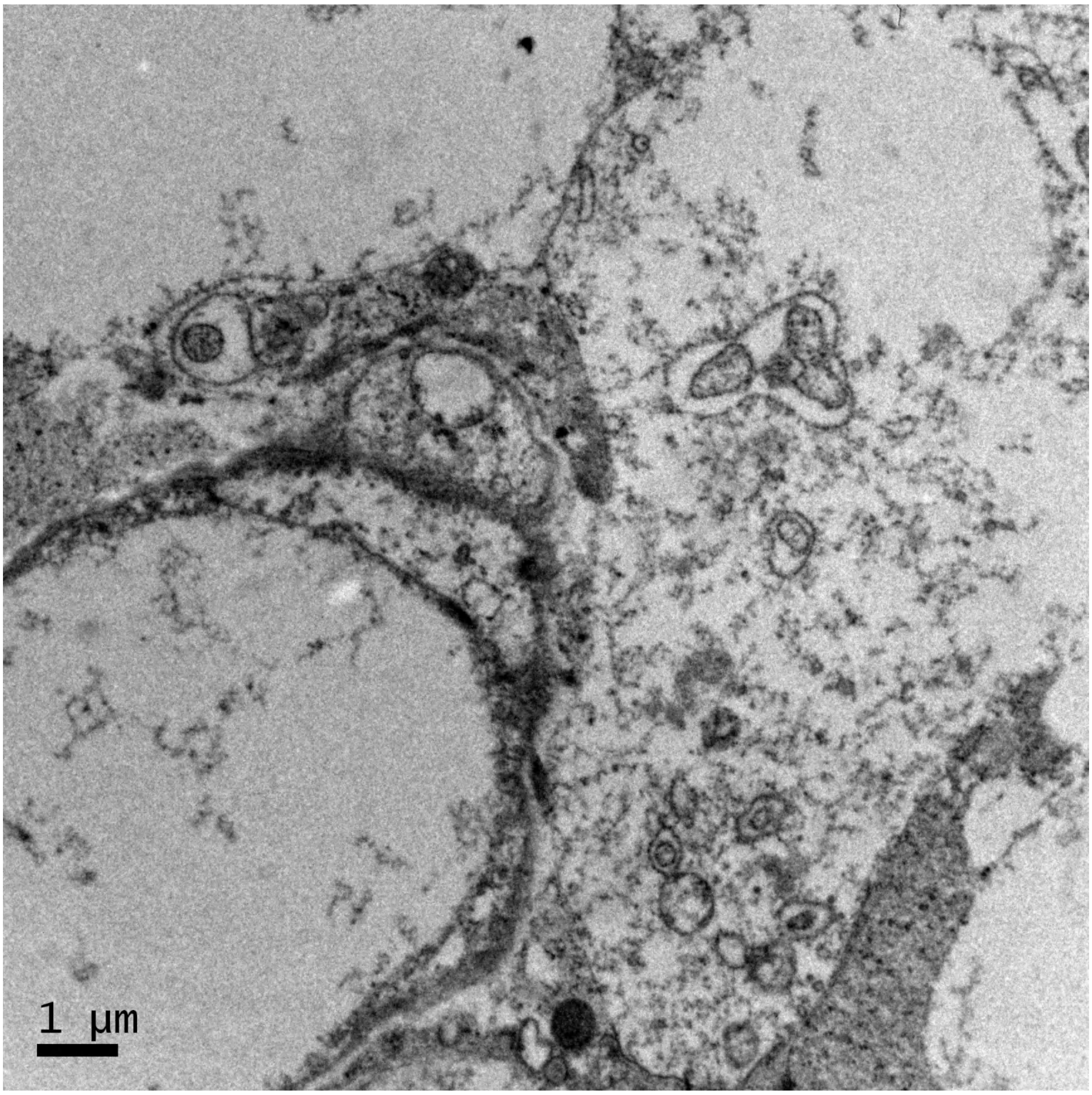
1 µm

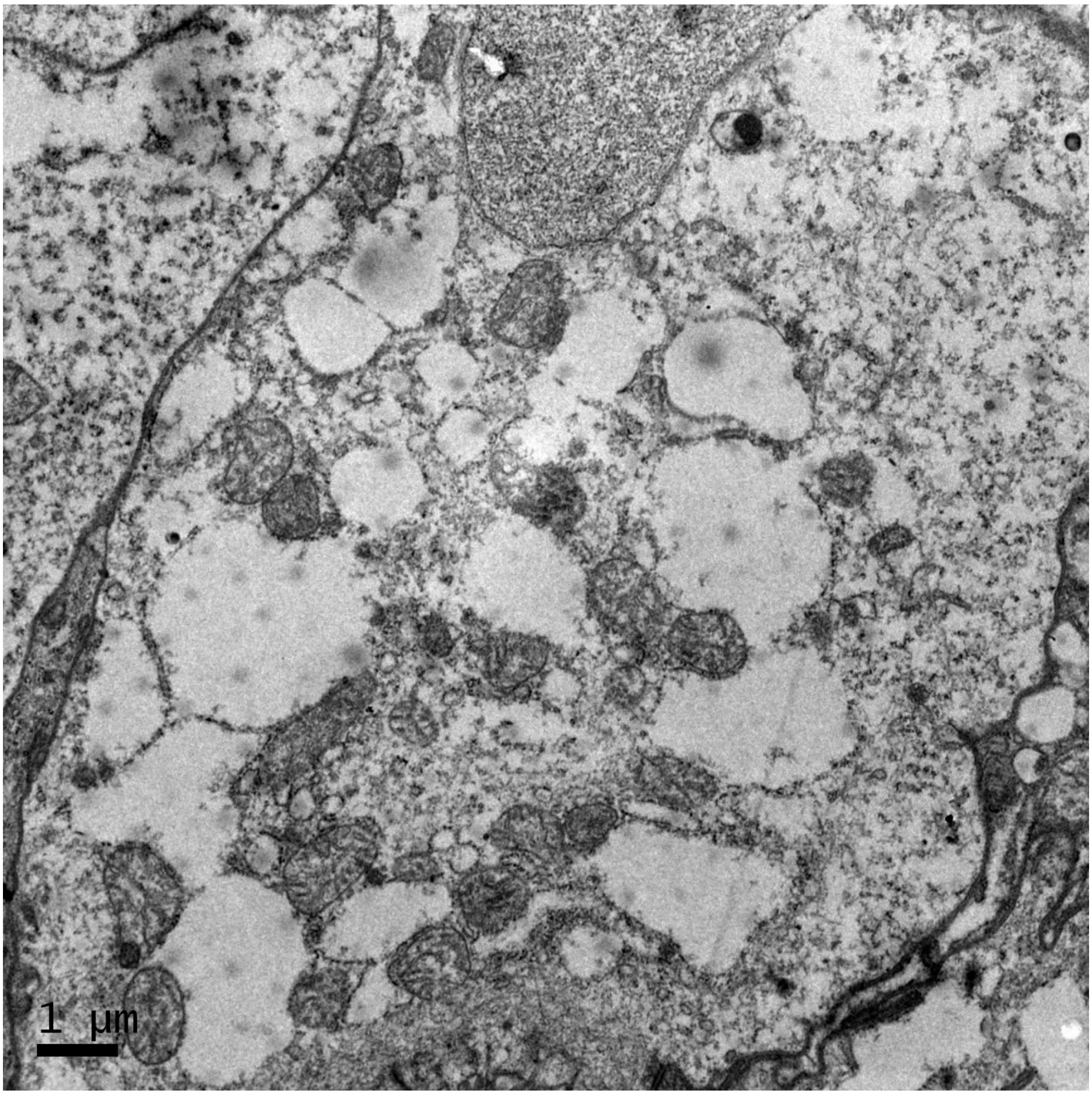
1 µm

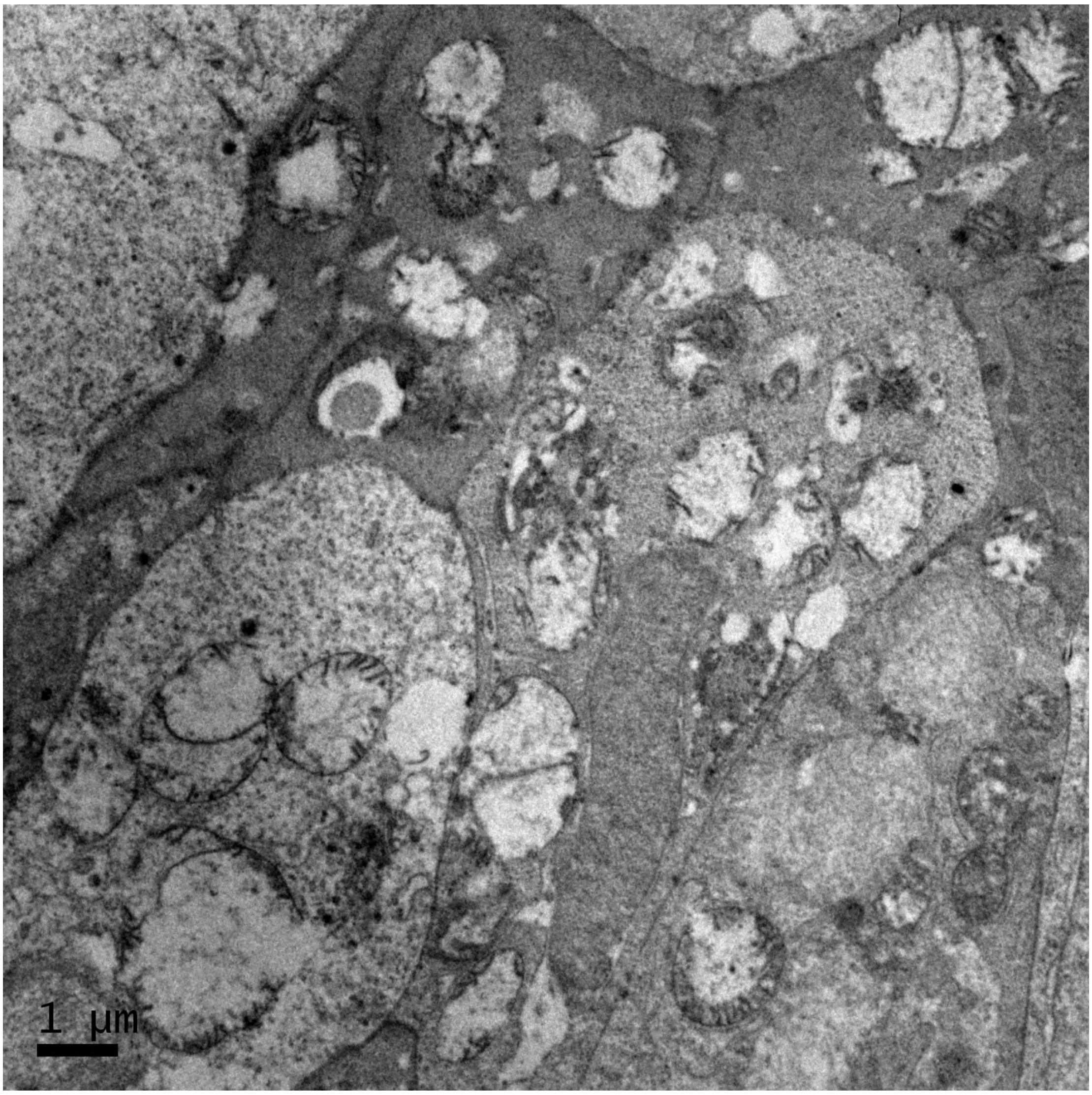

Fig.3B for the CLP+LV-NC group

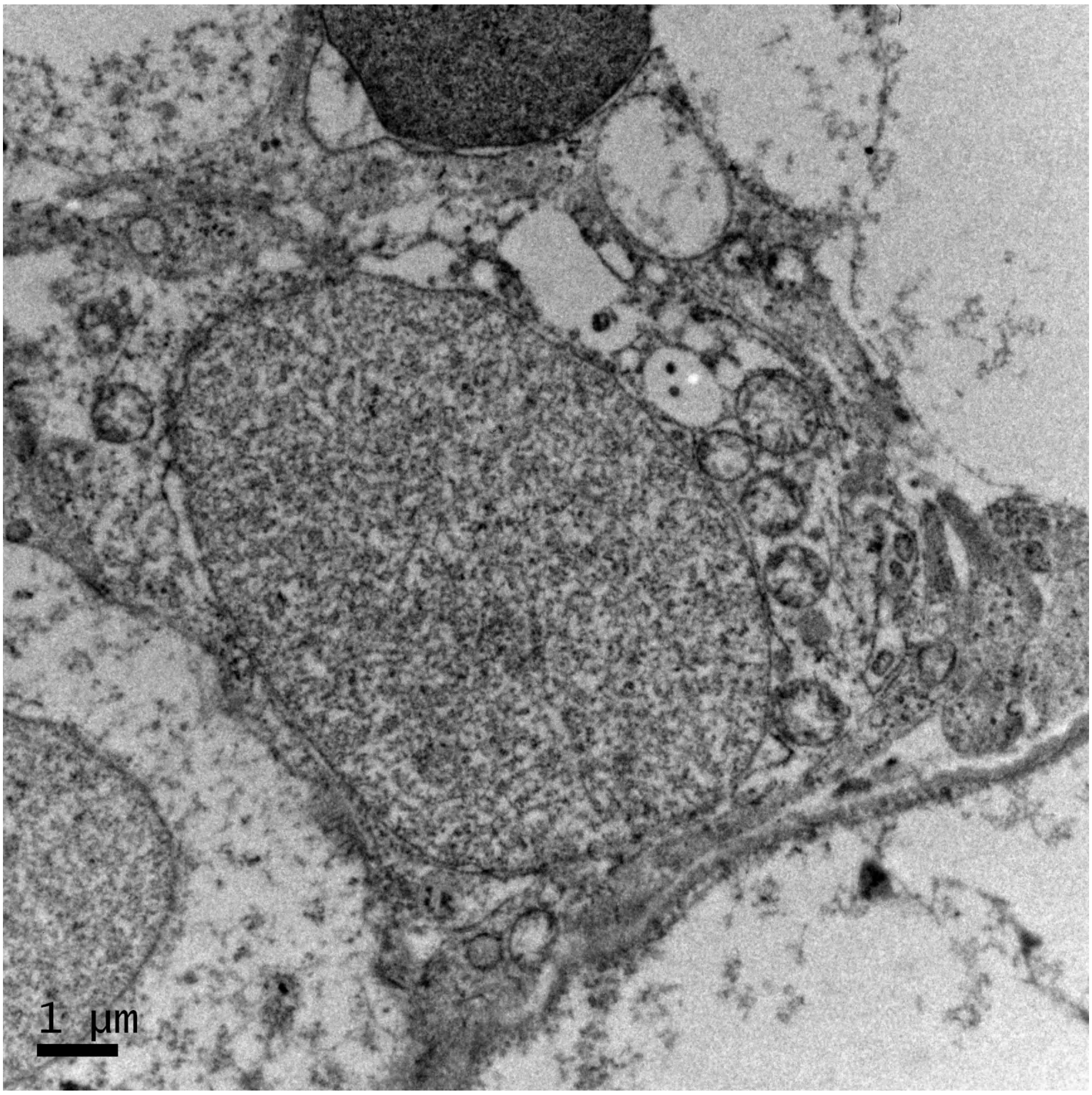
1 µm

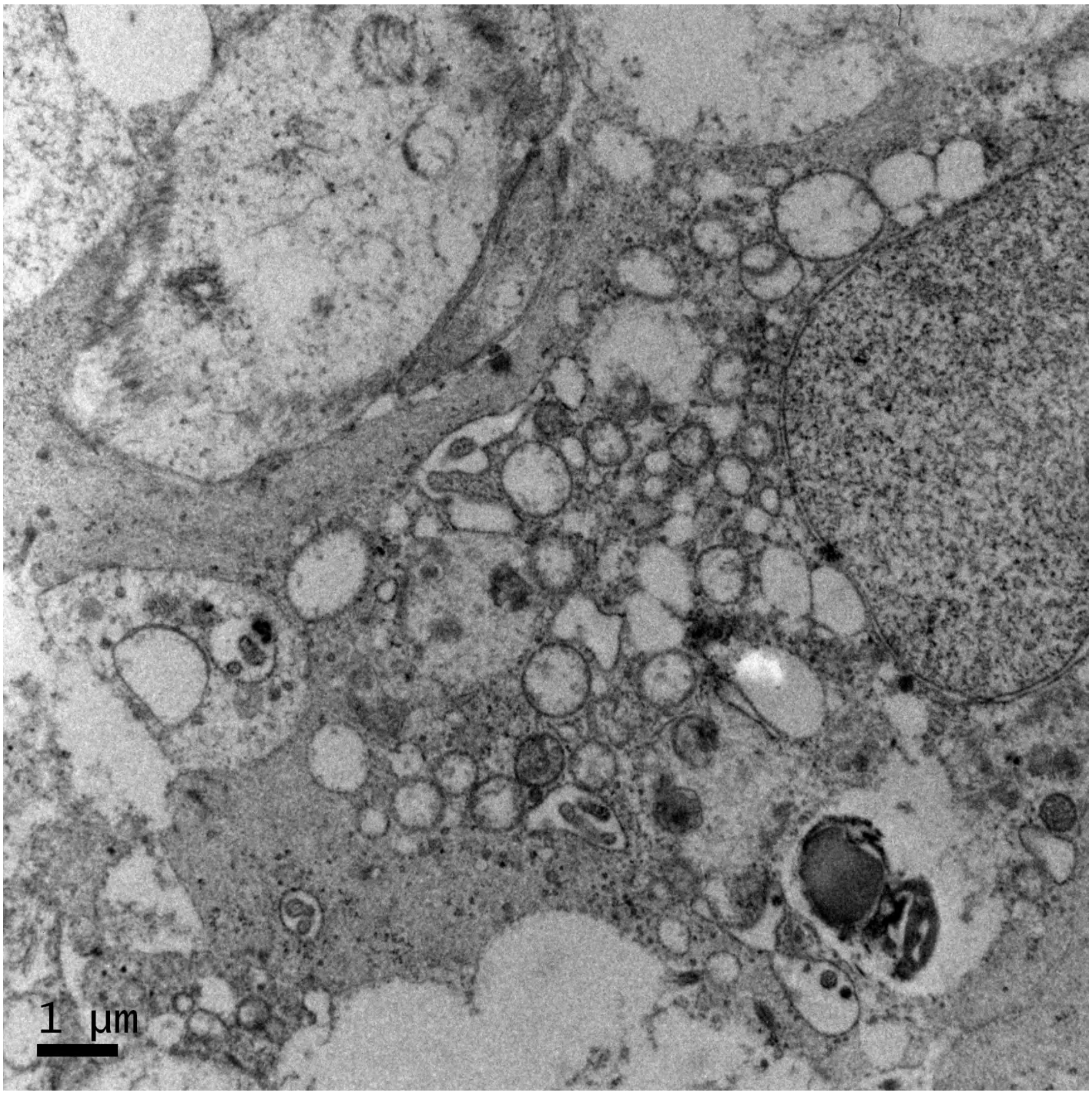
1 µm

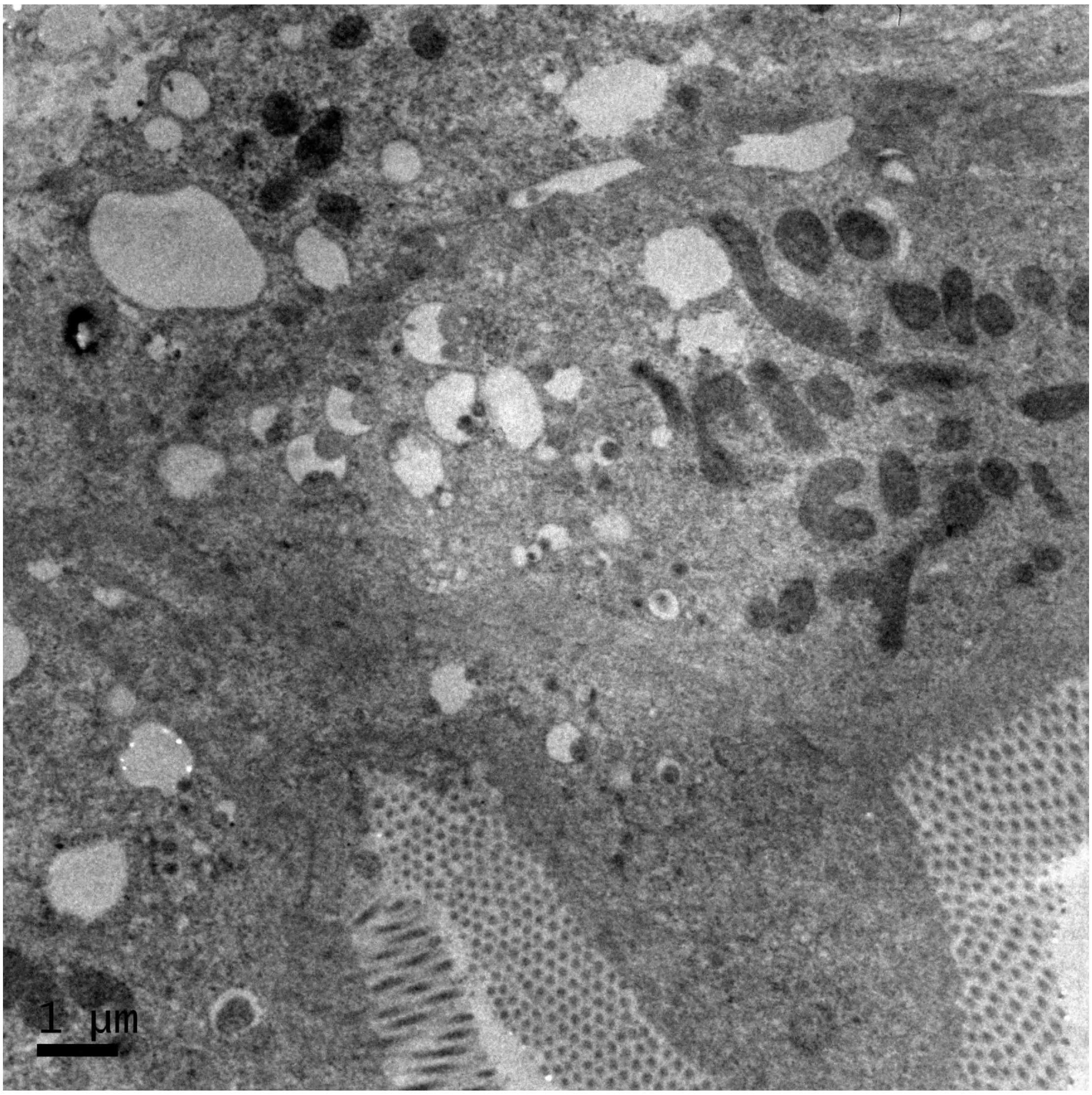

Fig.3B for the CLP+LV-miR-143 group

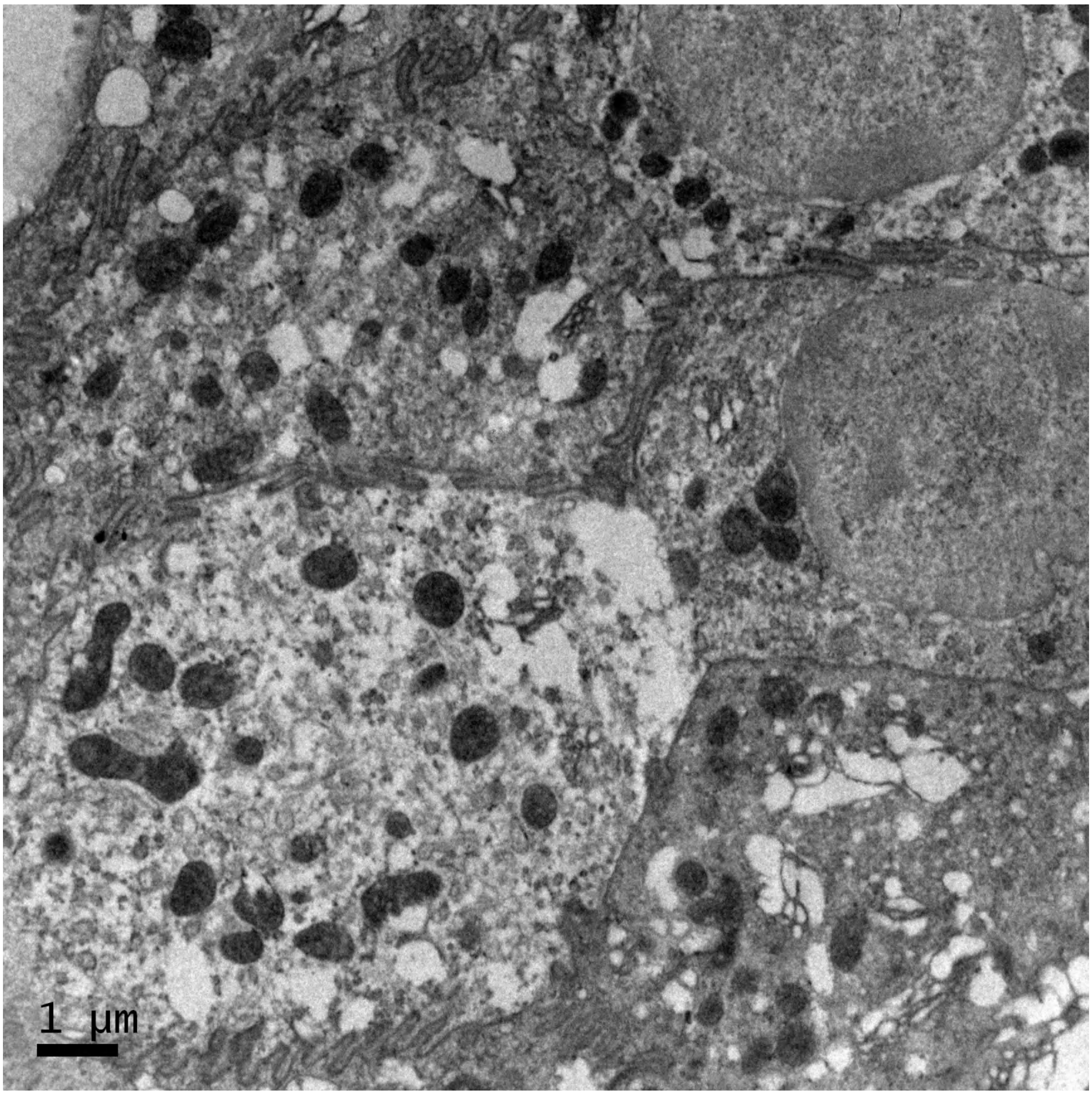
1 µm

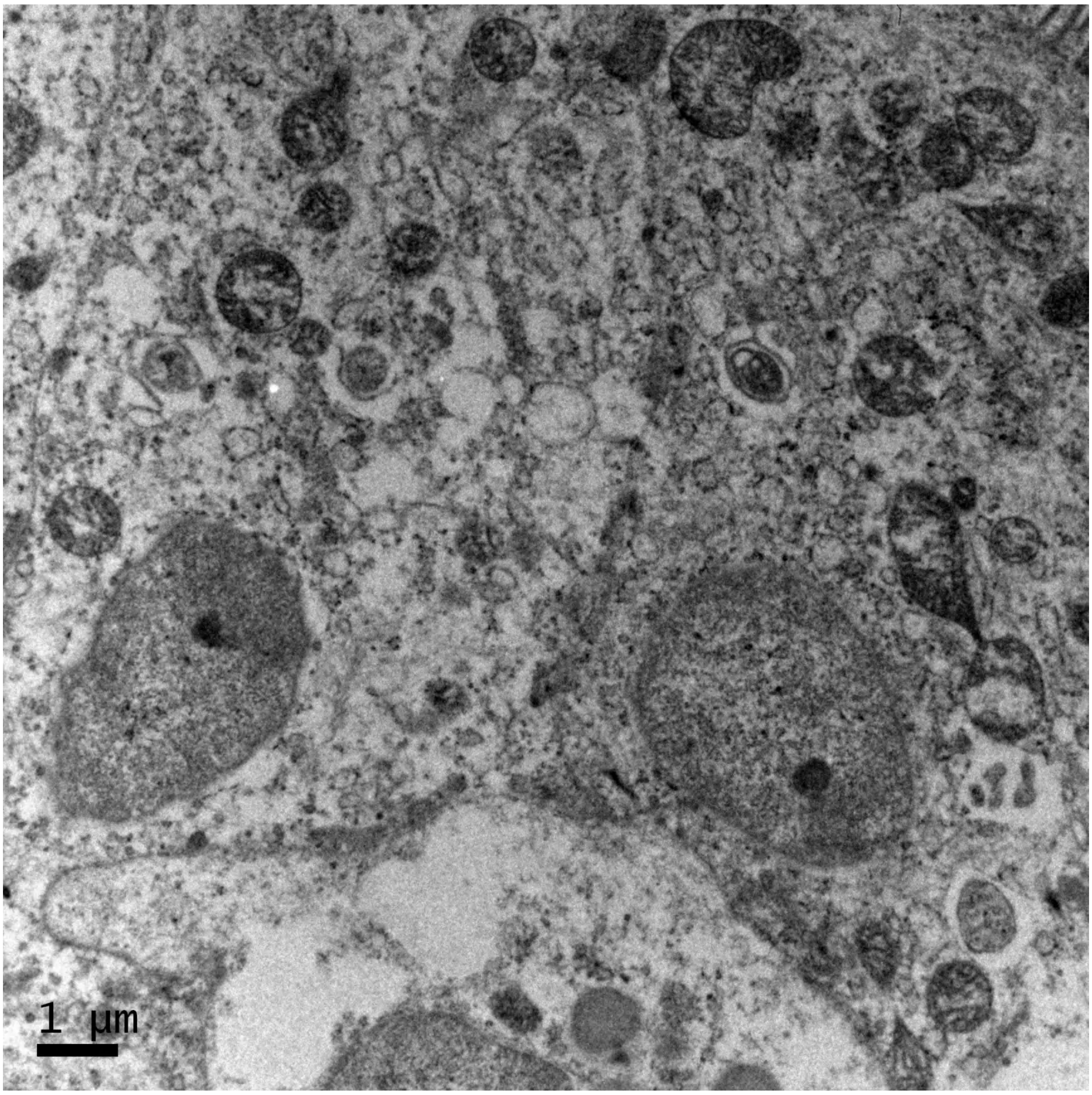
1 µm

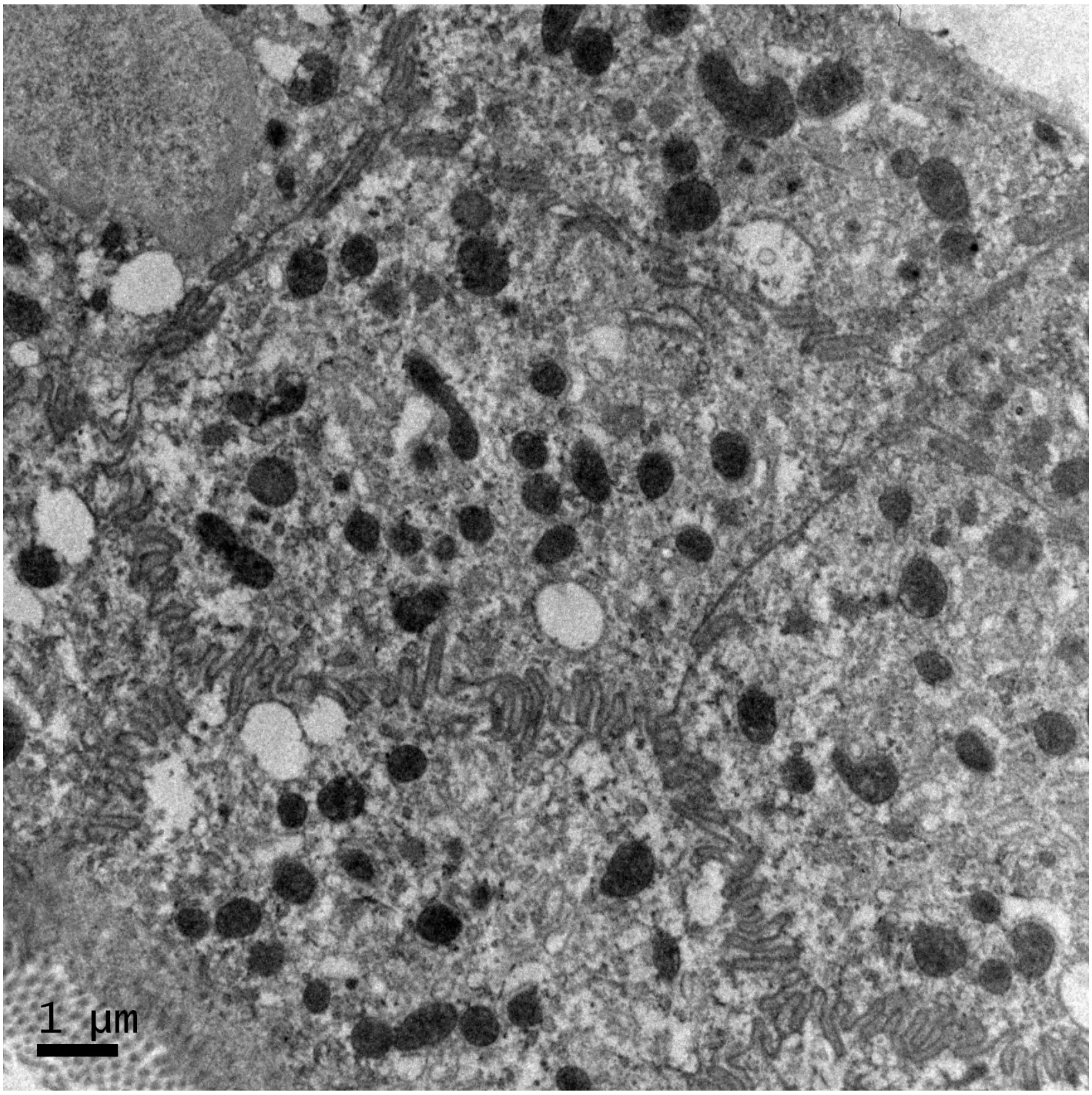

Fig.3B for the CLP+LV-NC+GHS group

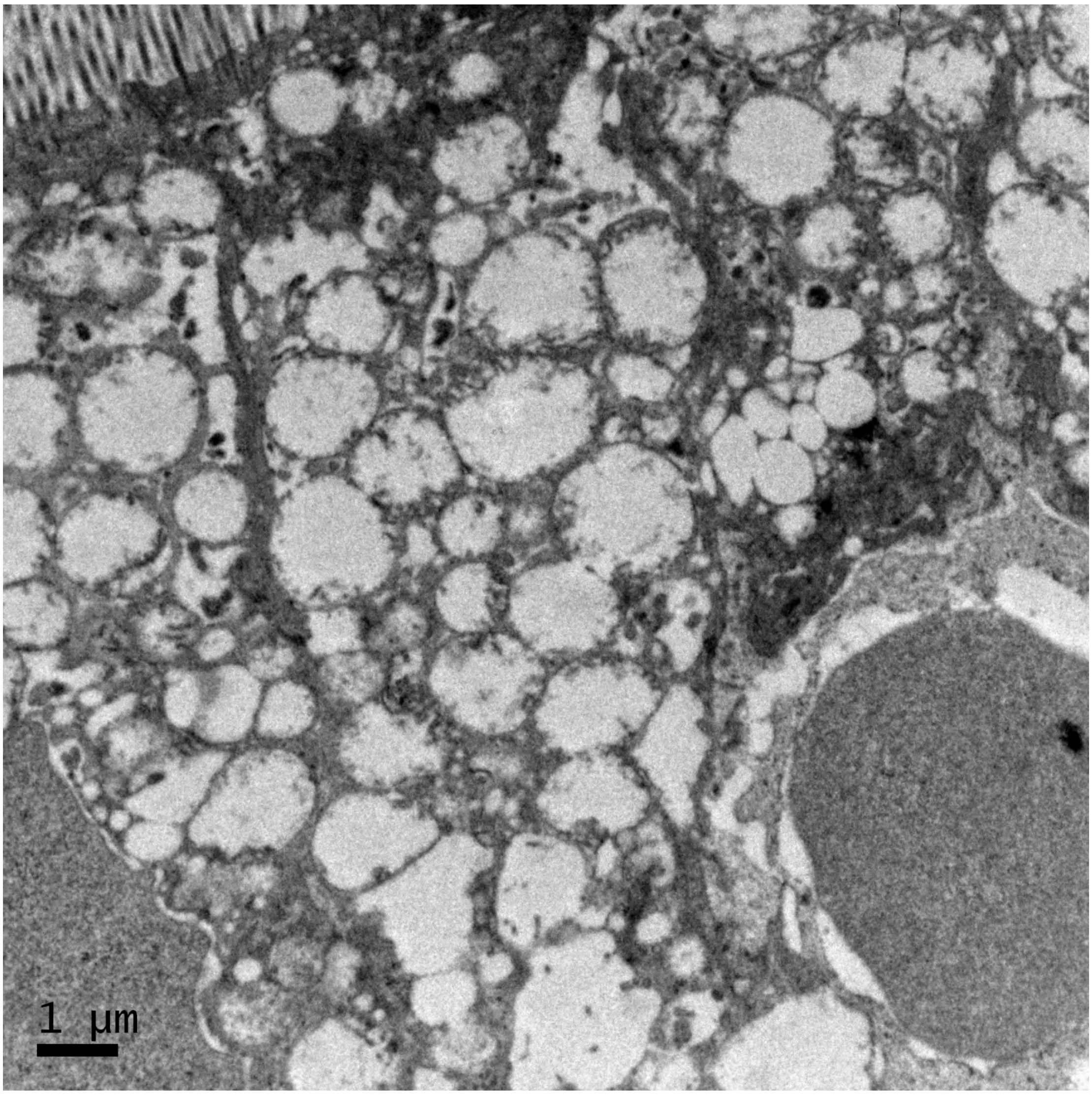
1 µm

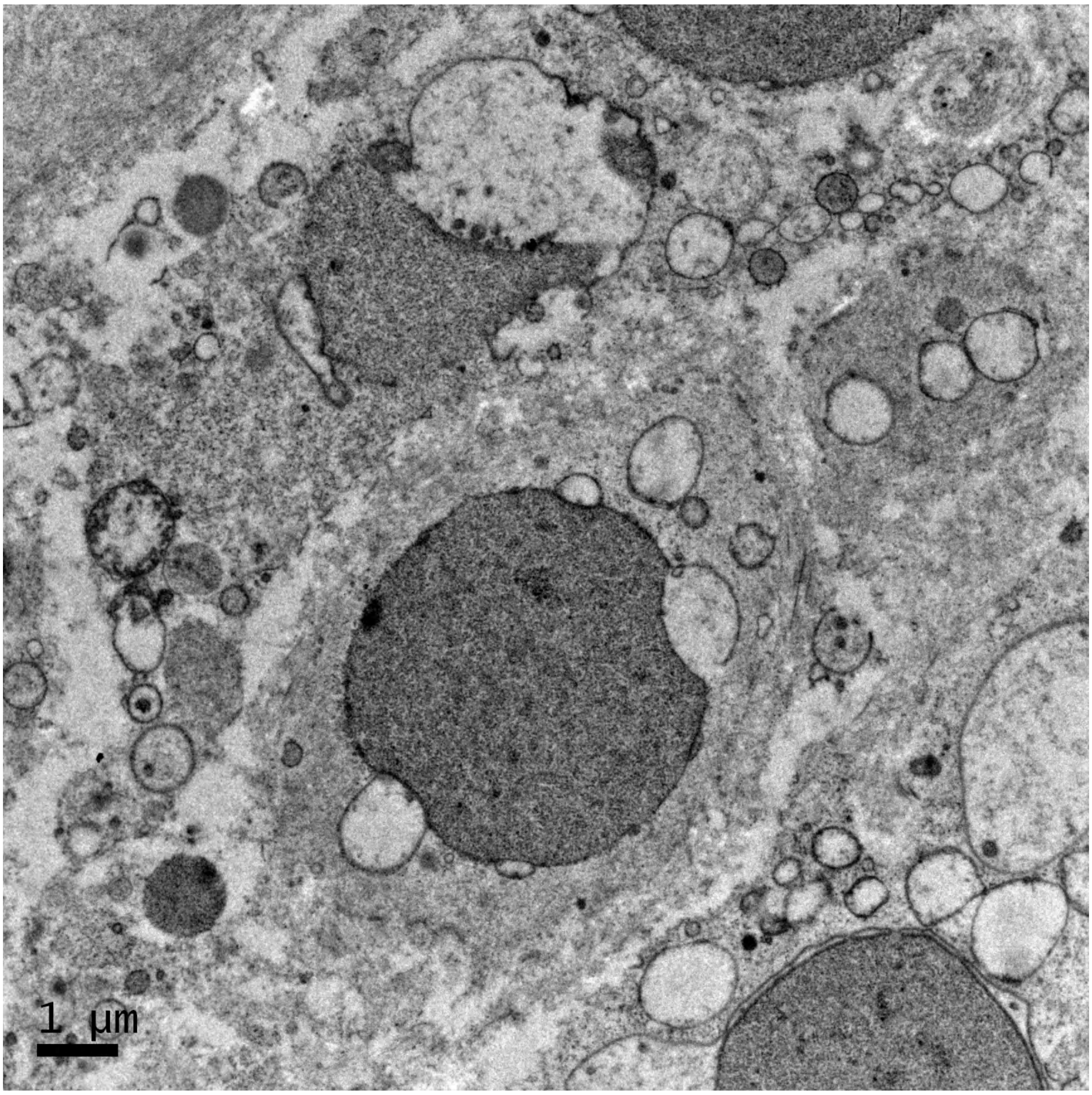

Fig.3B for the CLP+LV-miR-143+GHS group

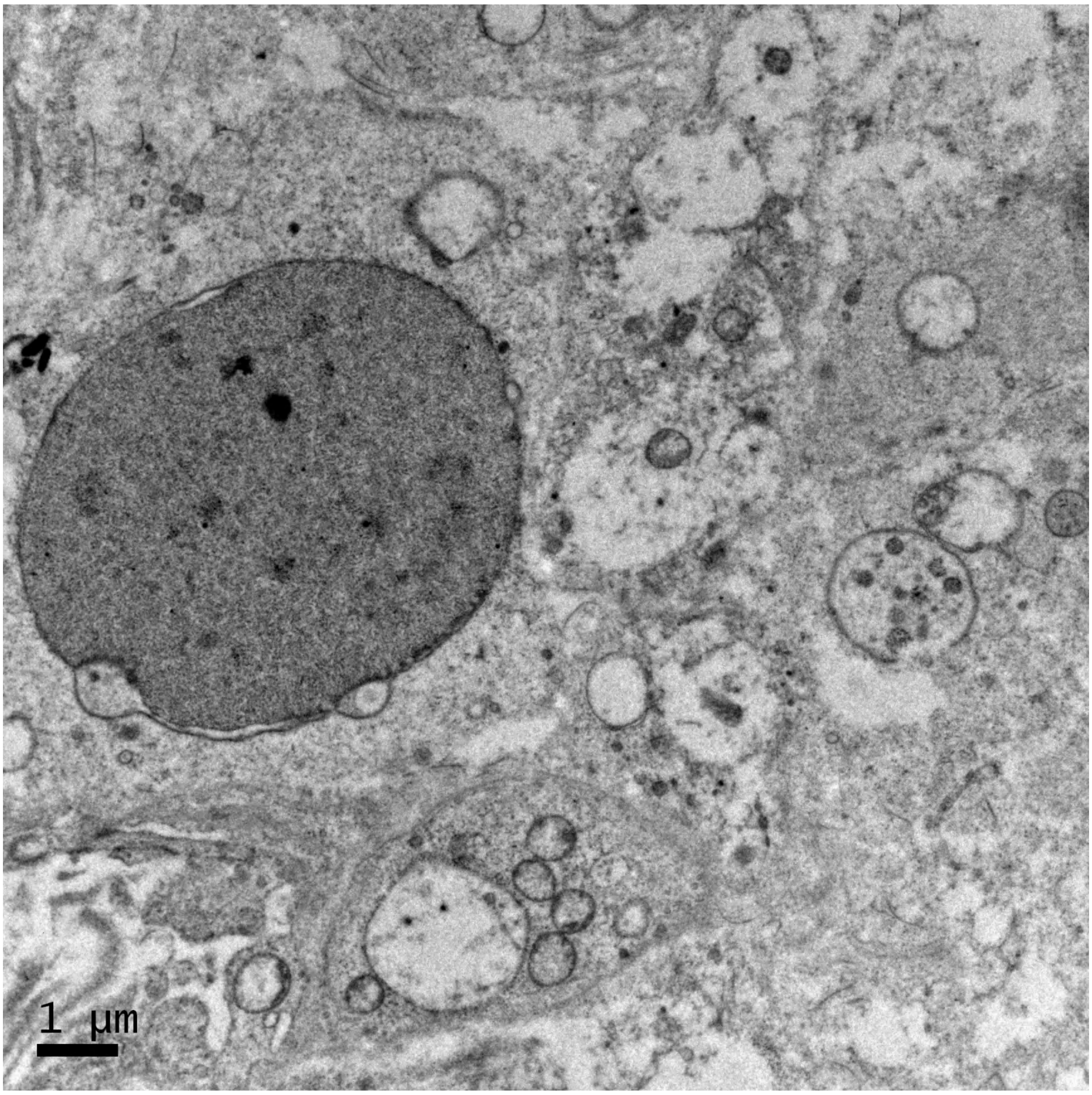
1 μm

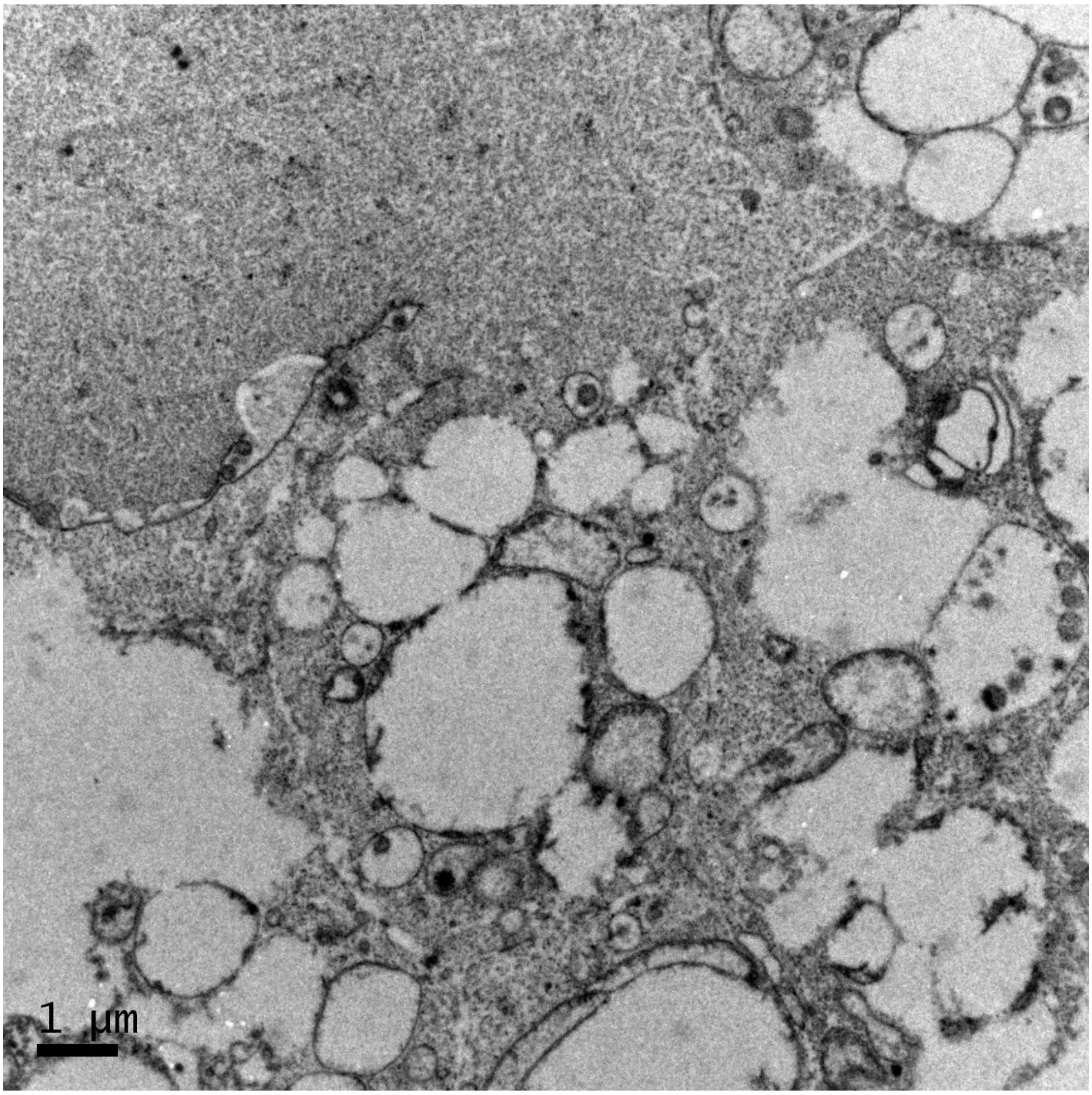
1 µm

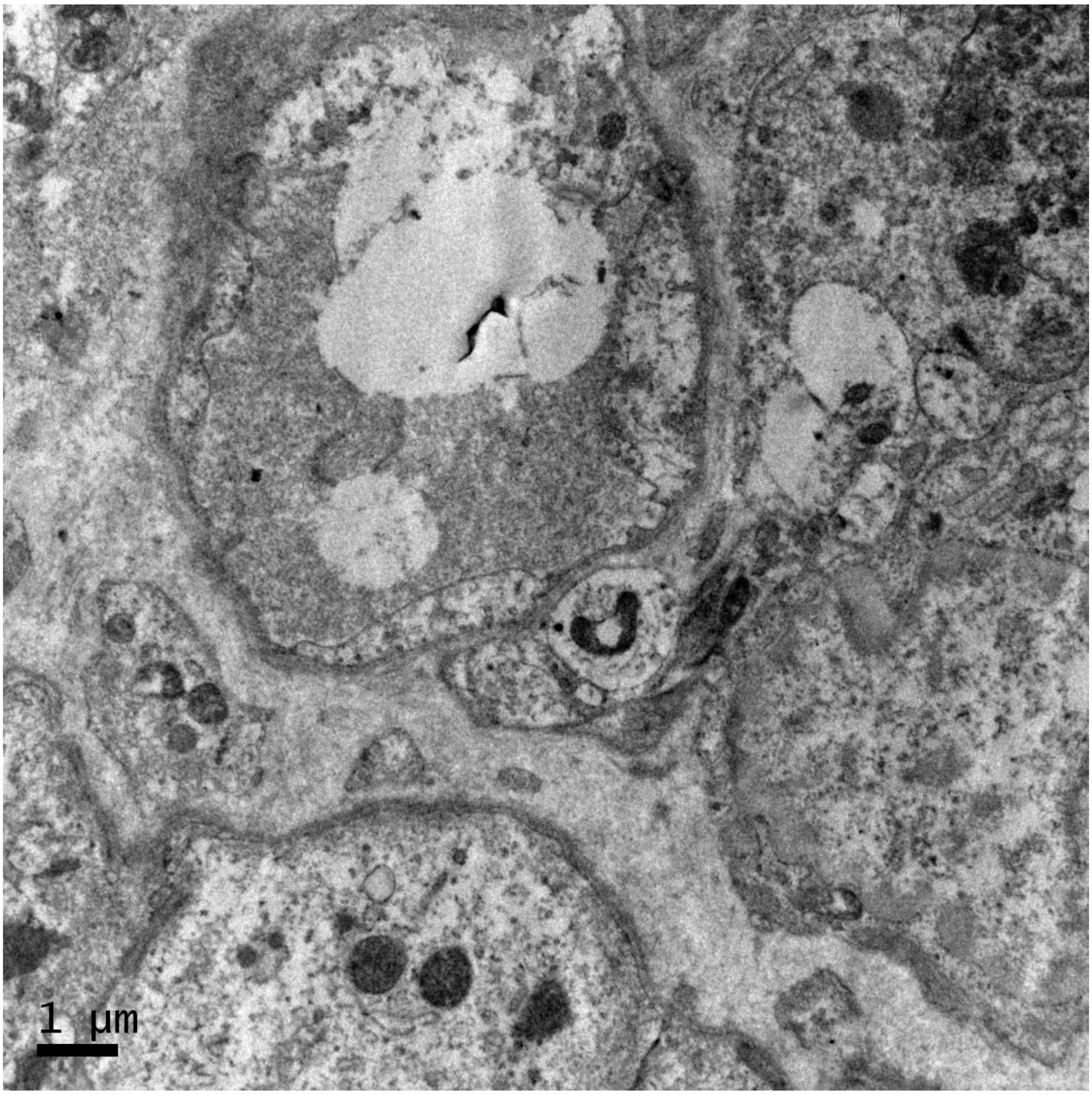

Fig.4D

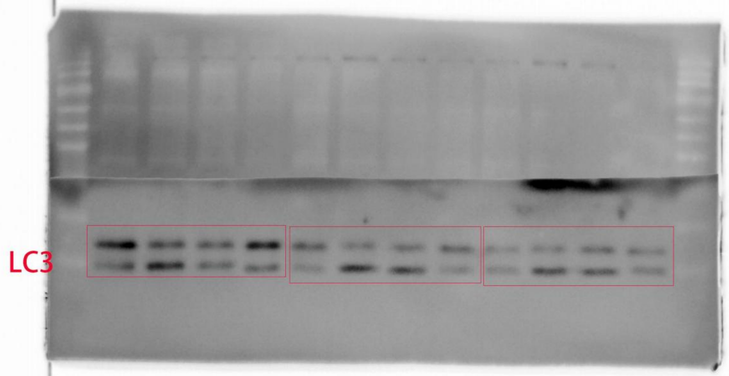
LC3

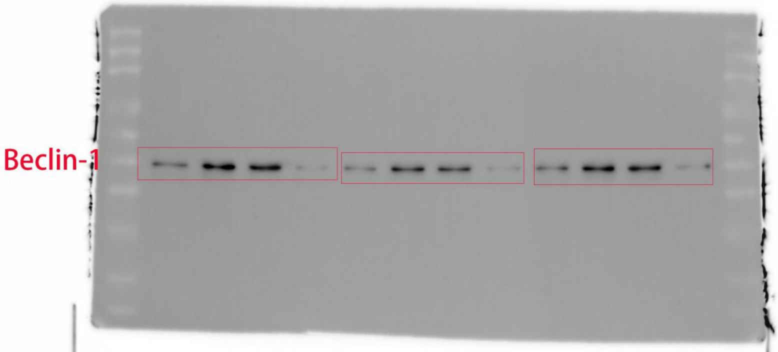
Beclin-1

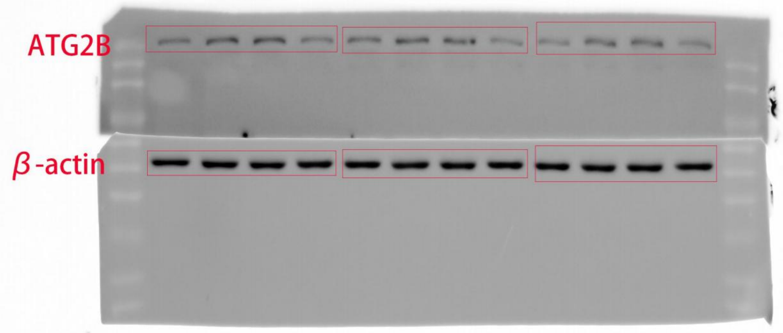
ATG2B
β-actin

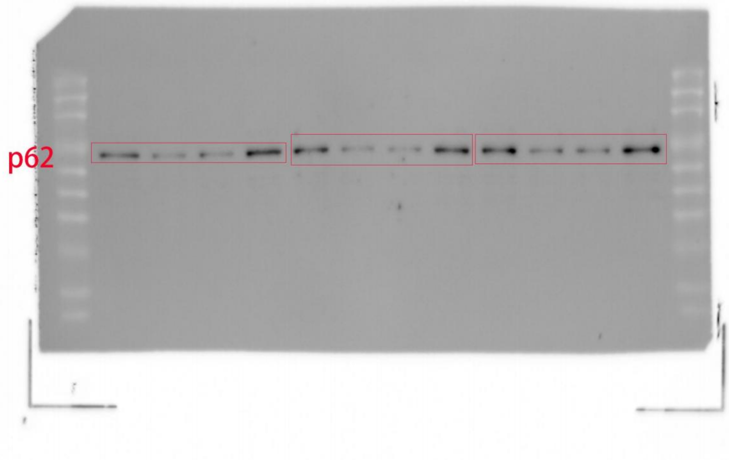
p62

Fig.4E for LPS group

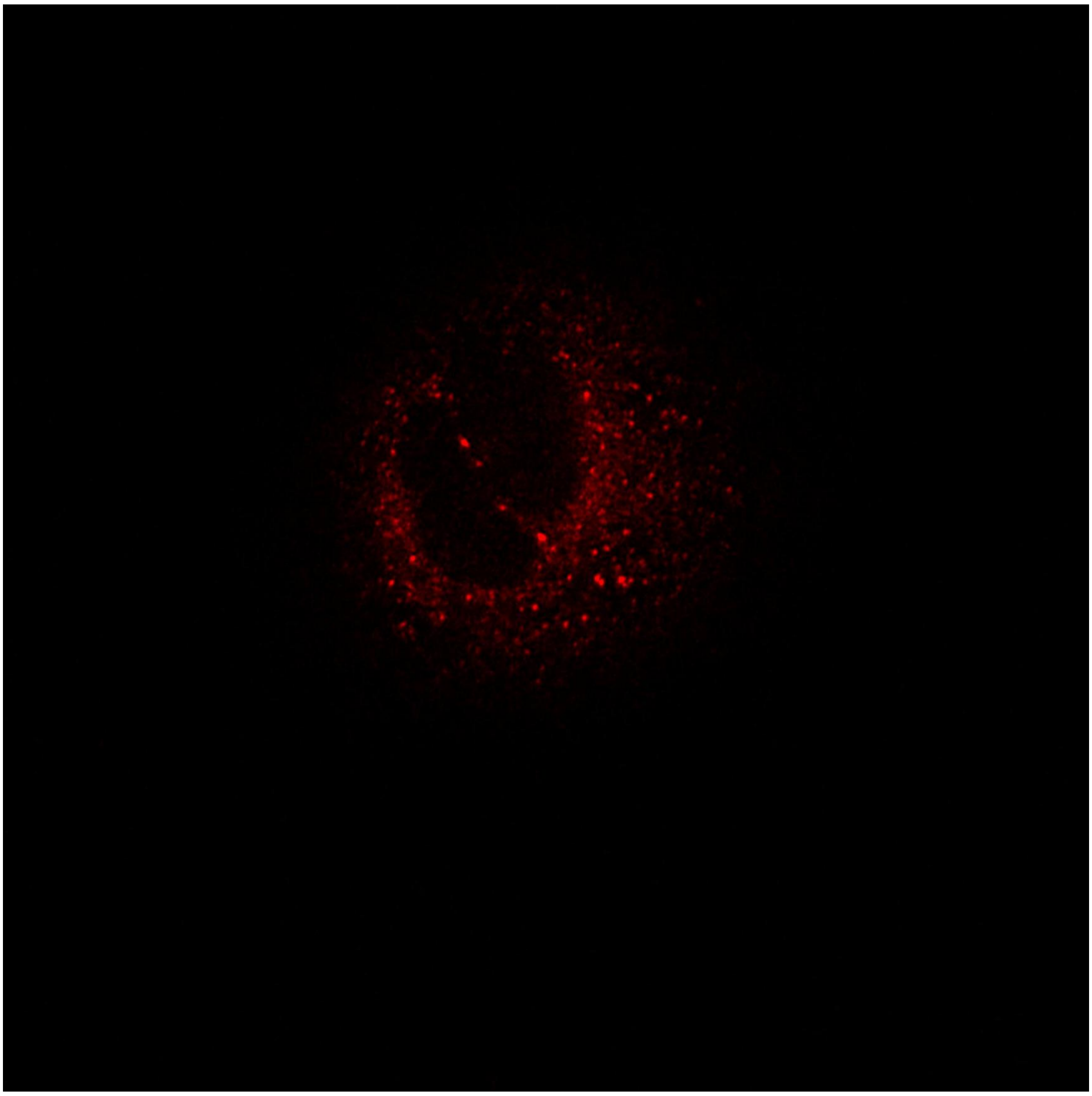

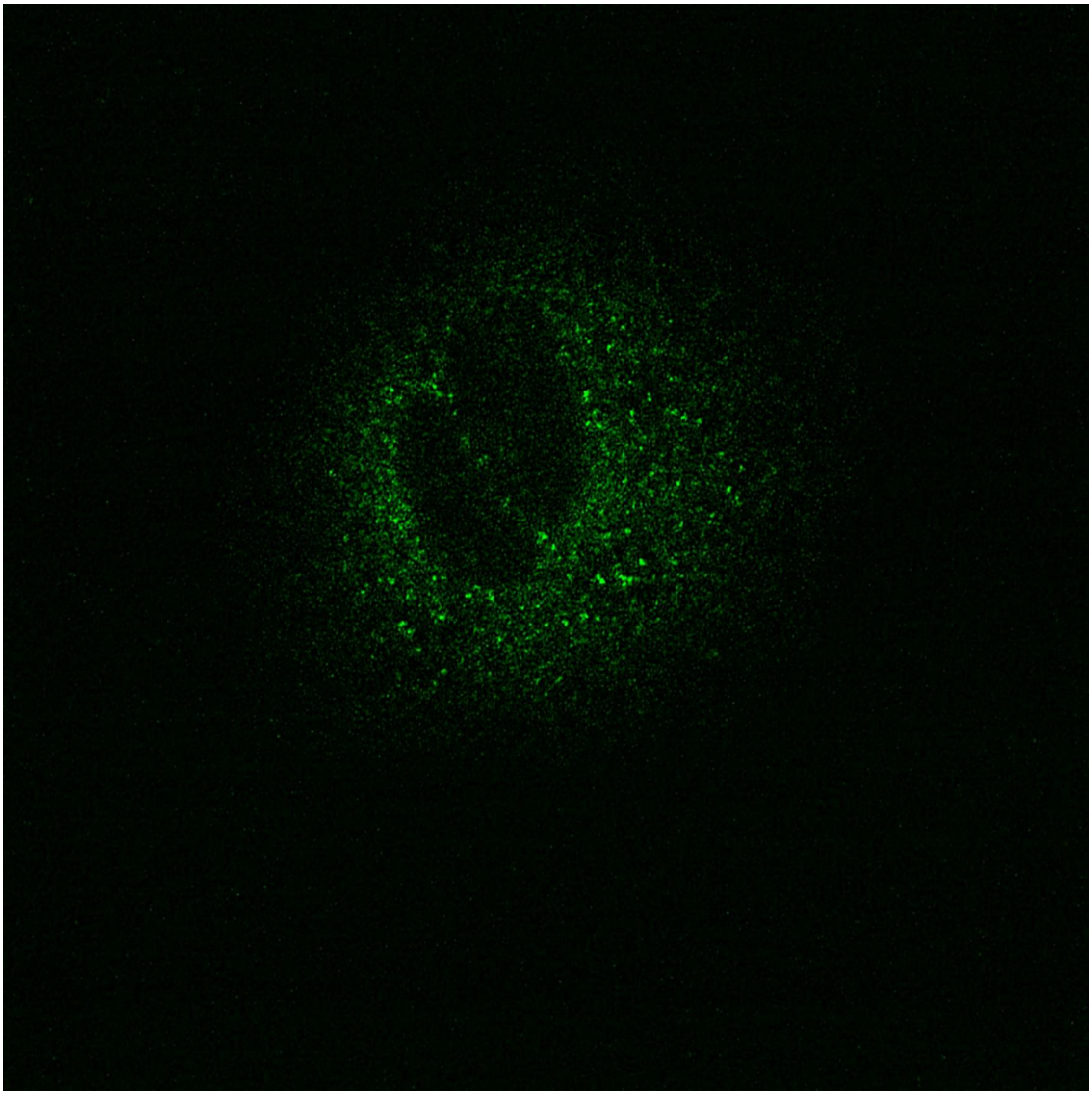

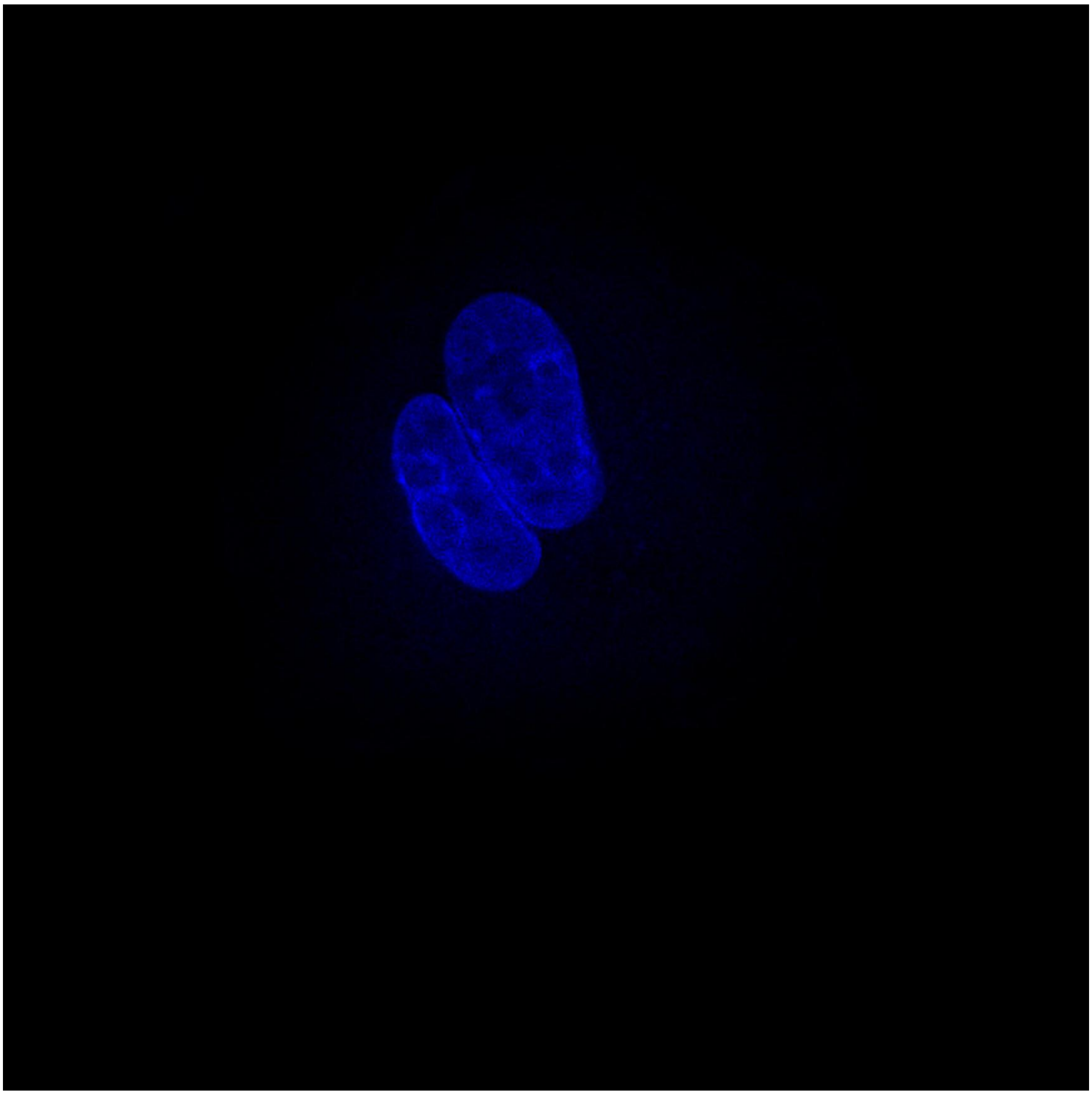

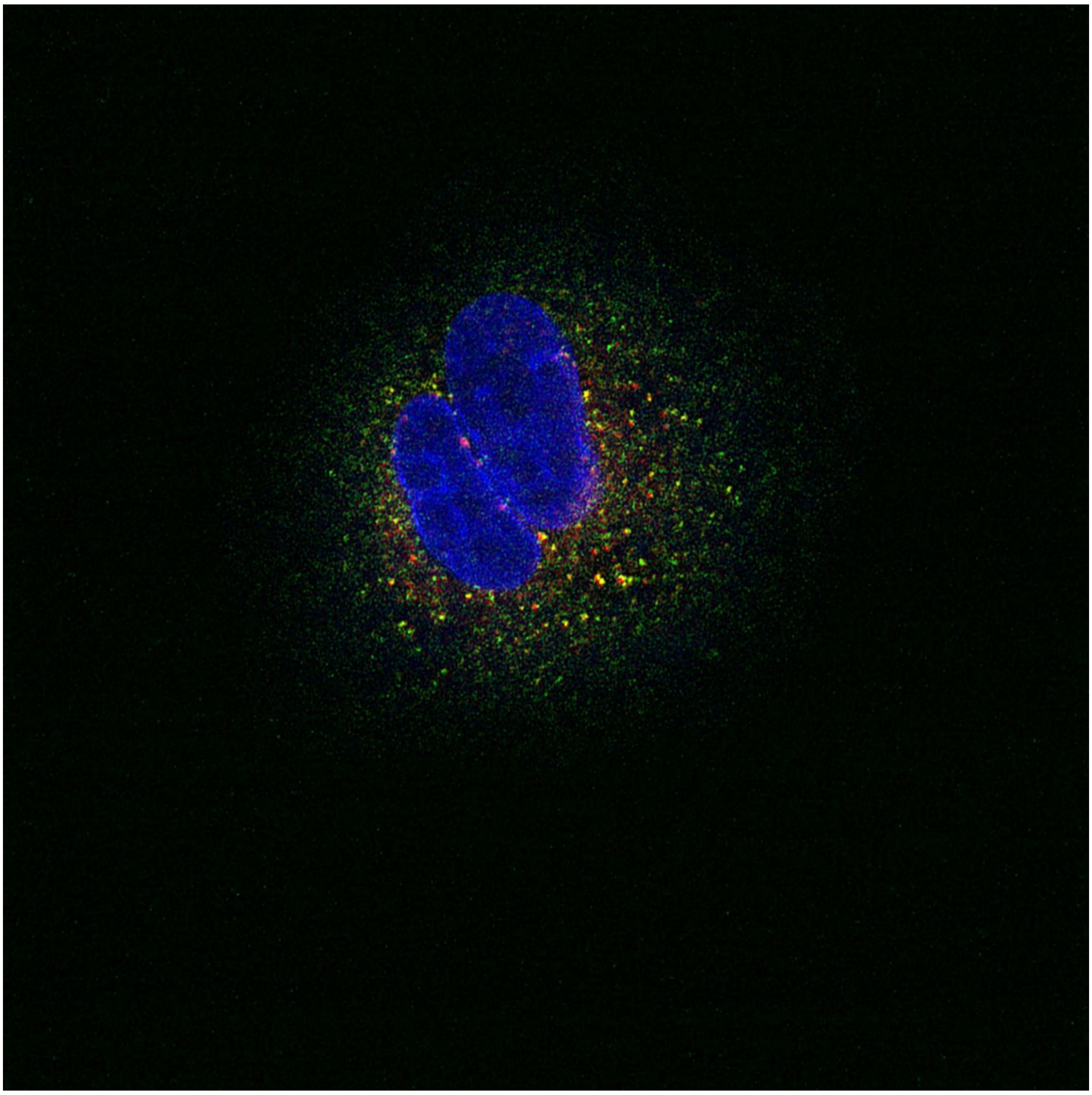

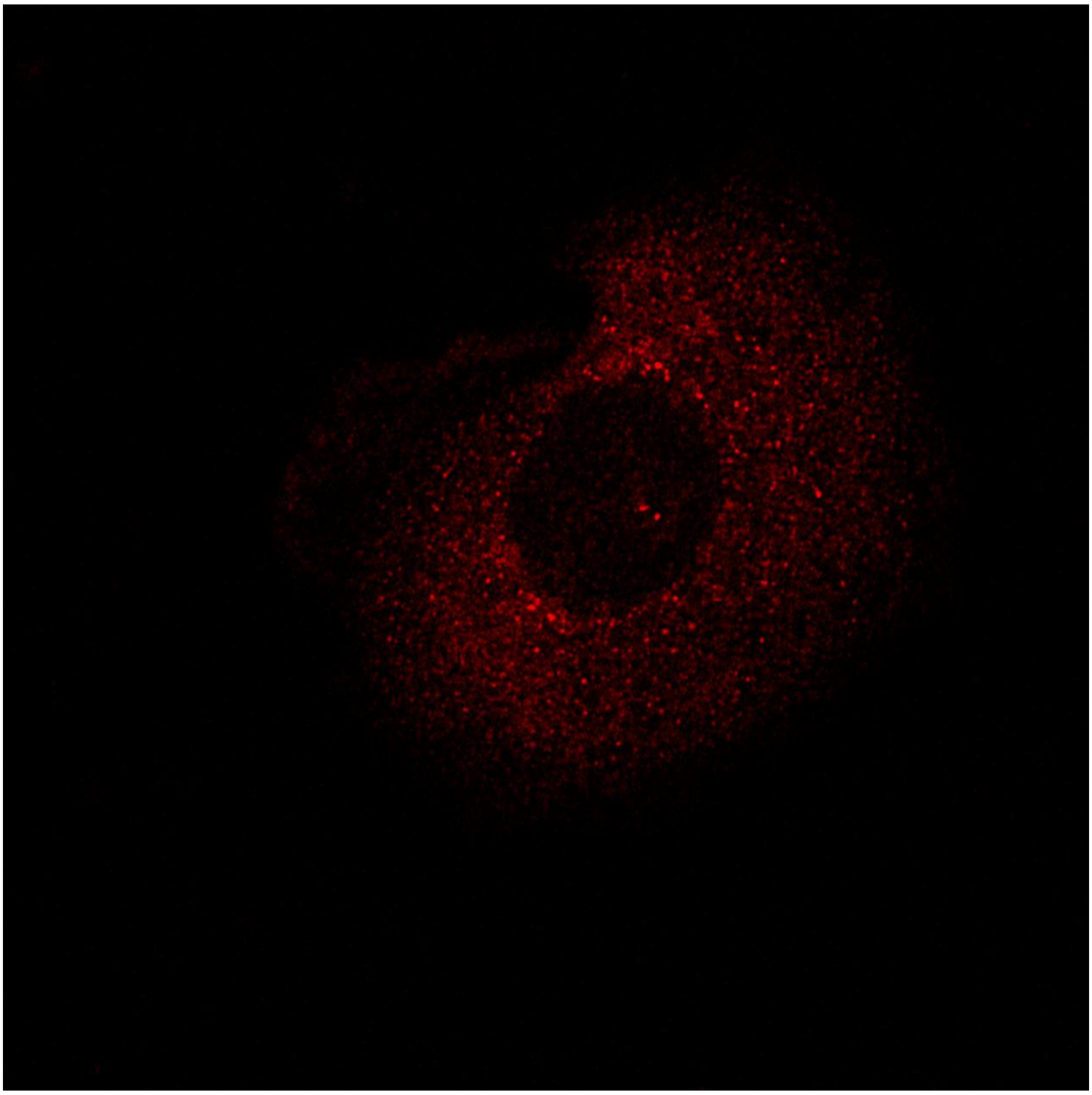

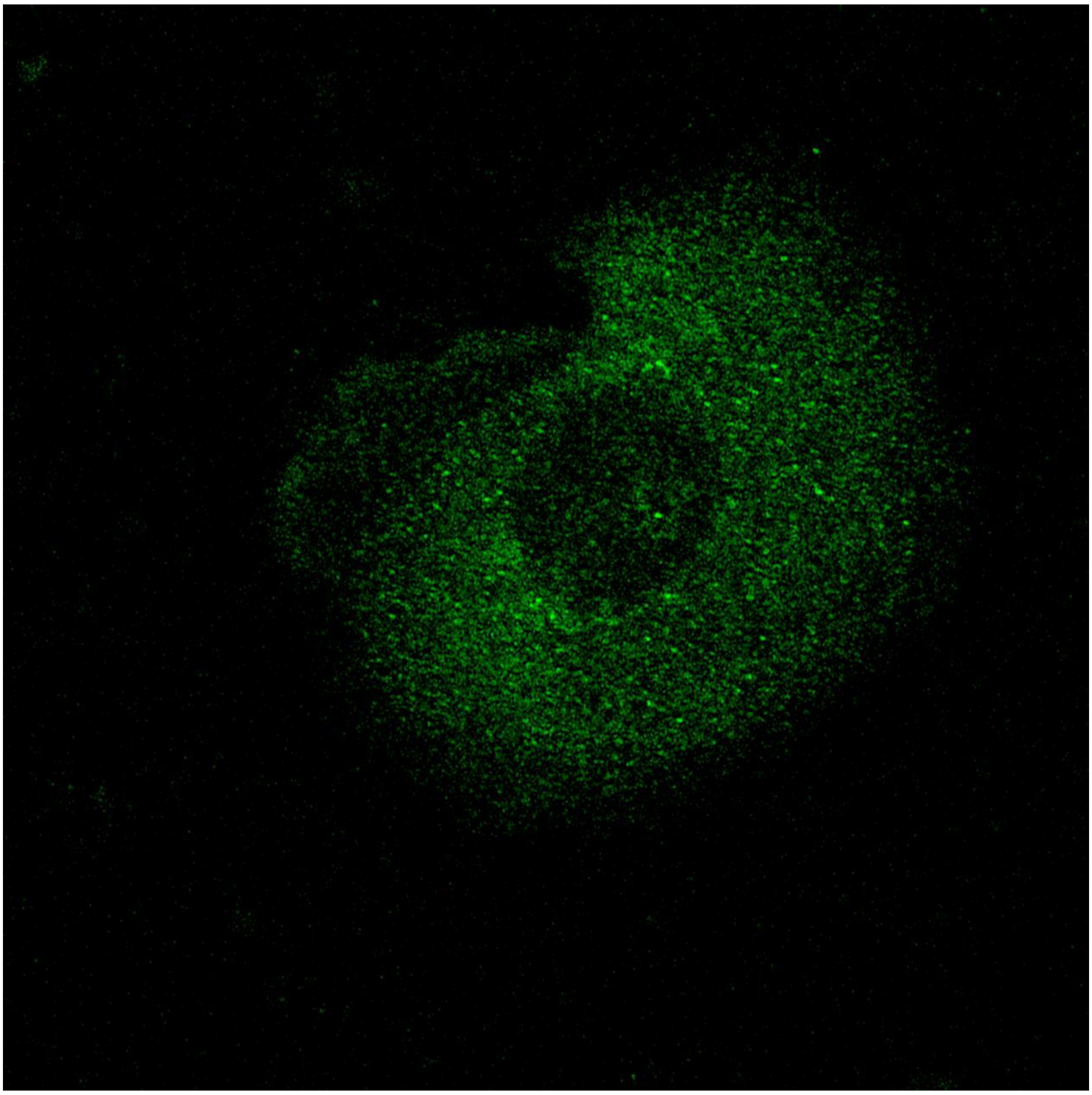

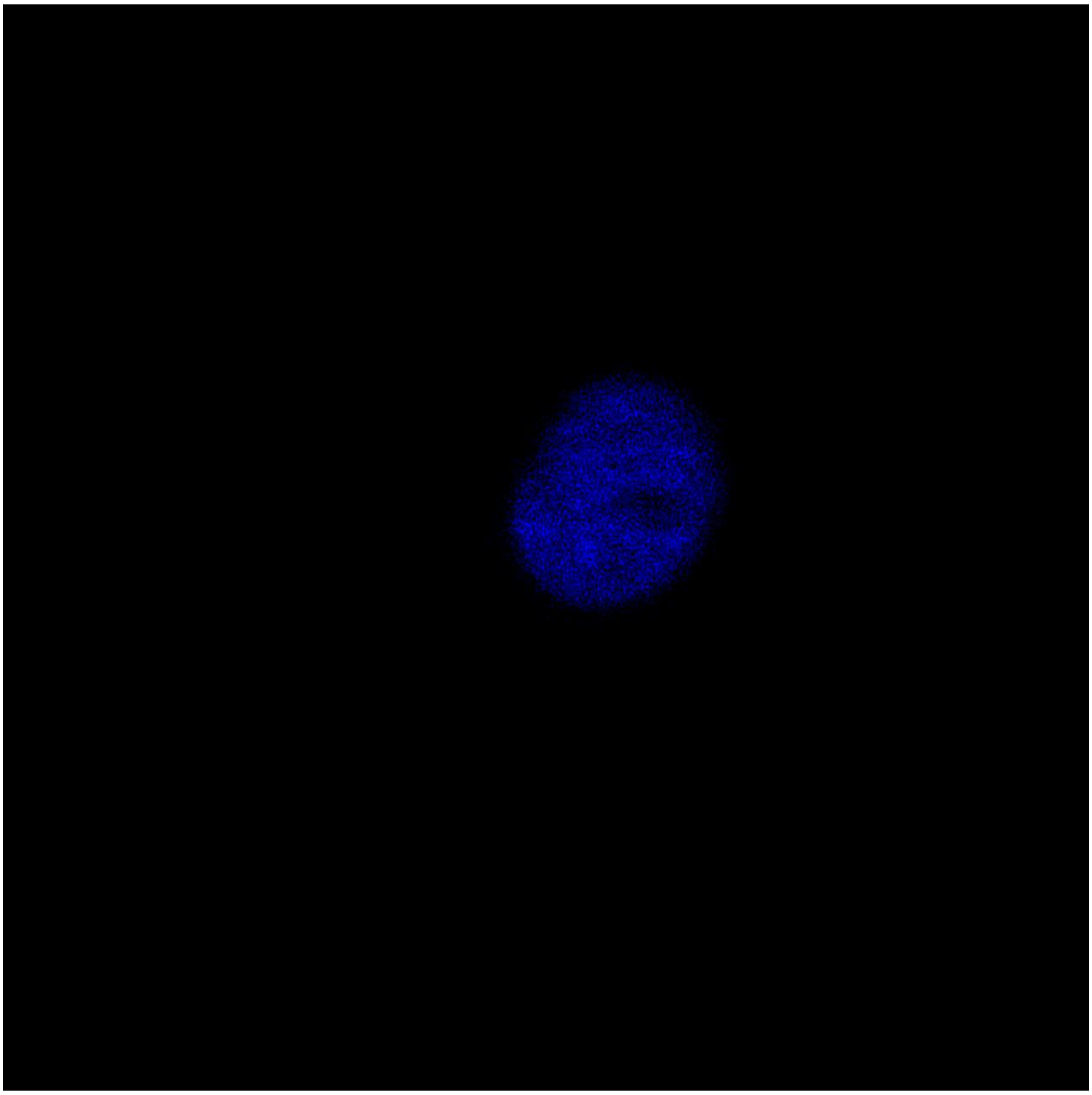

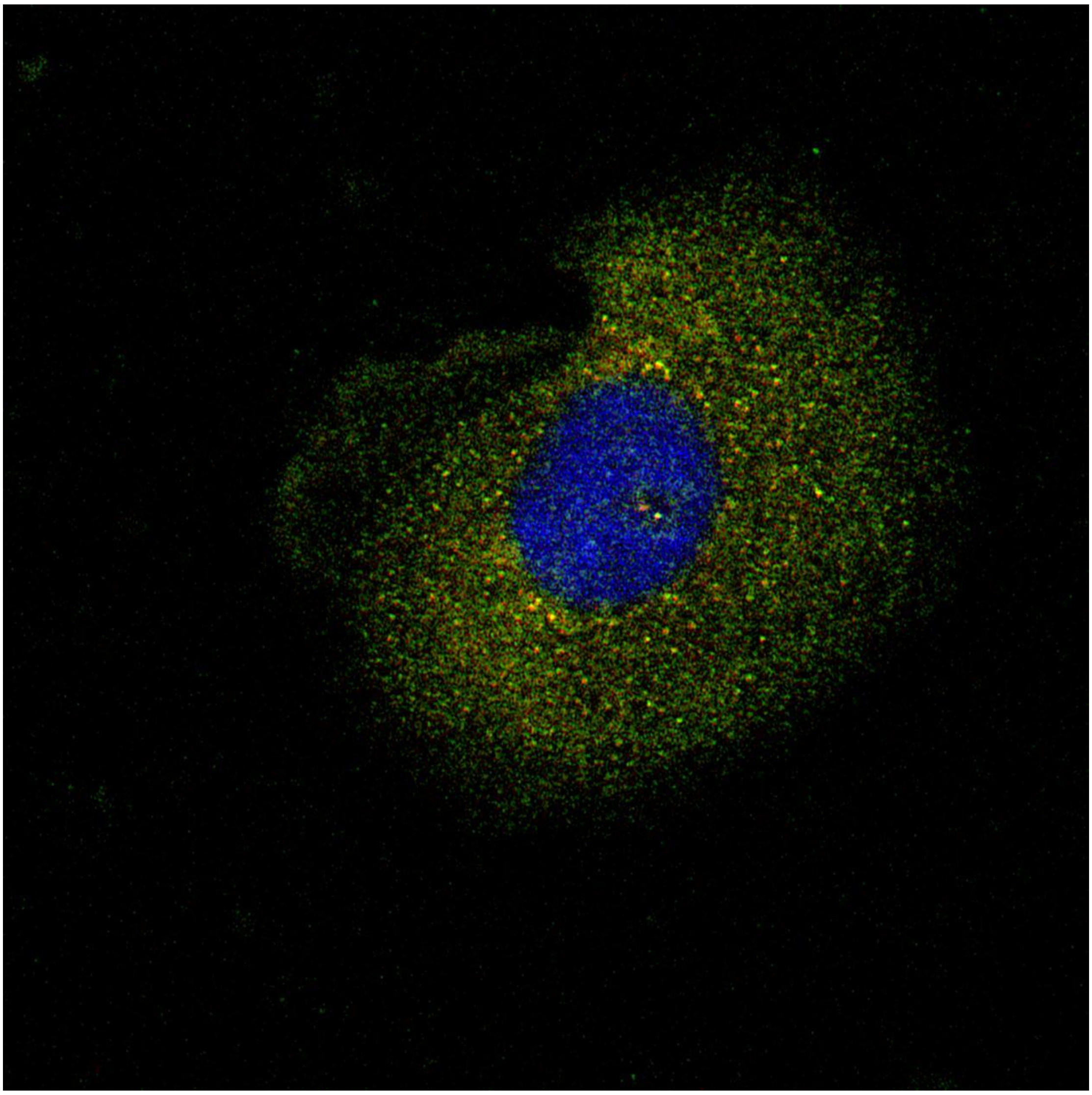

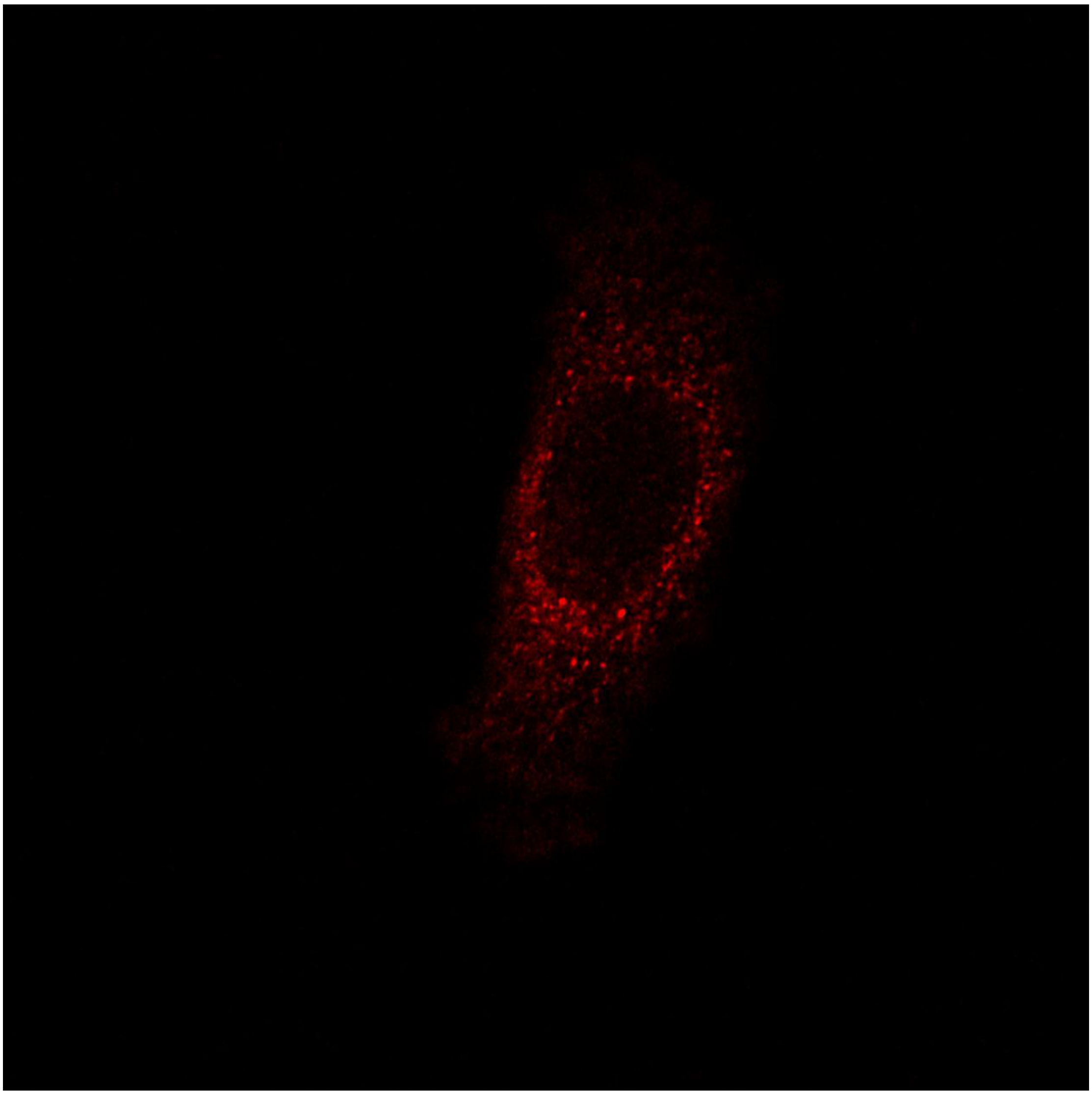

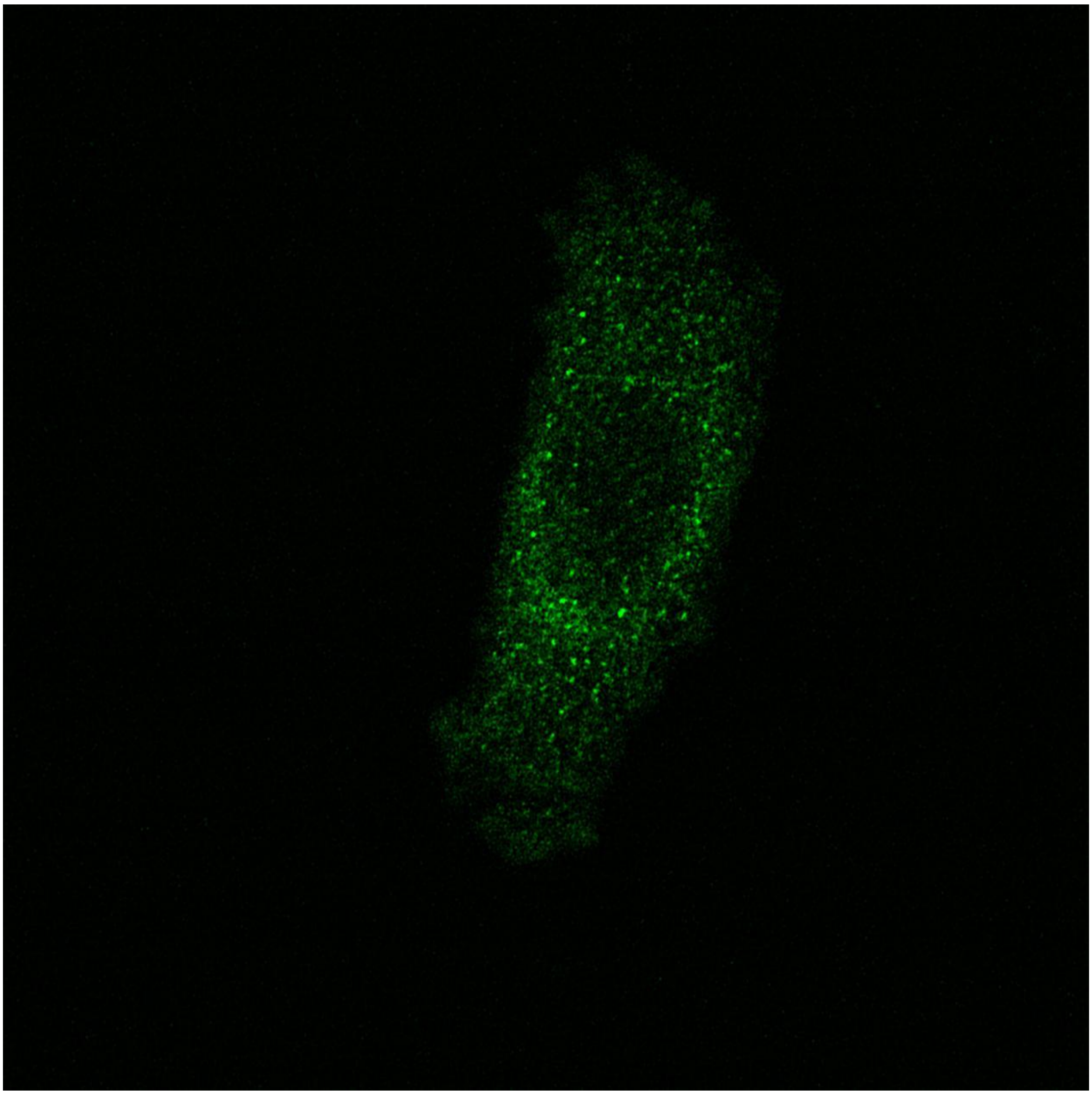

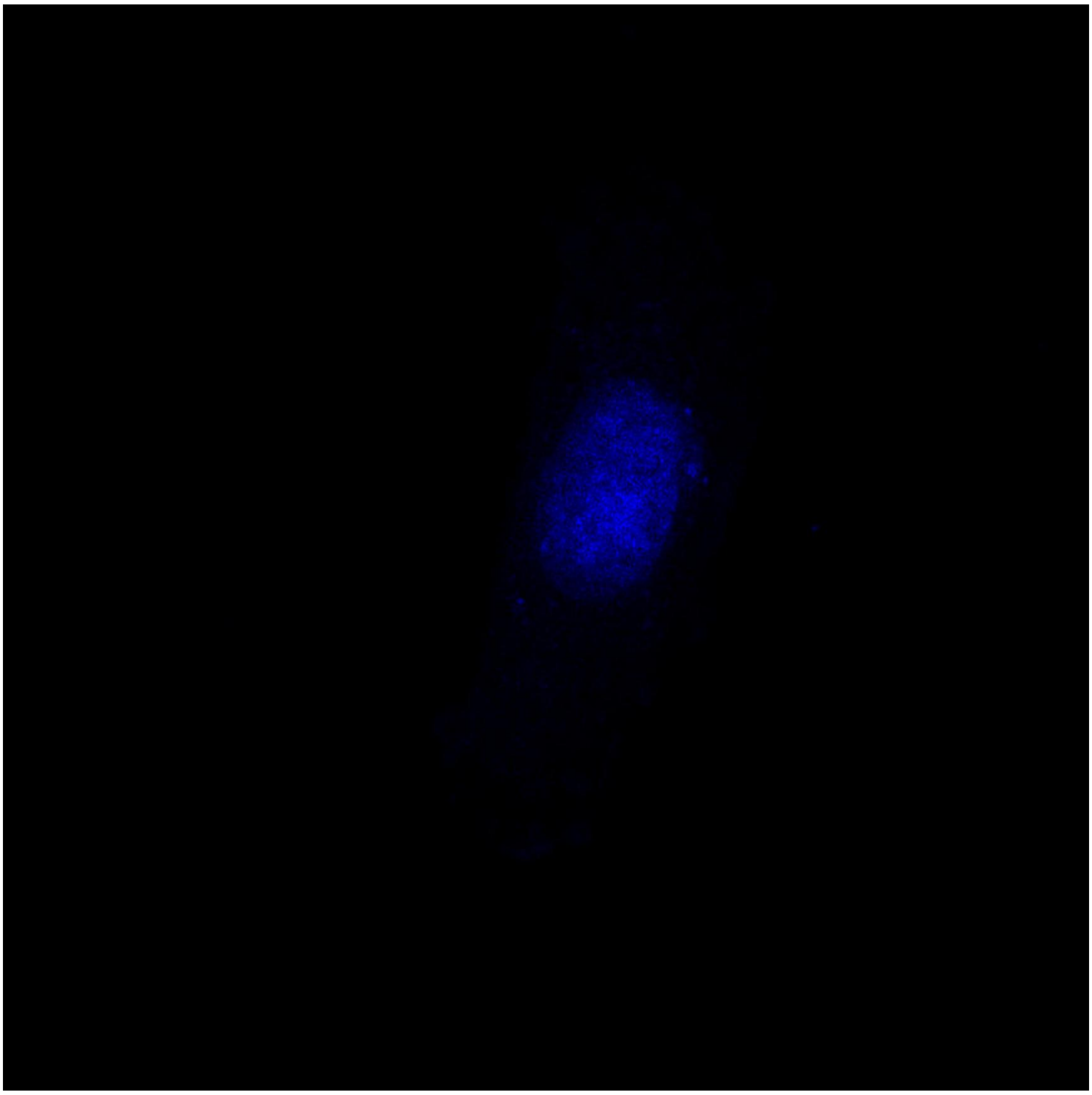

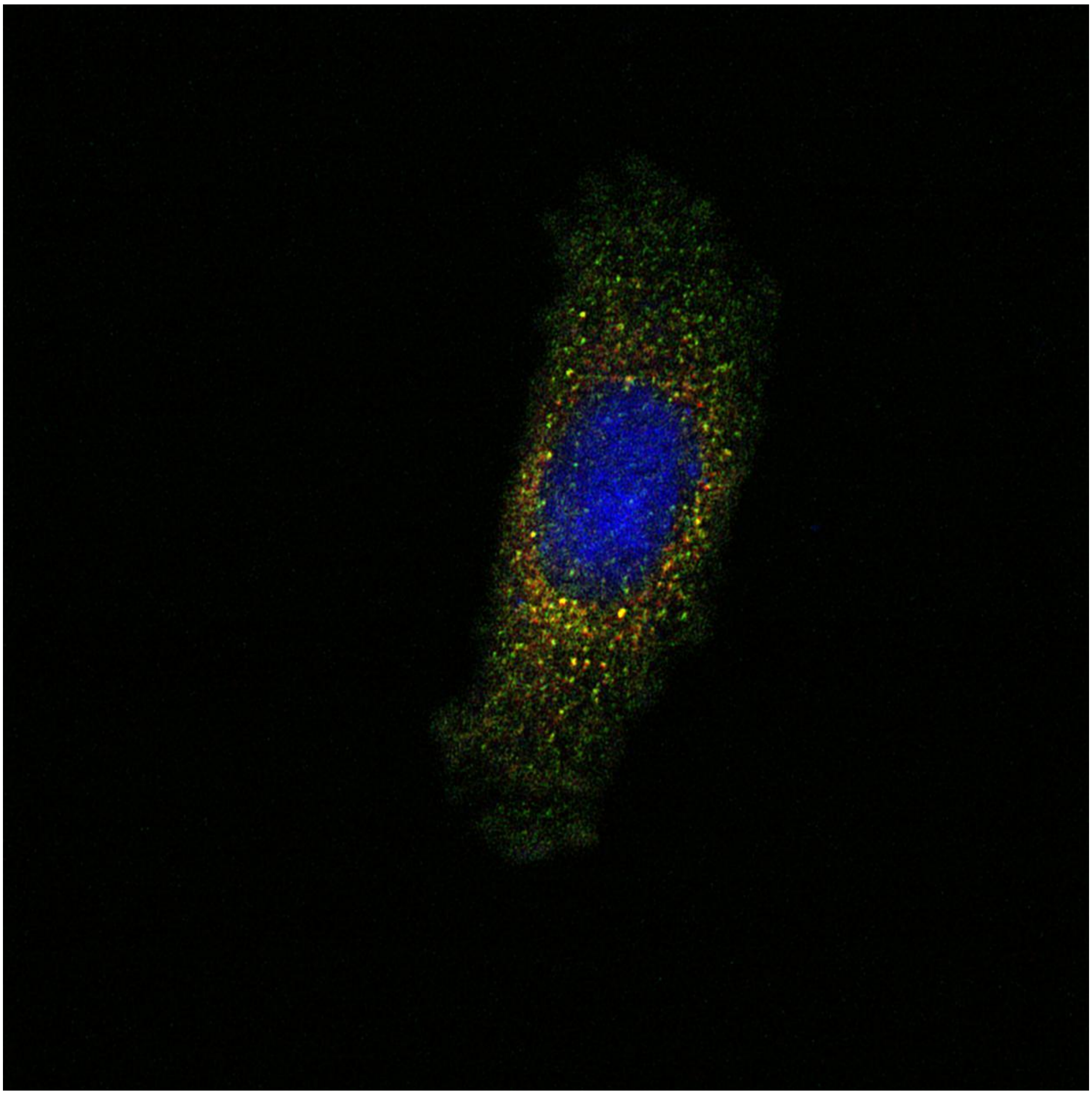

Fig.4E for LPS+GHS group

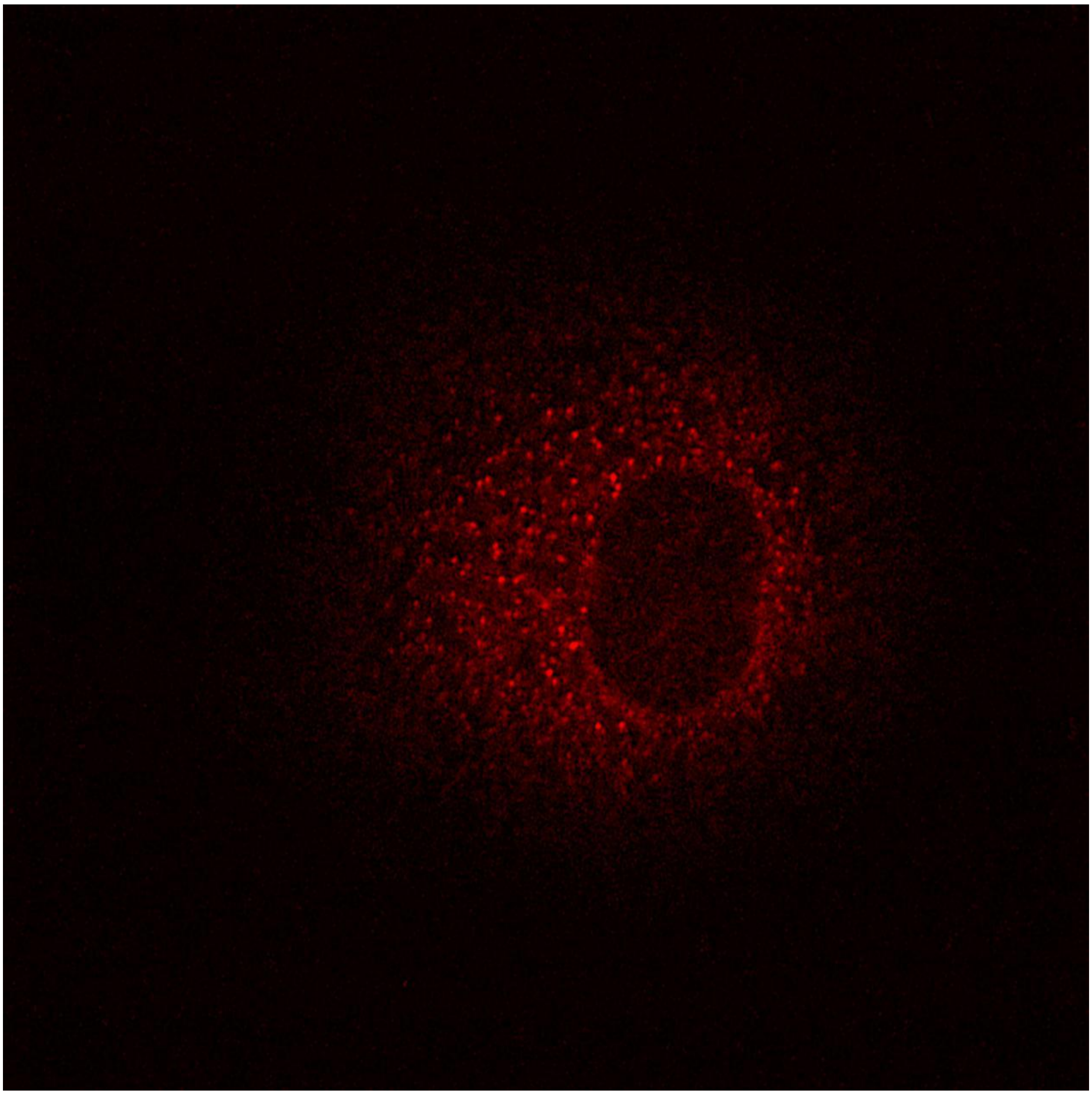

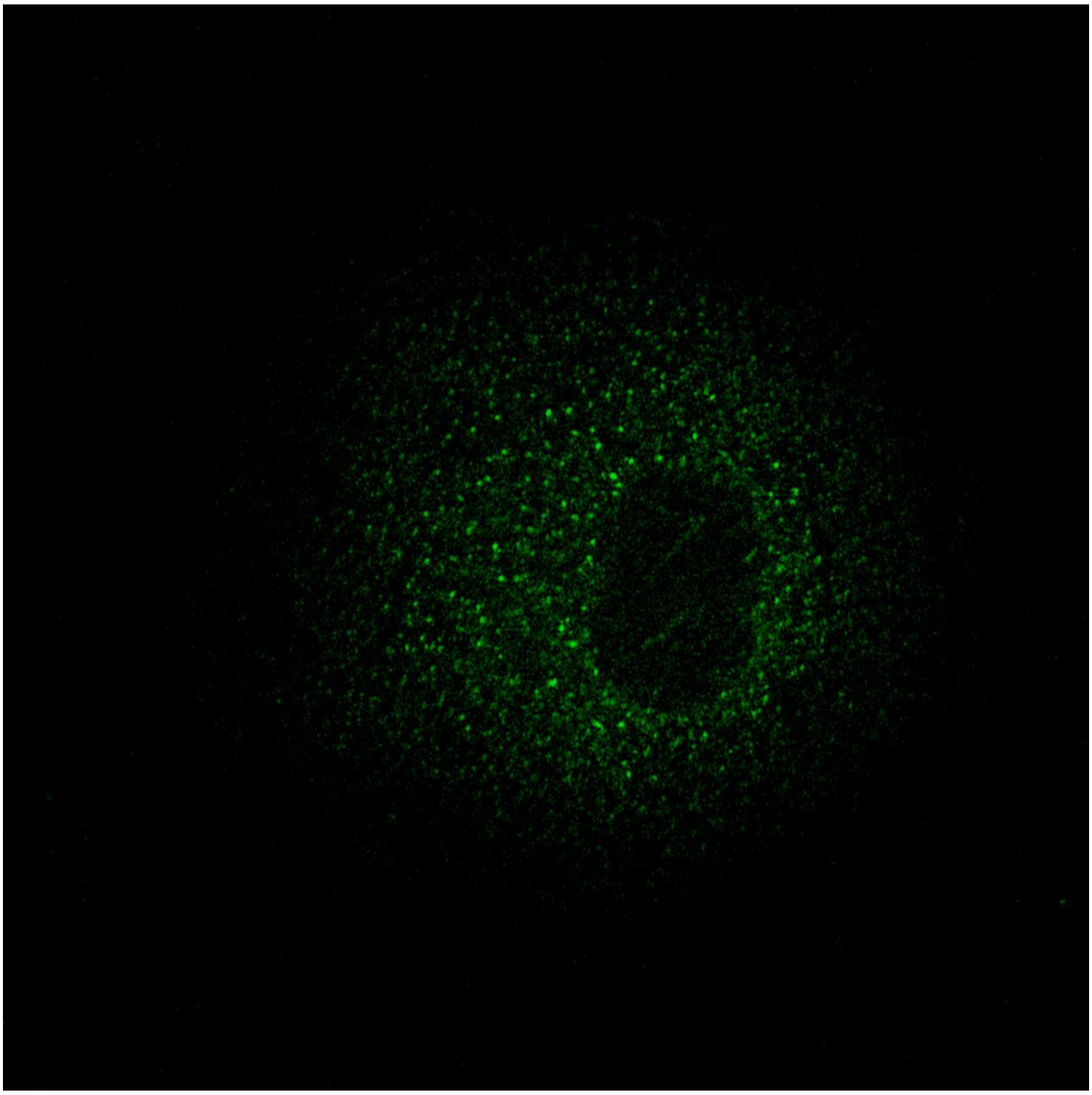

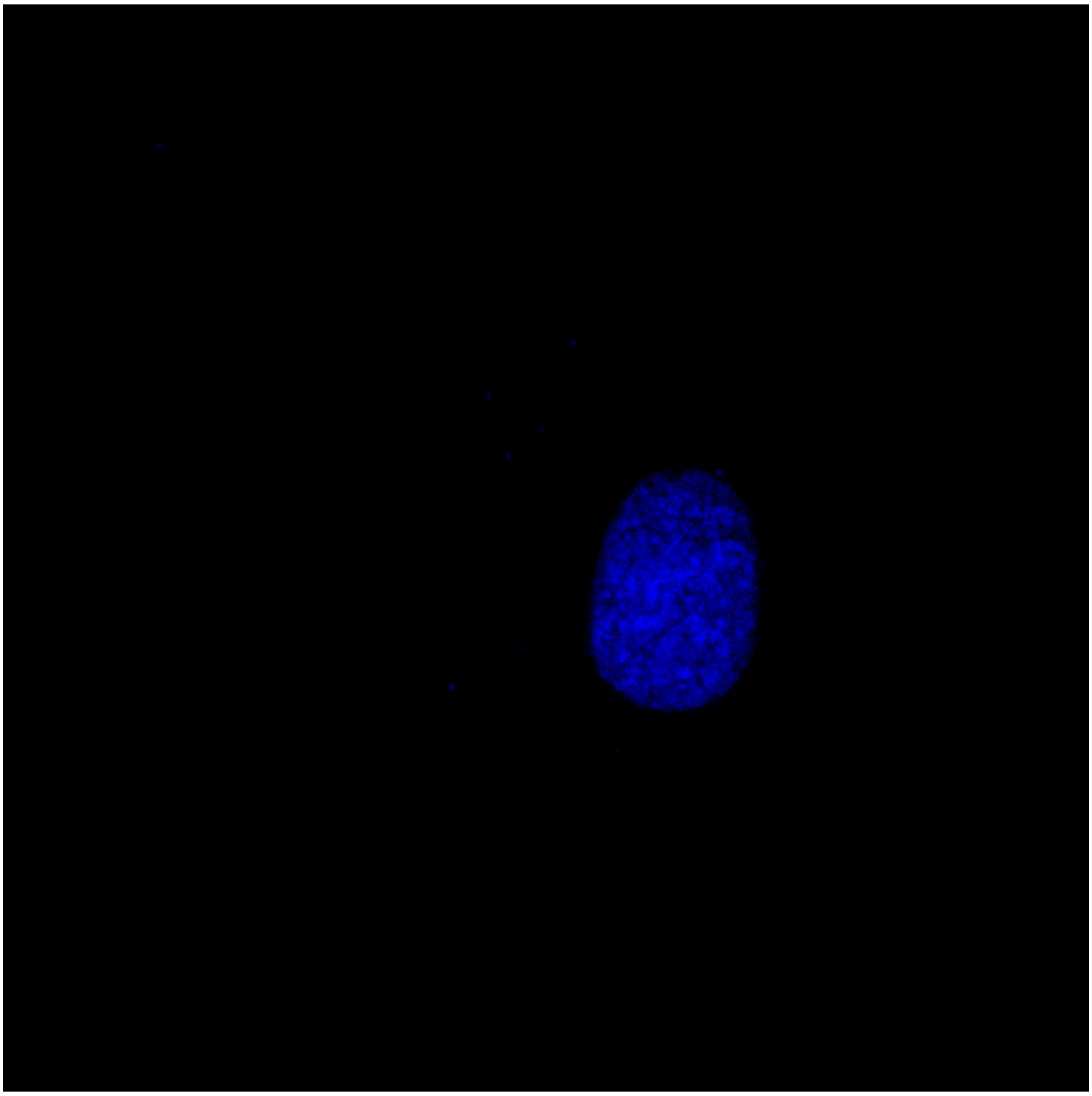

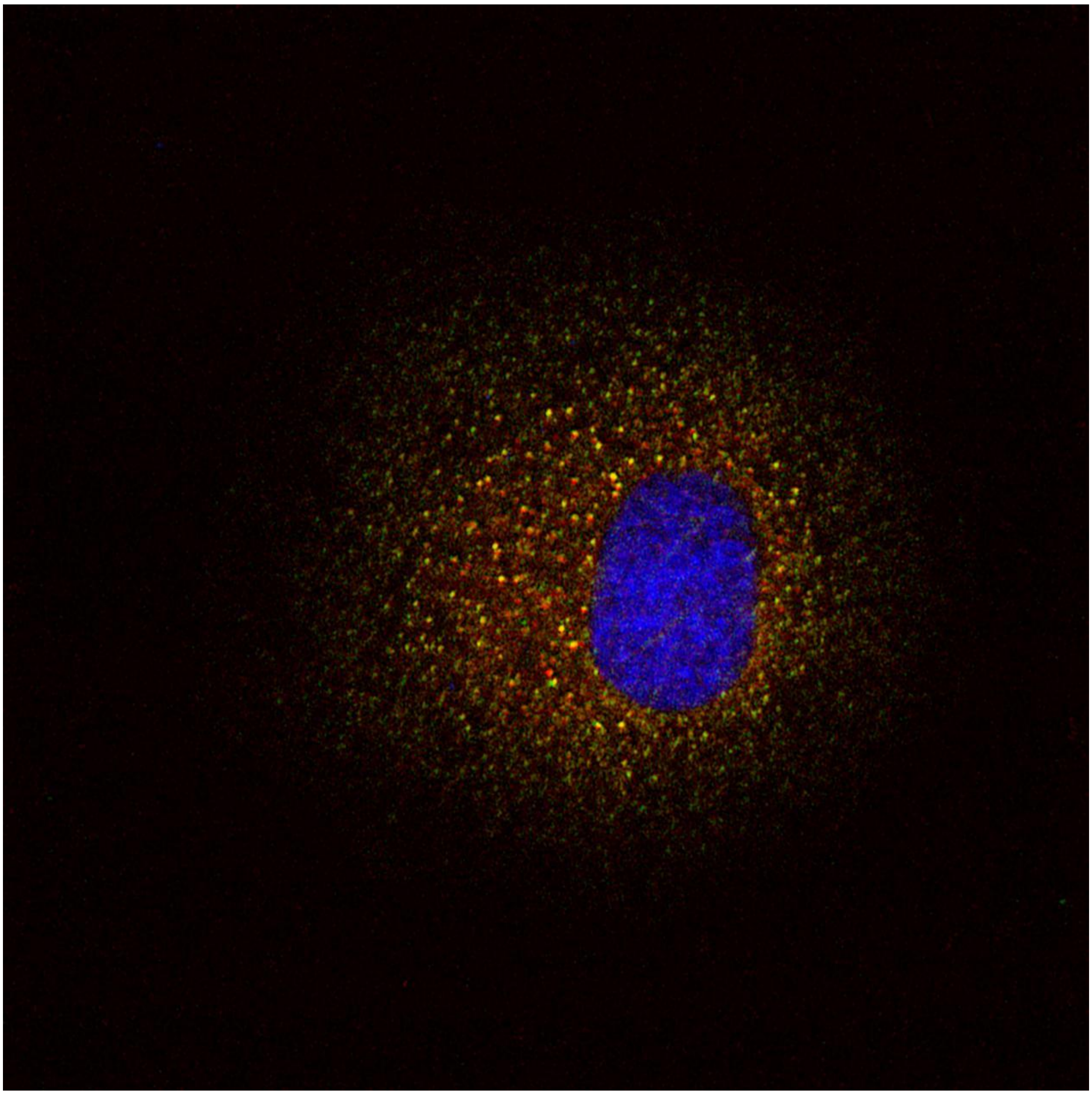

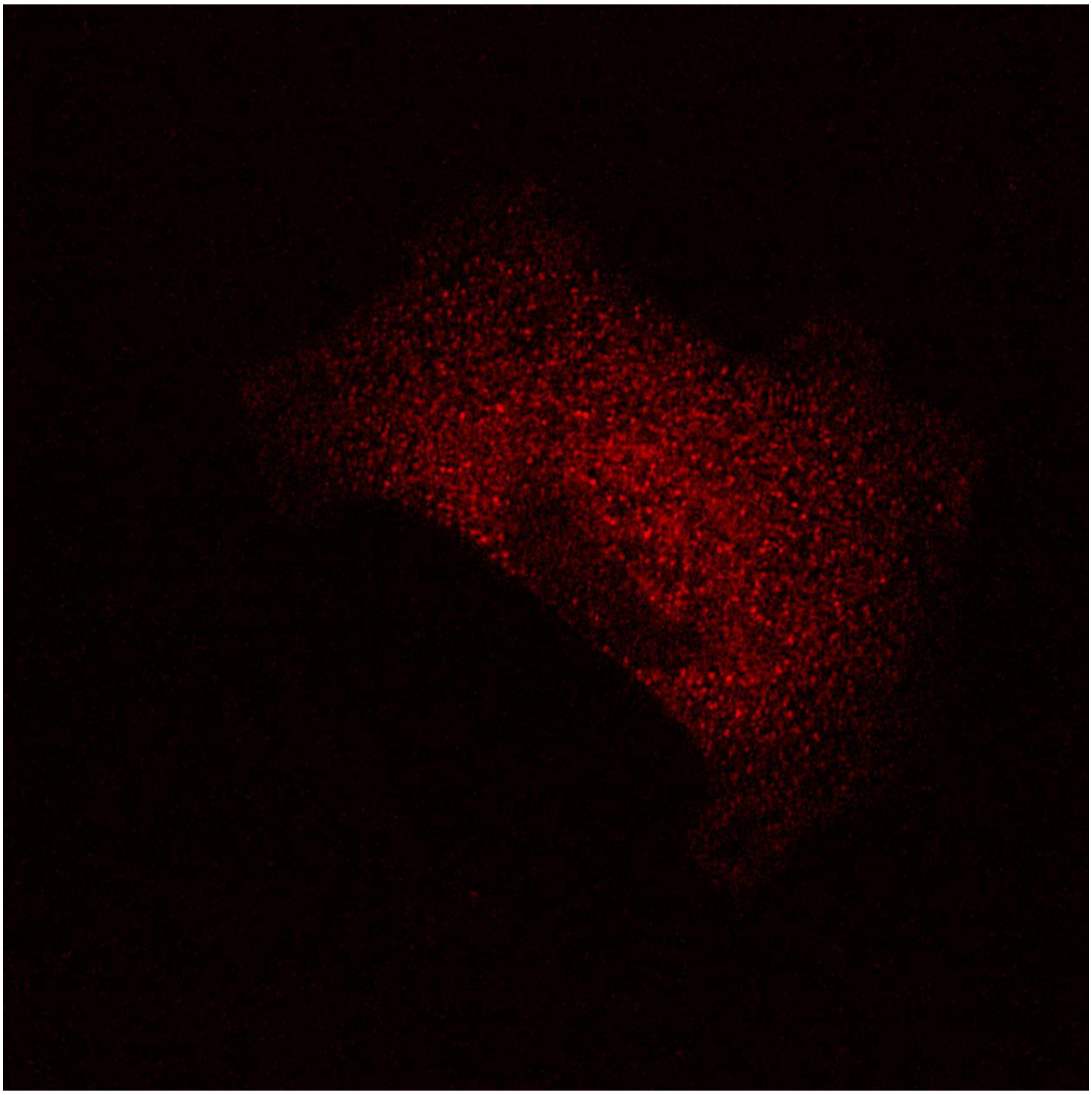

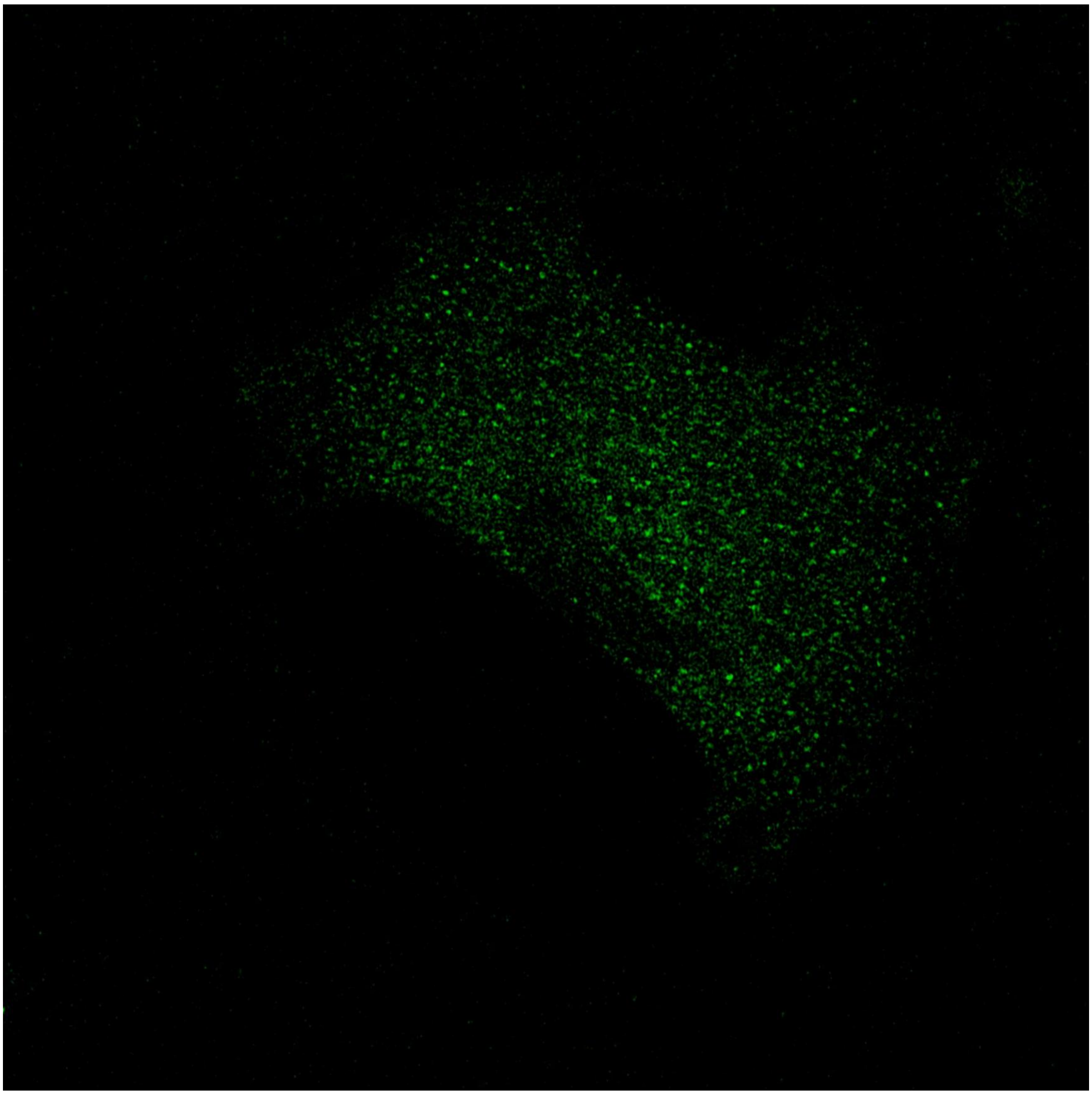

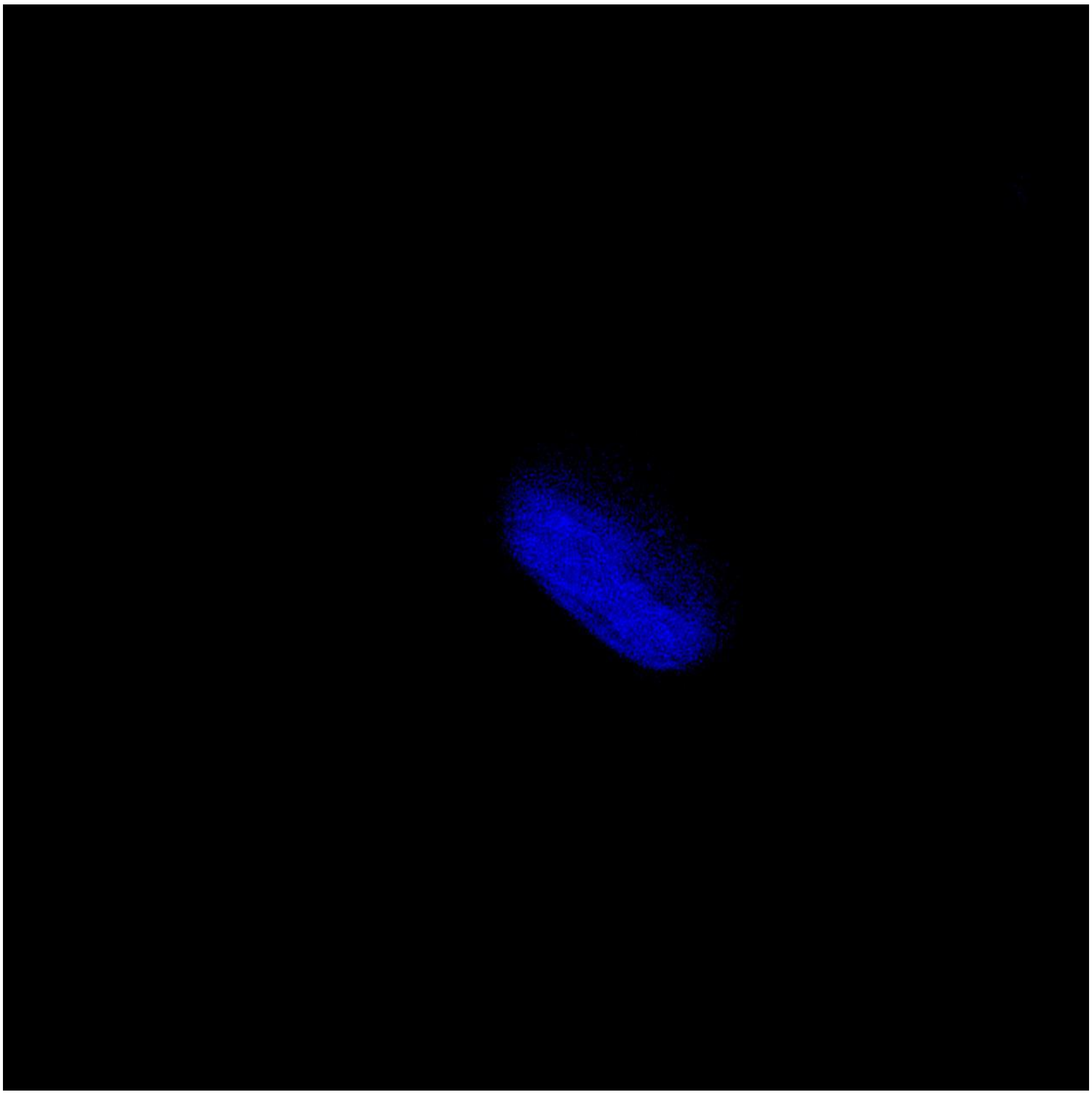

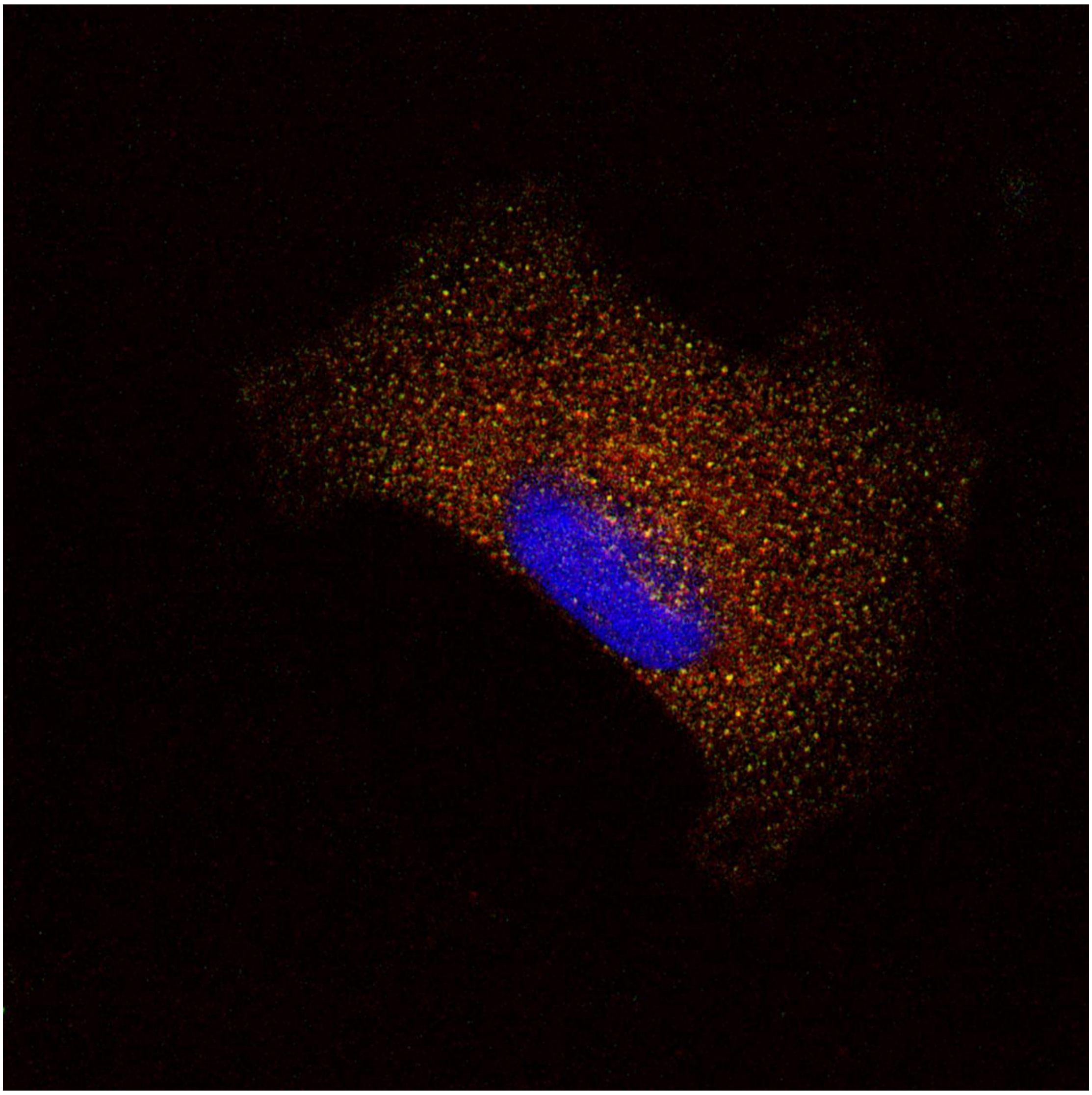

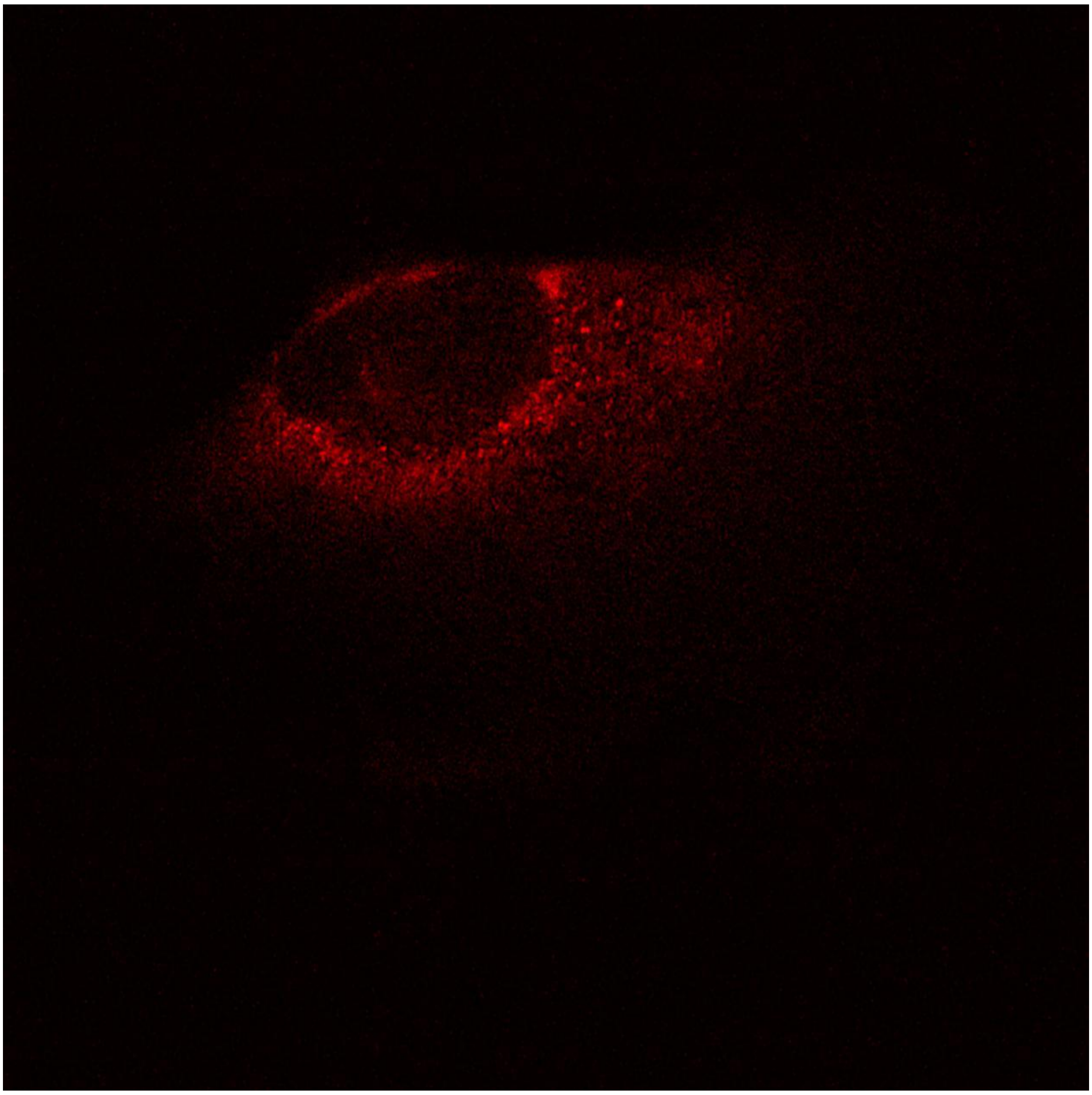

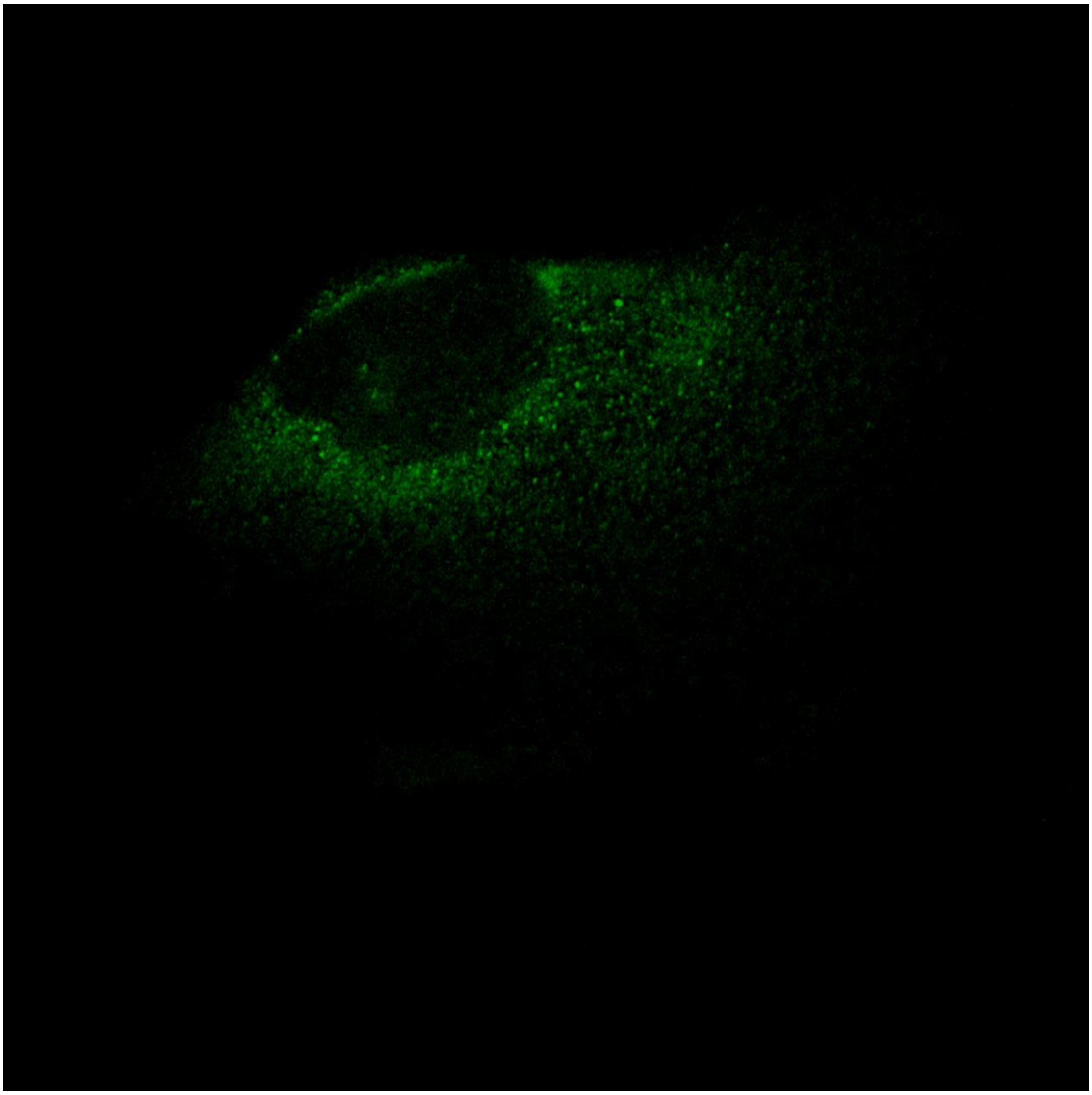

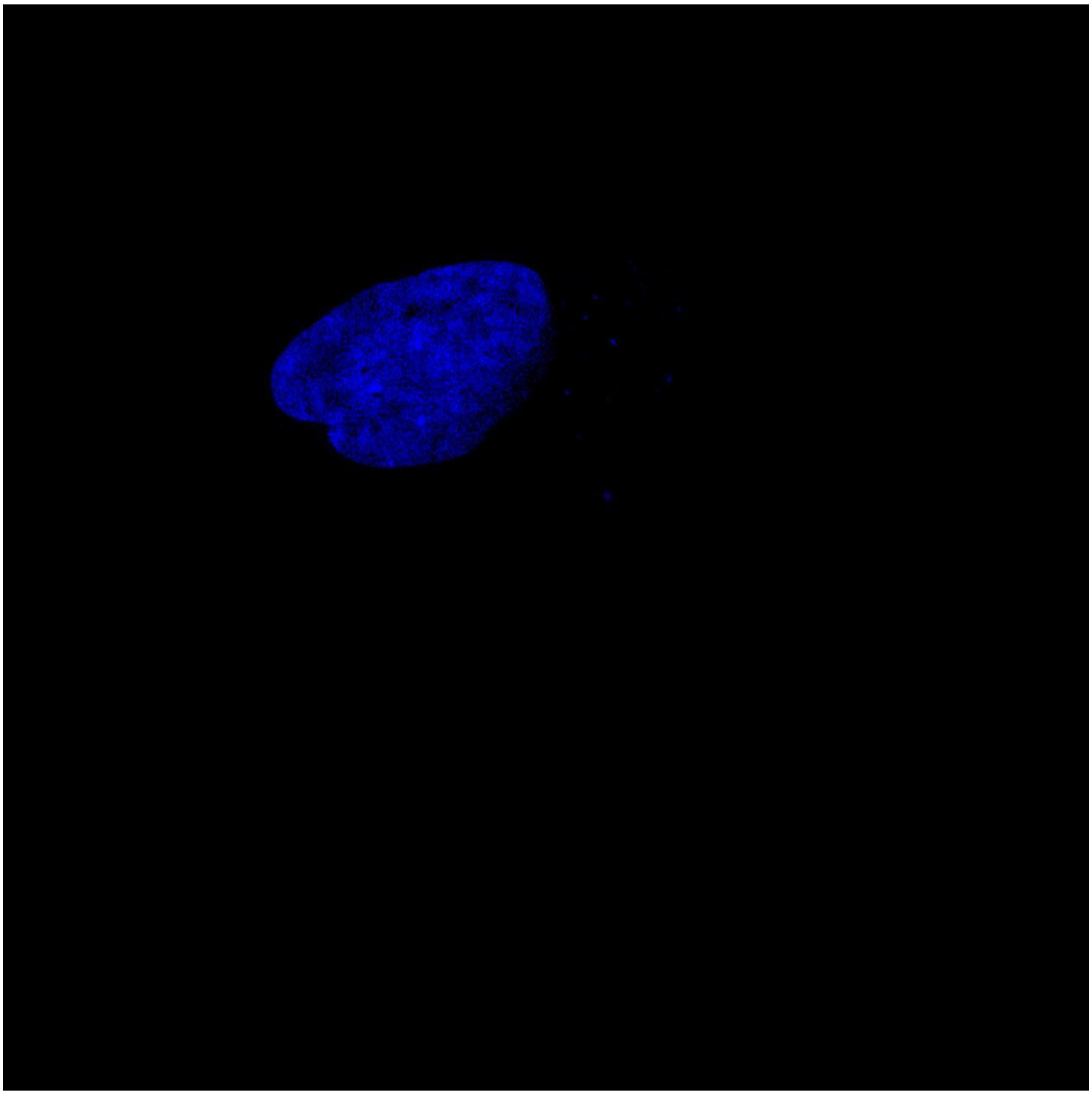

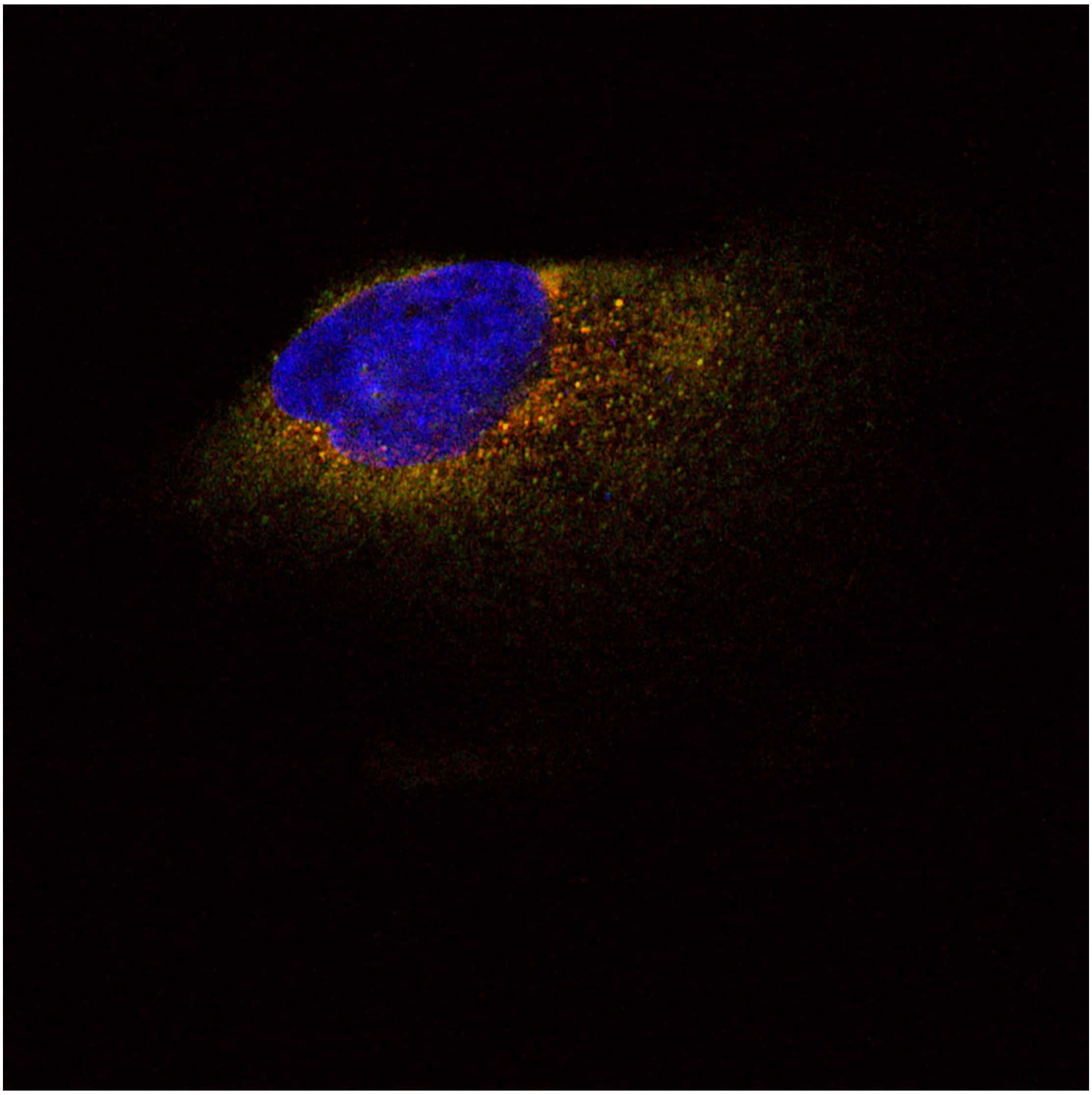

Fig.4E for LPS+GHS+miR-NC group

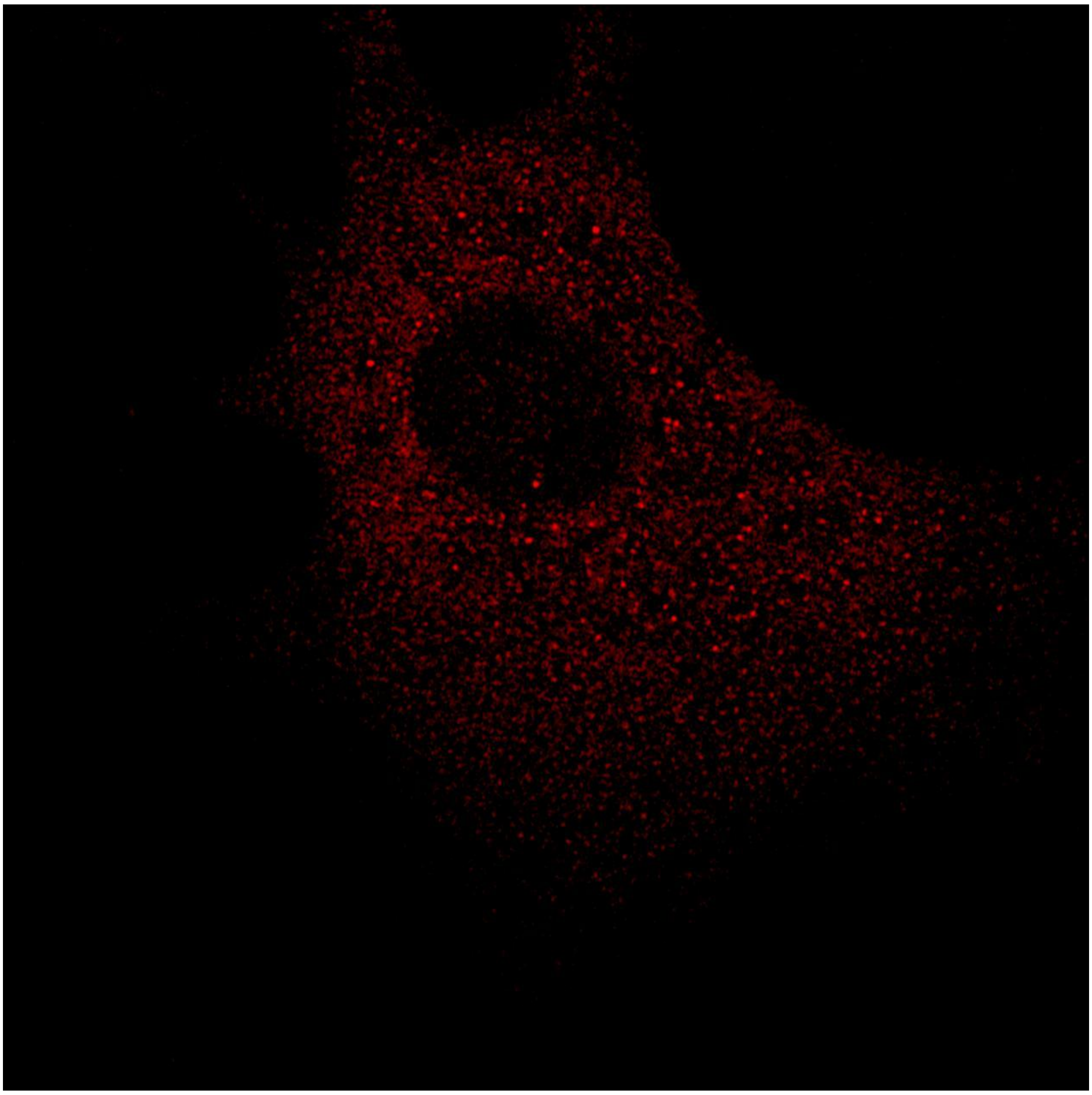

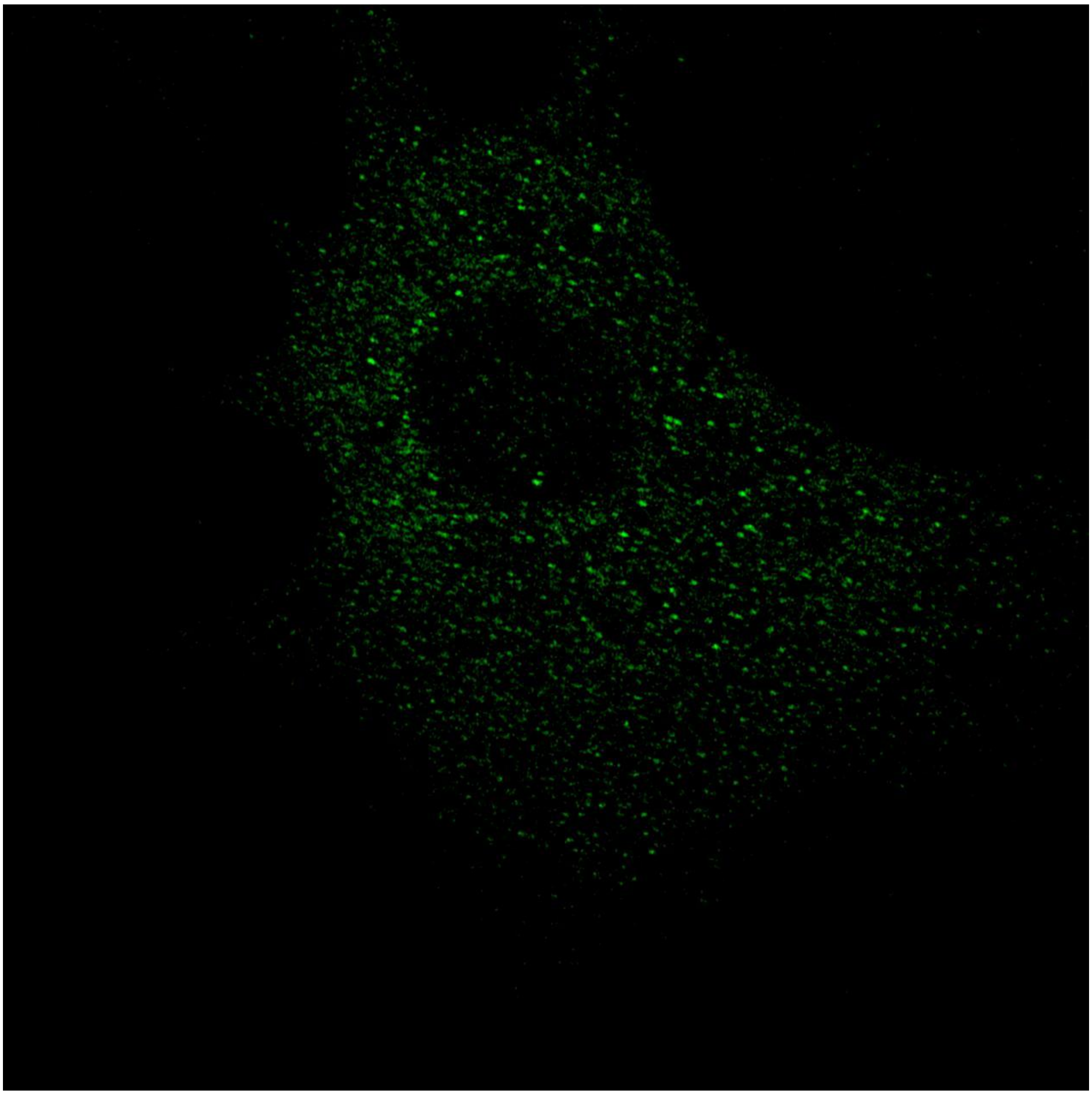

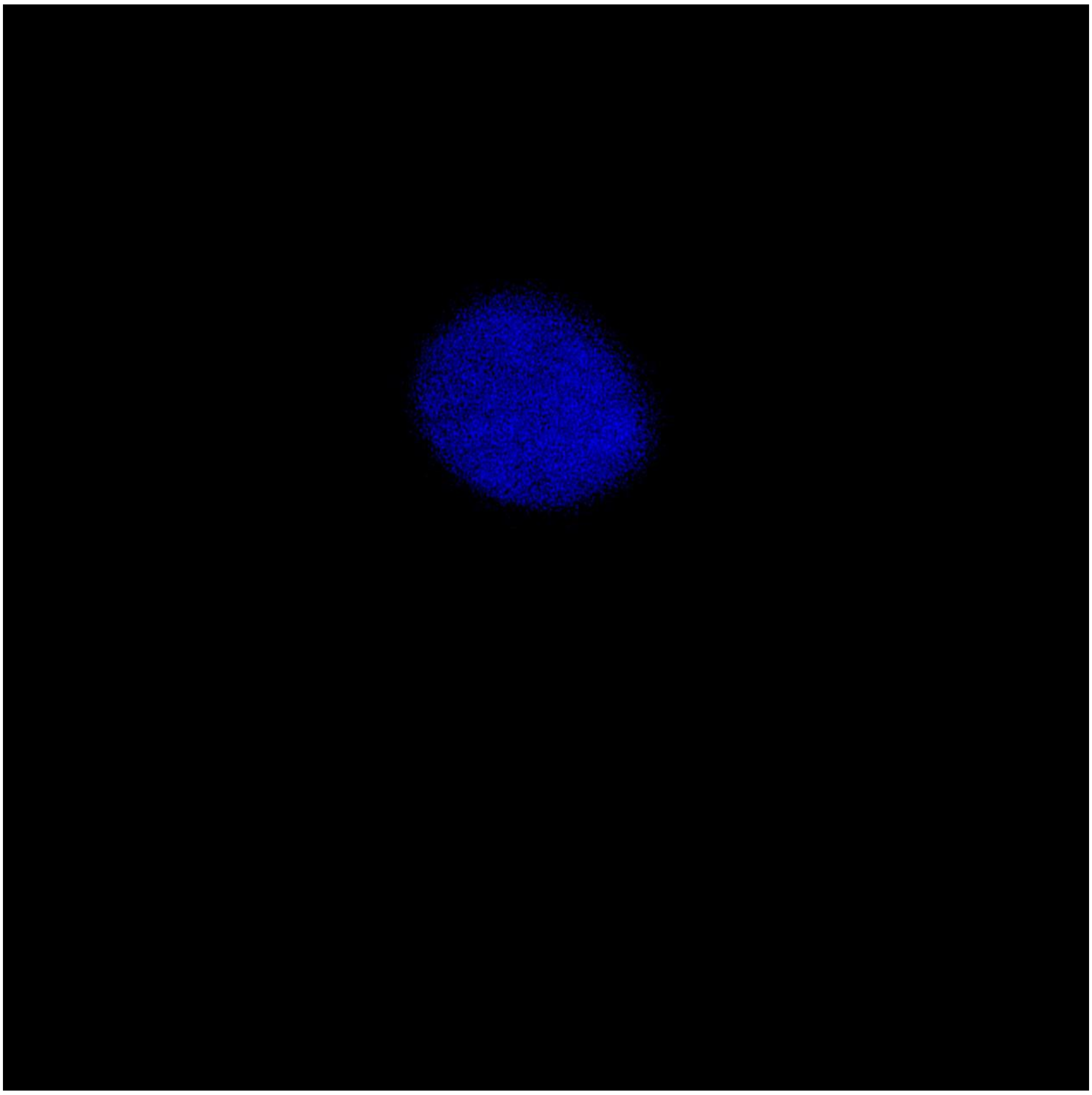

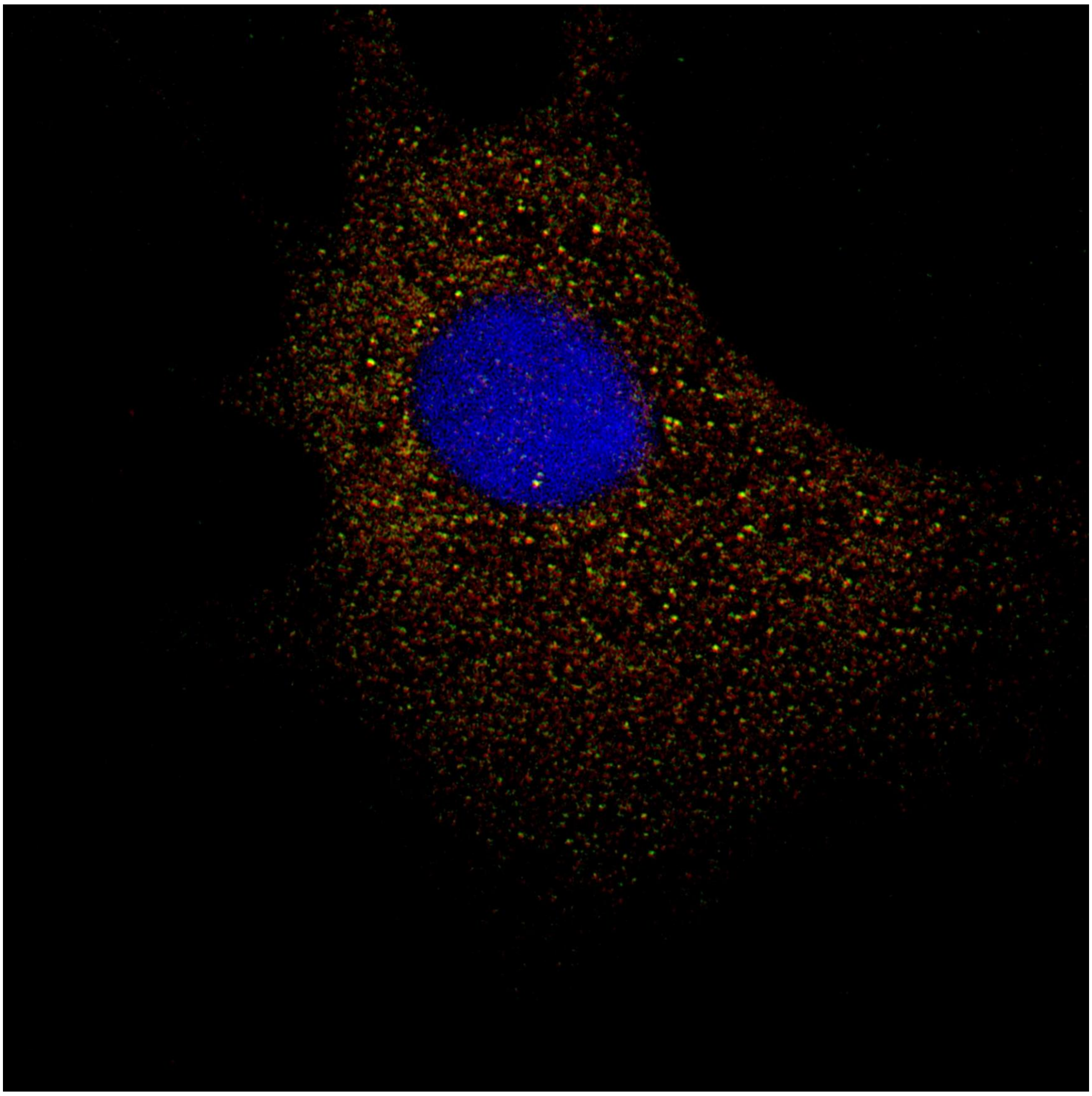

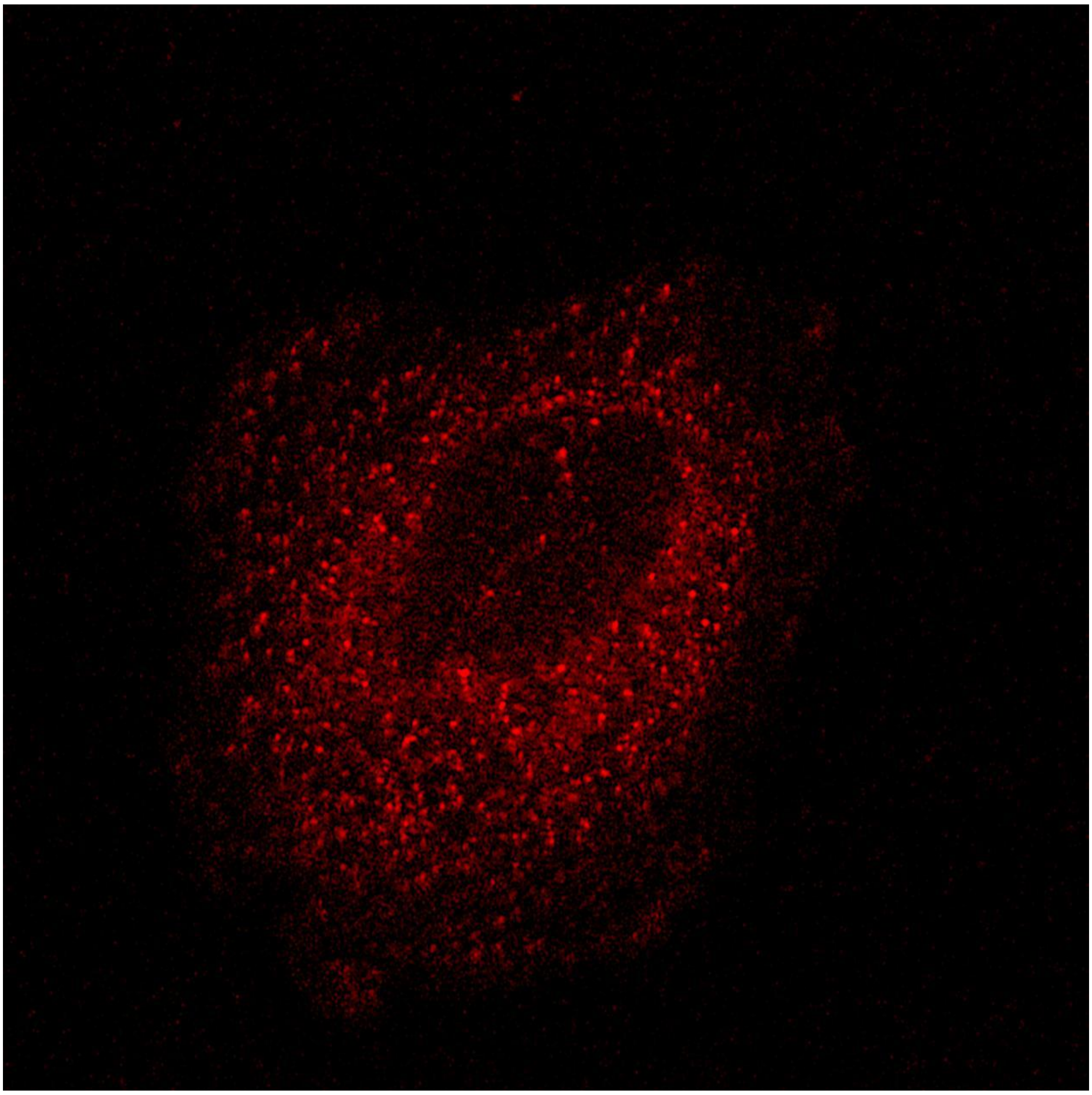

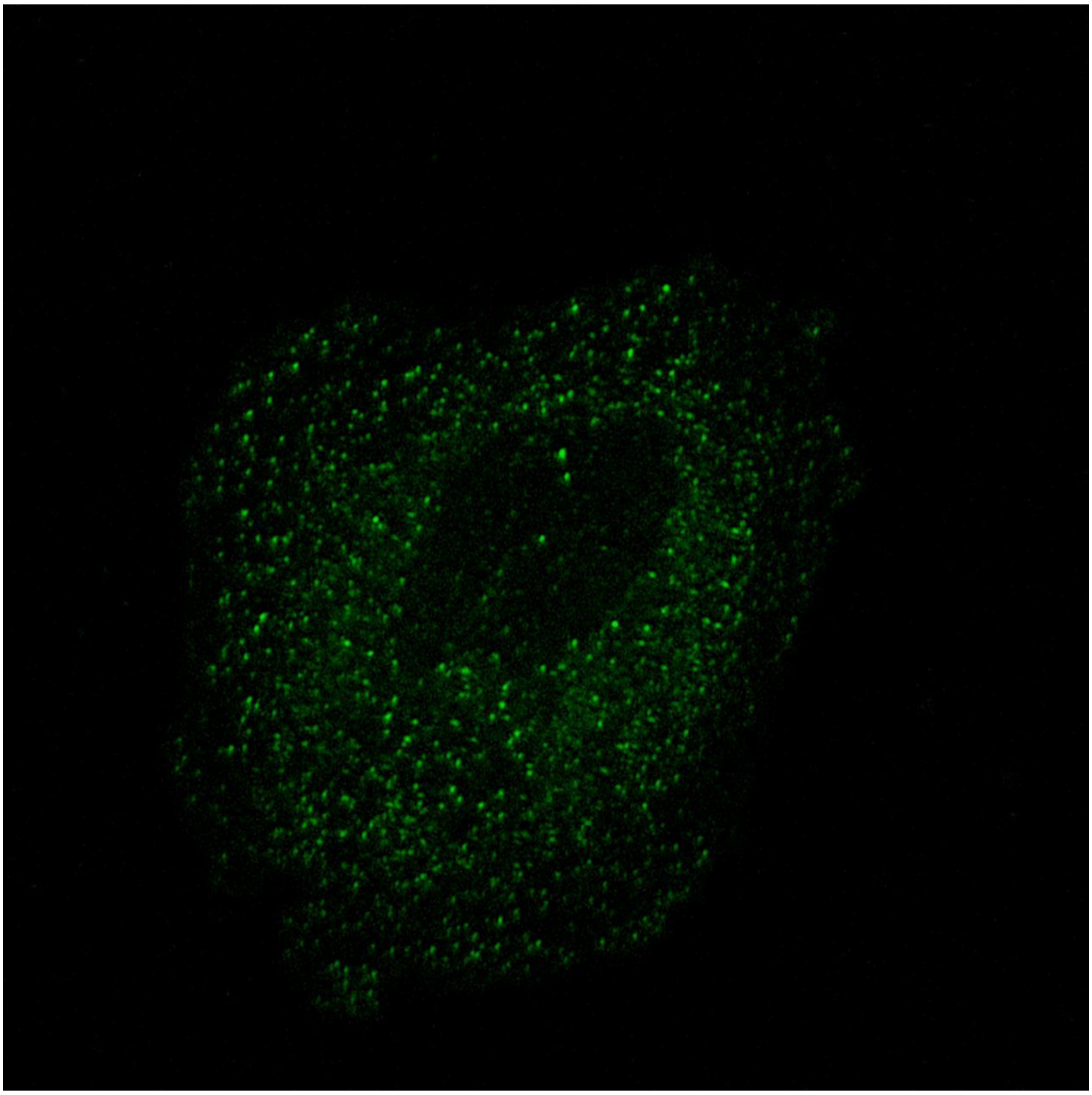

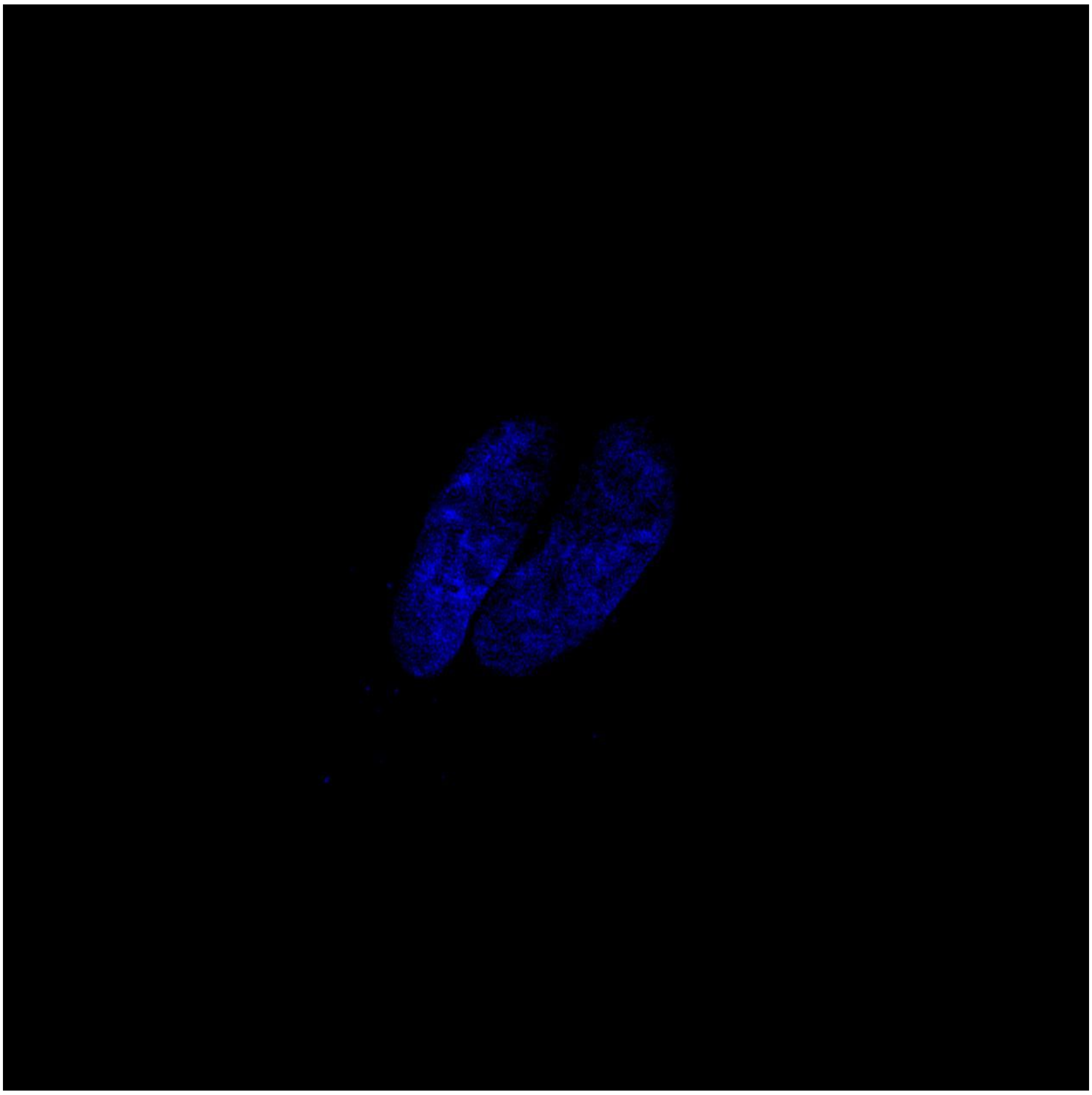

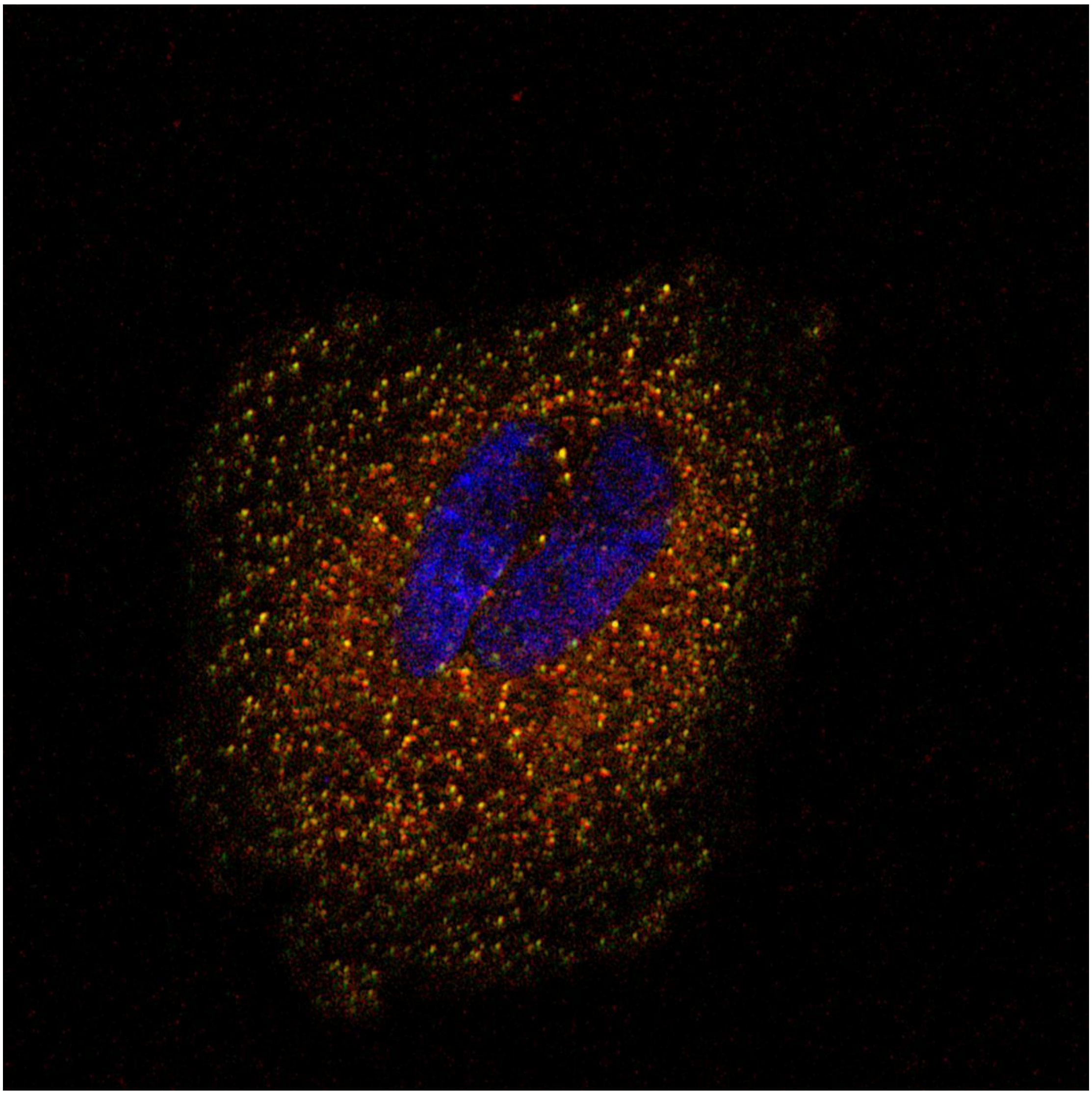

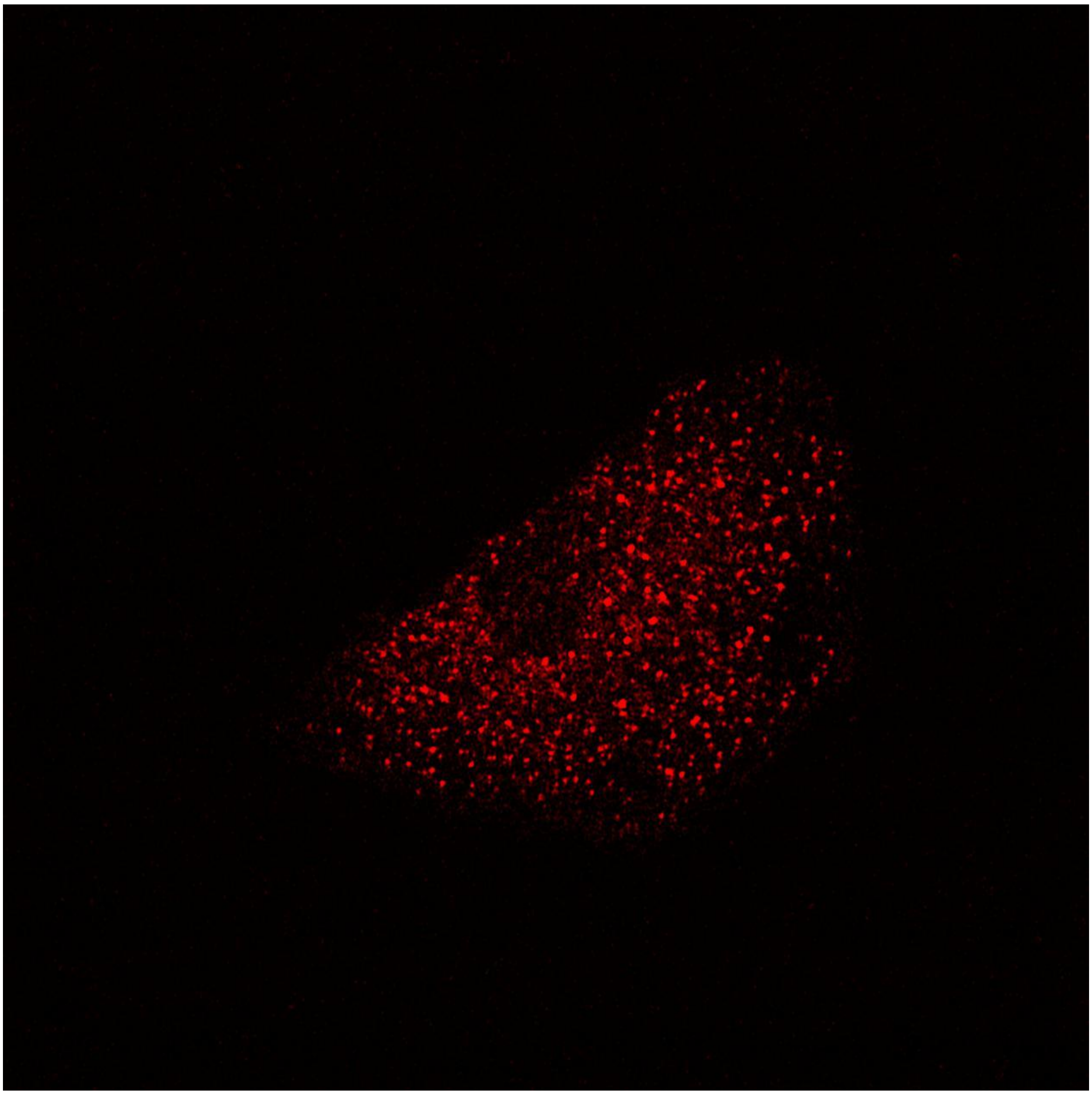

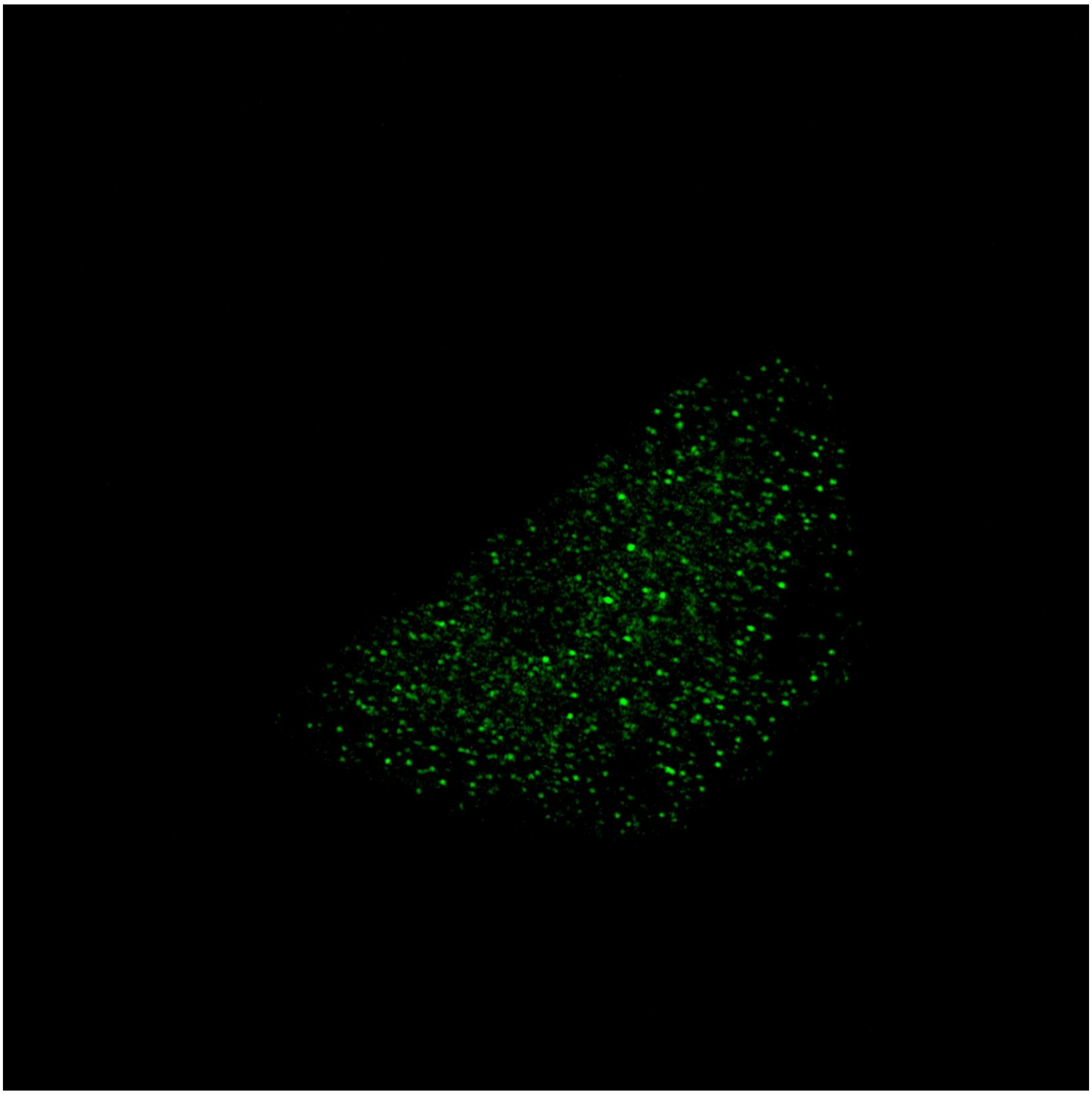

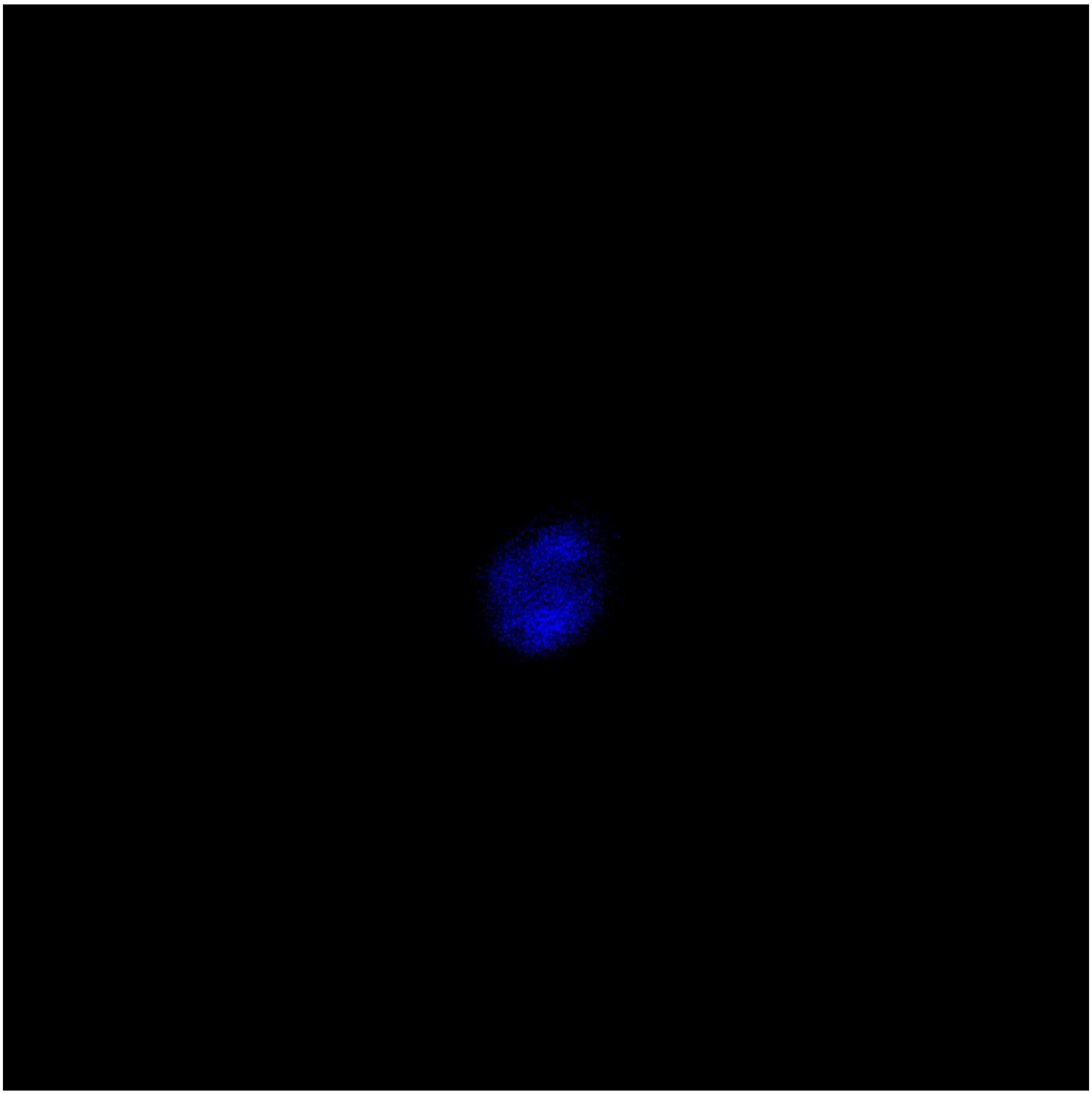

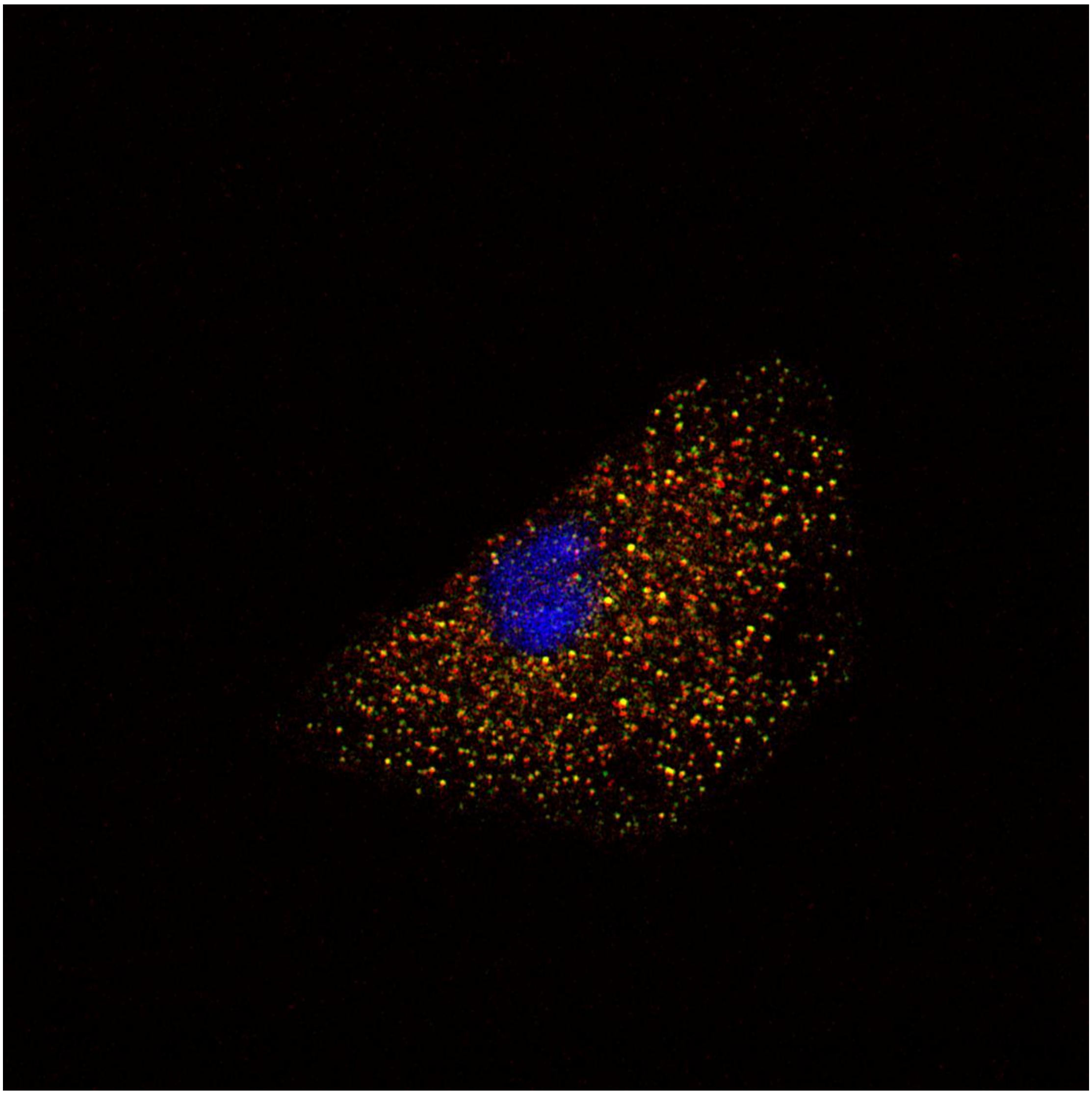

Fig.4E for LPS+GHS+miR-143 group

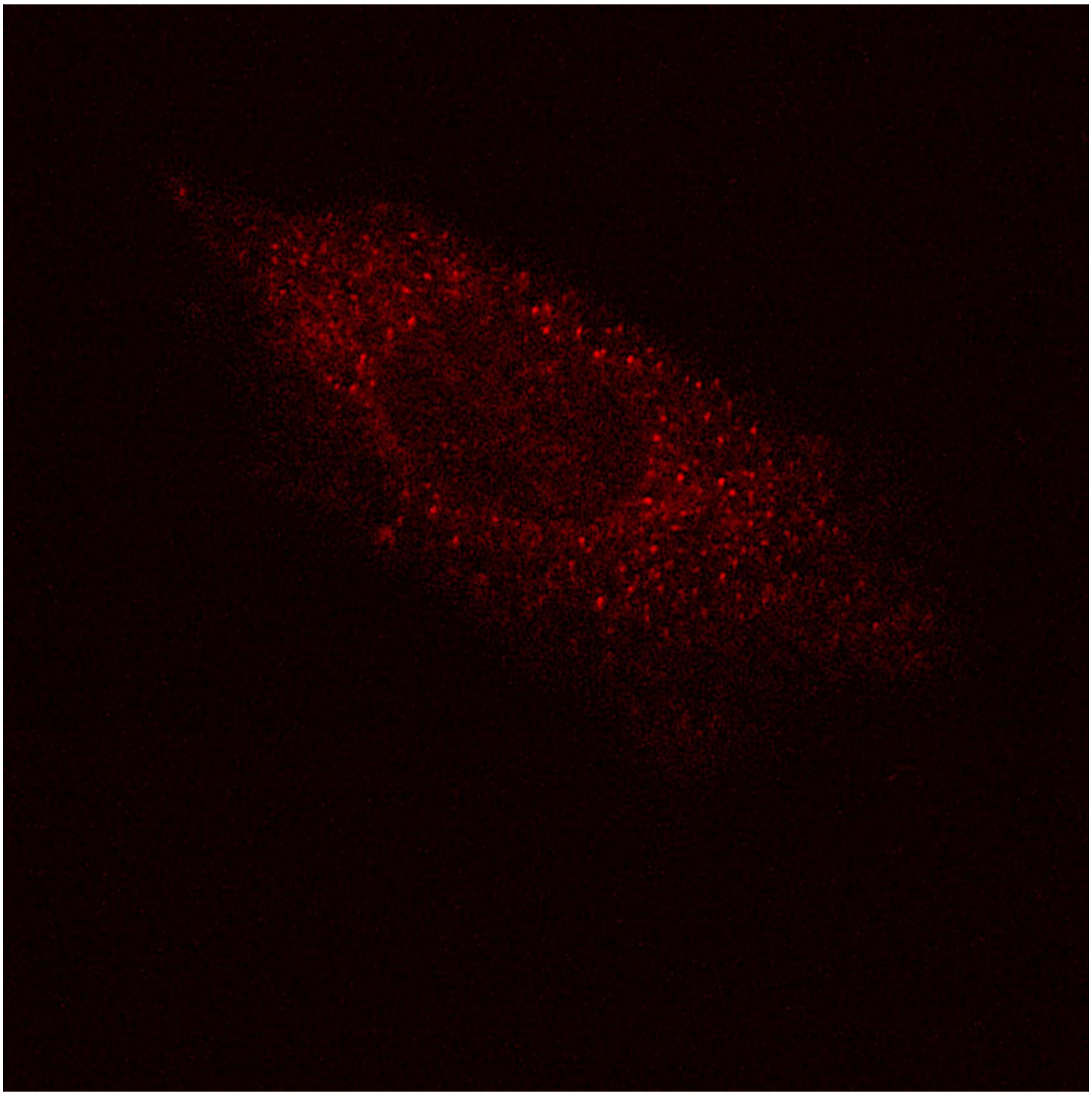

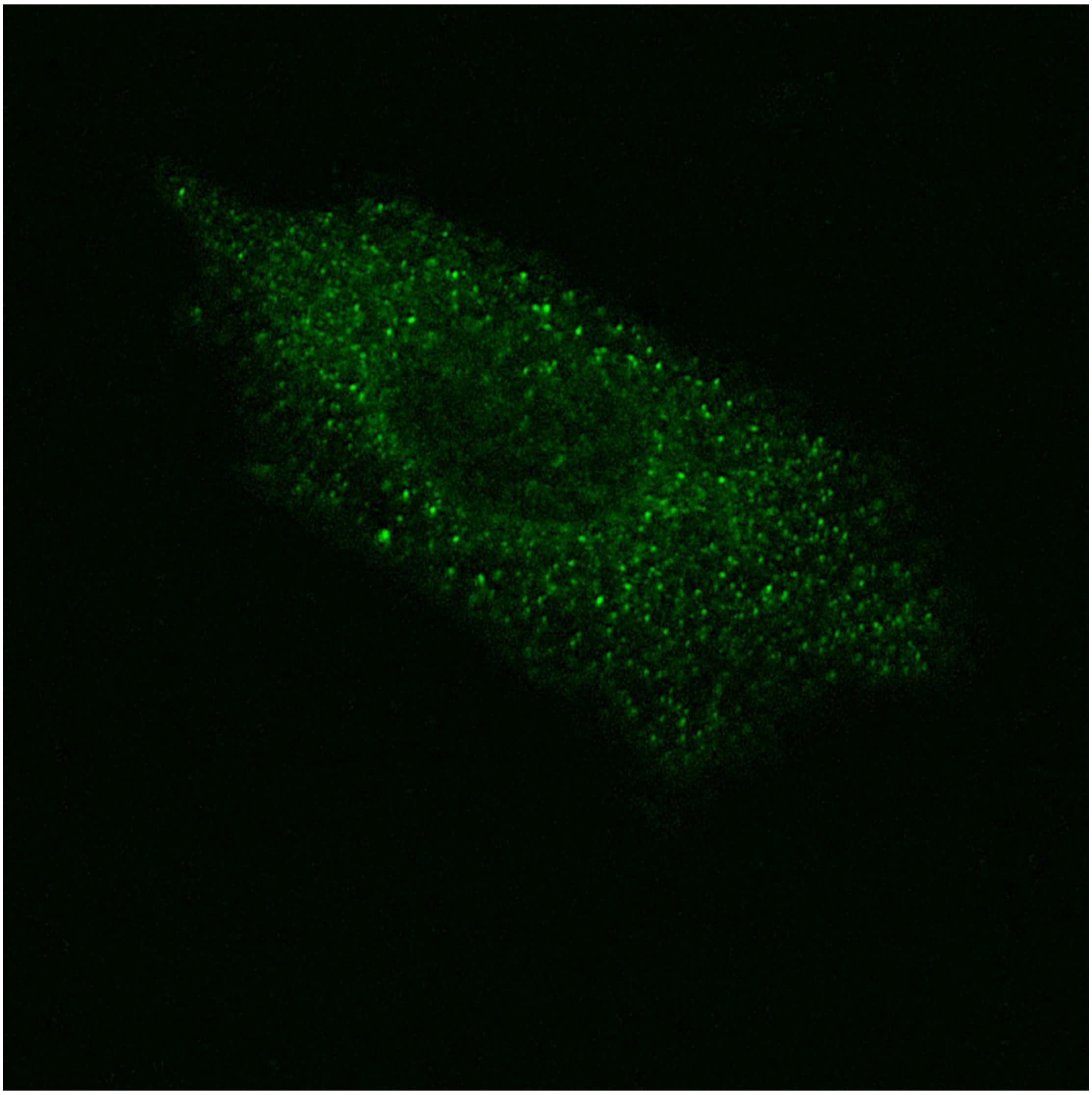

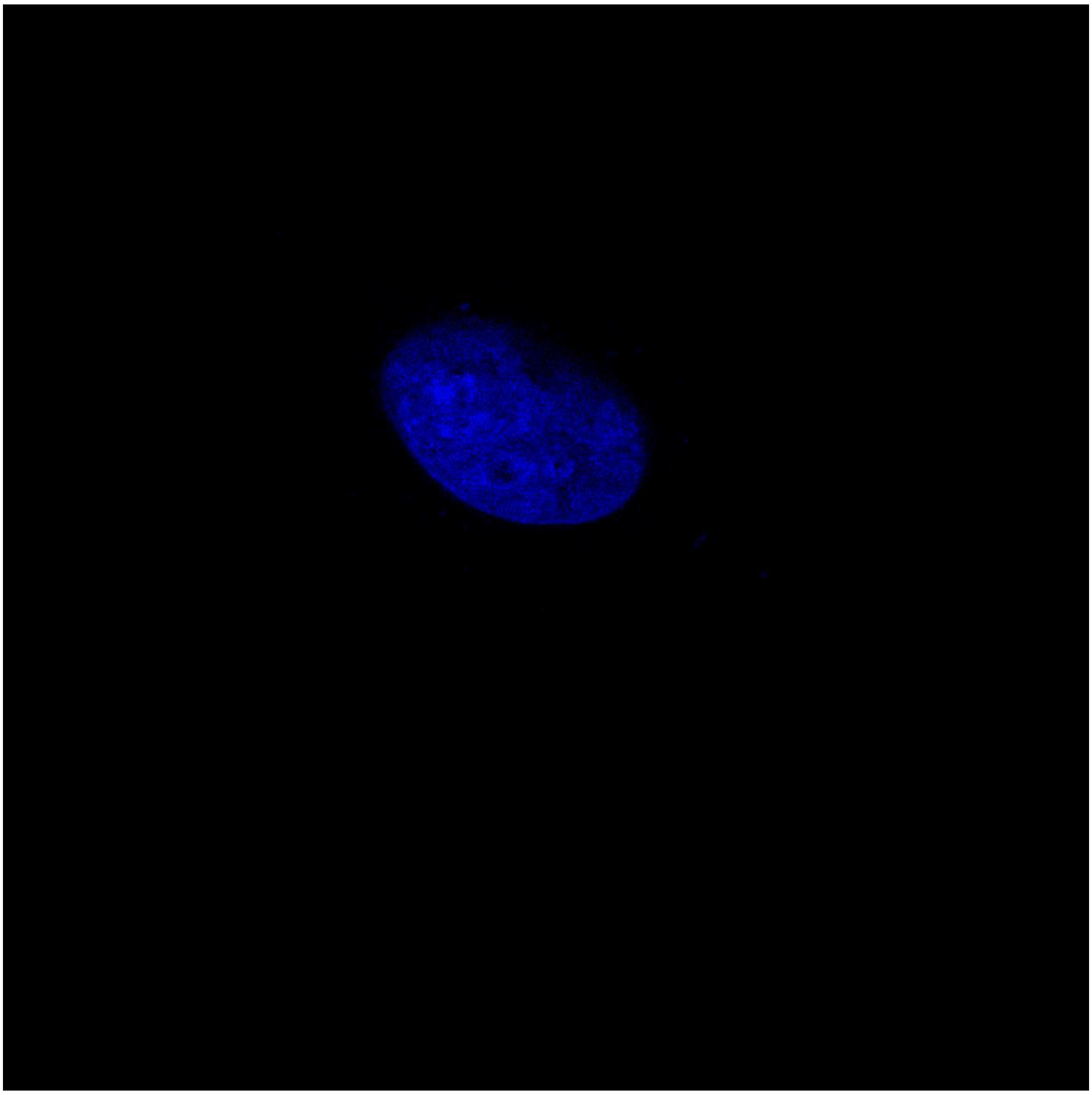

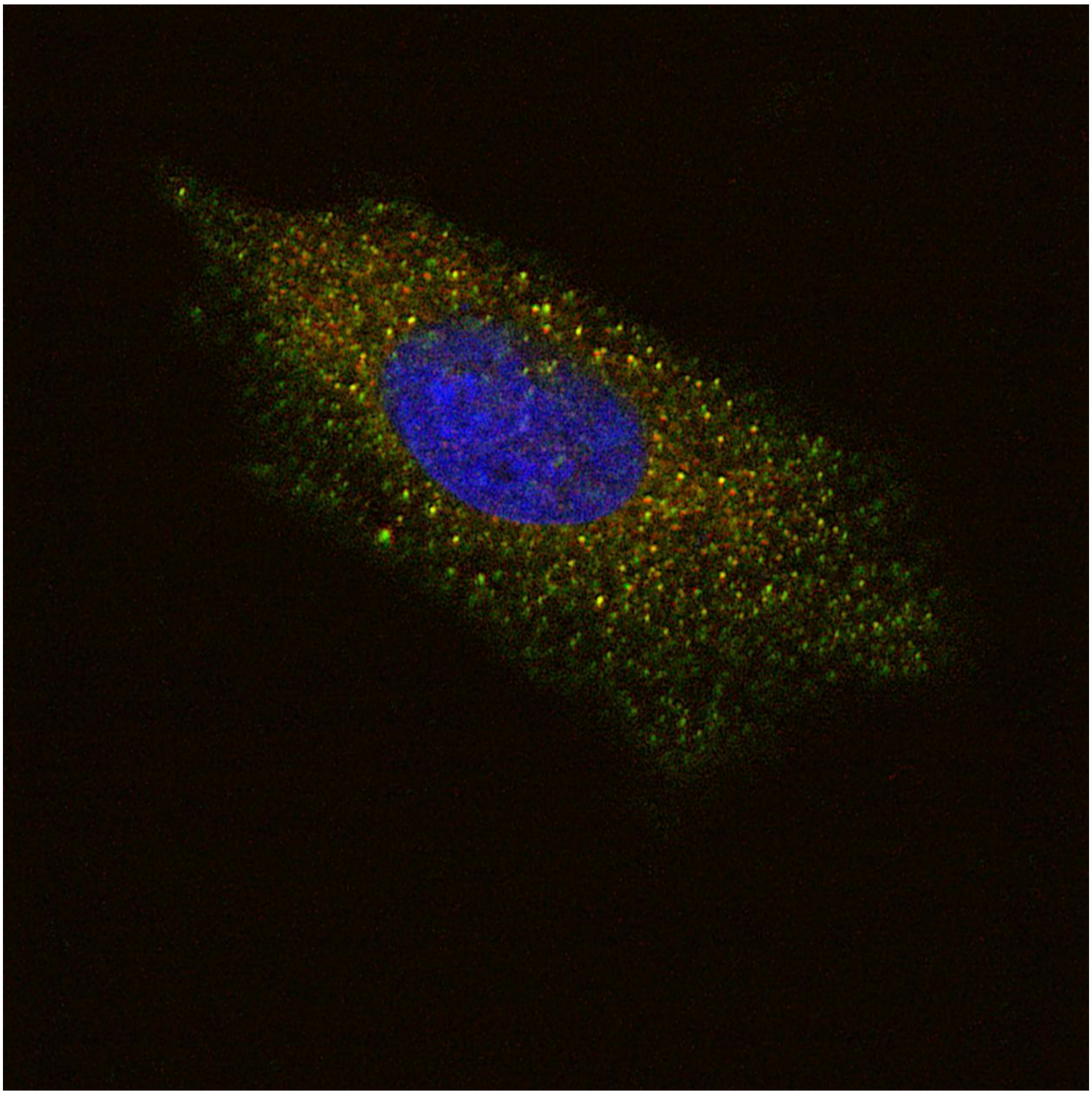

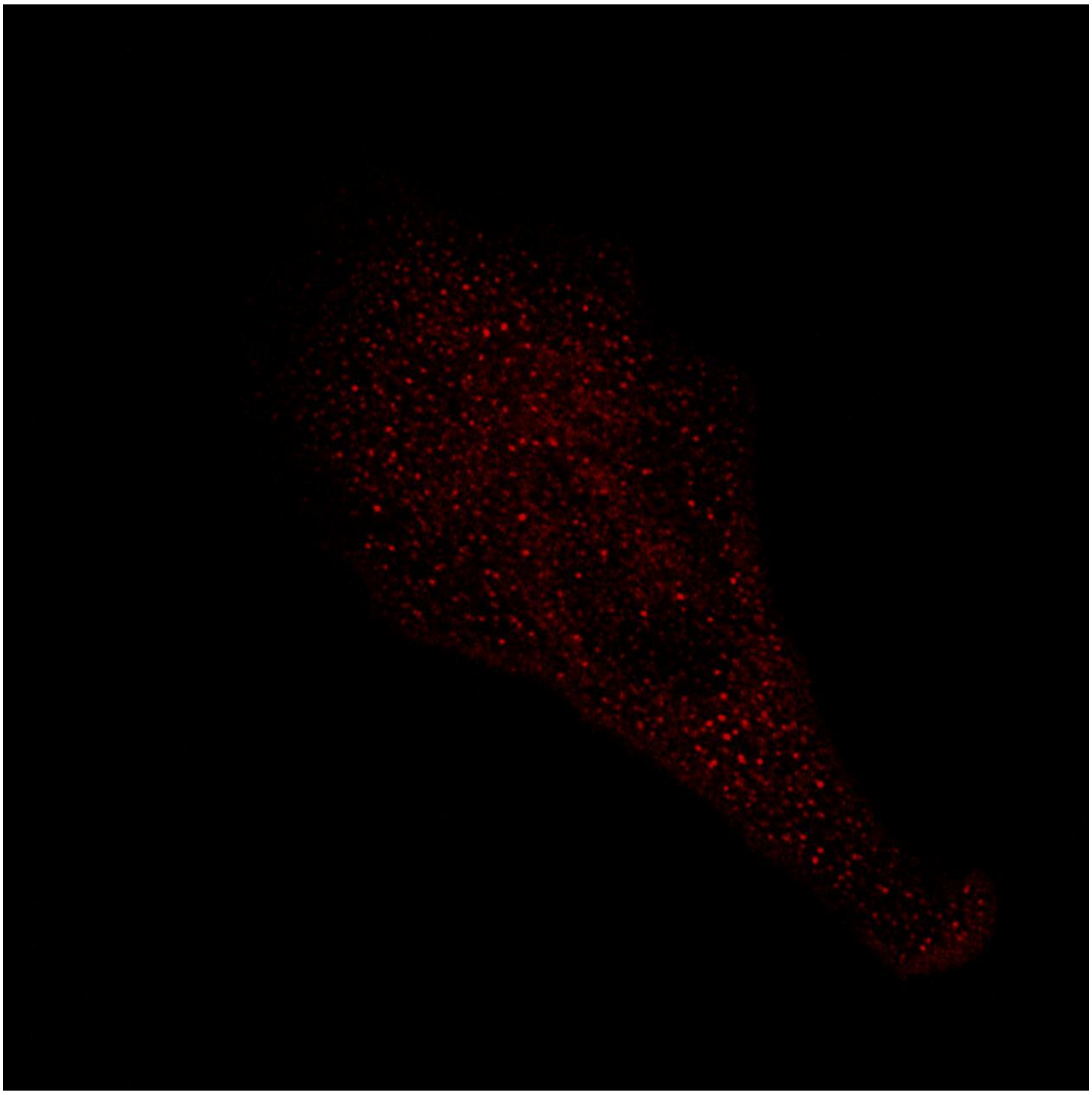

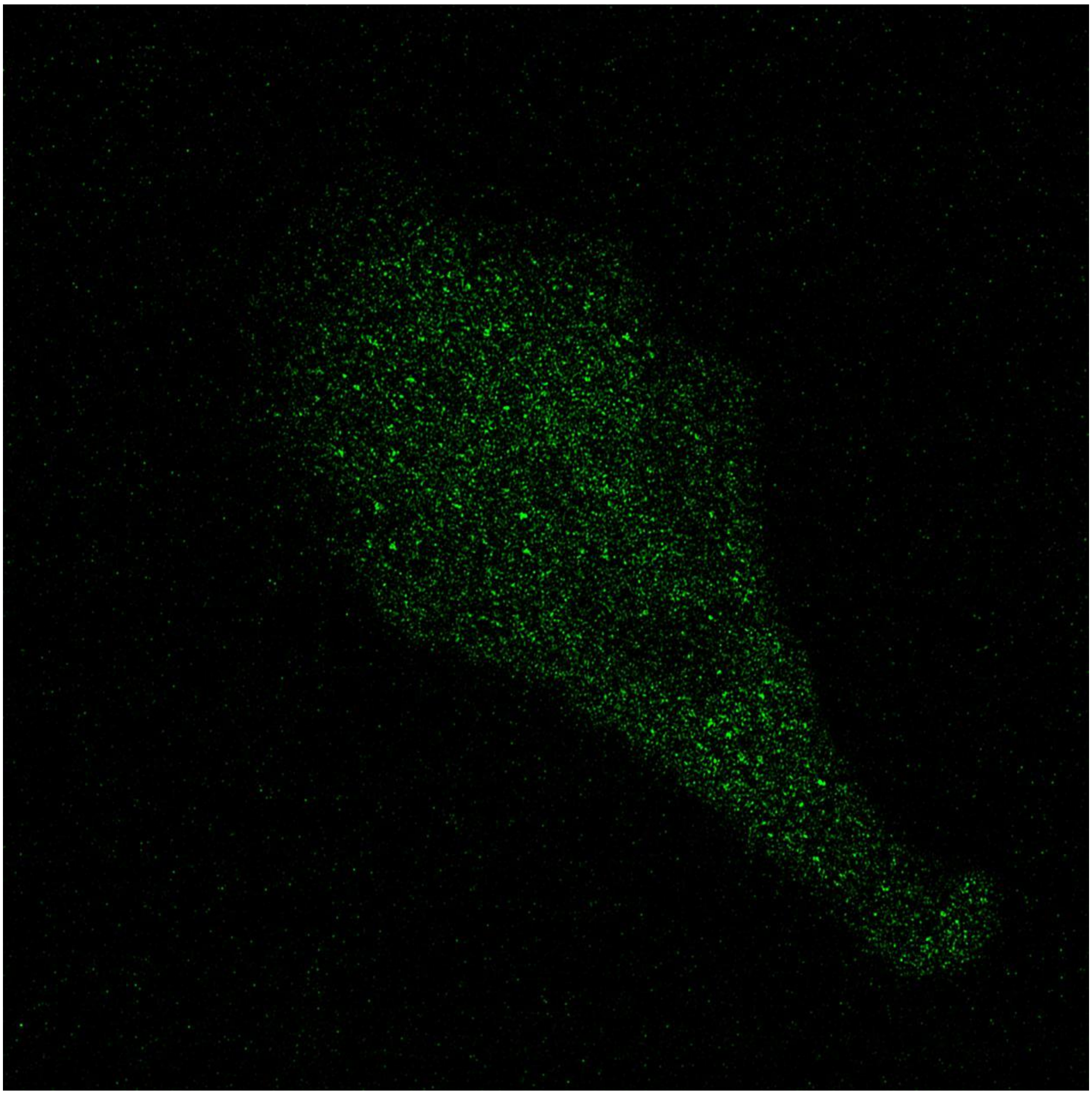

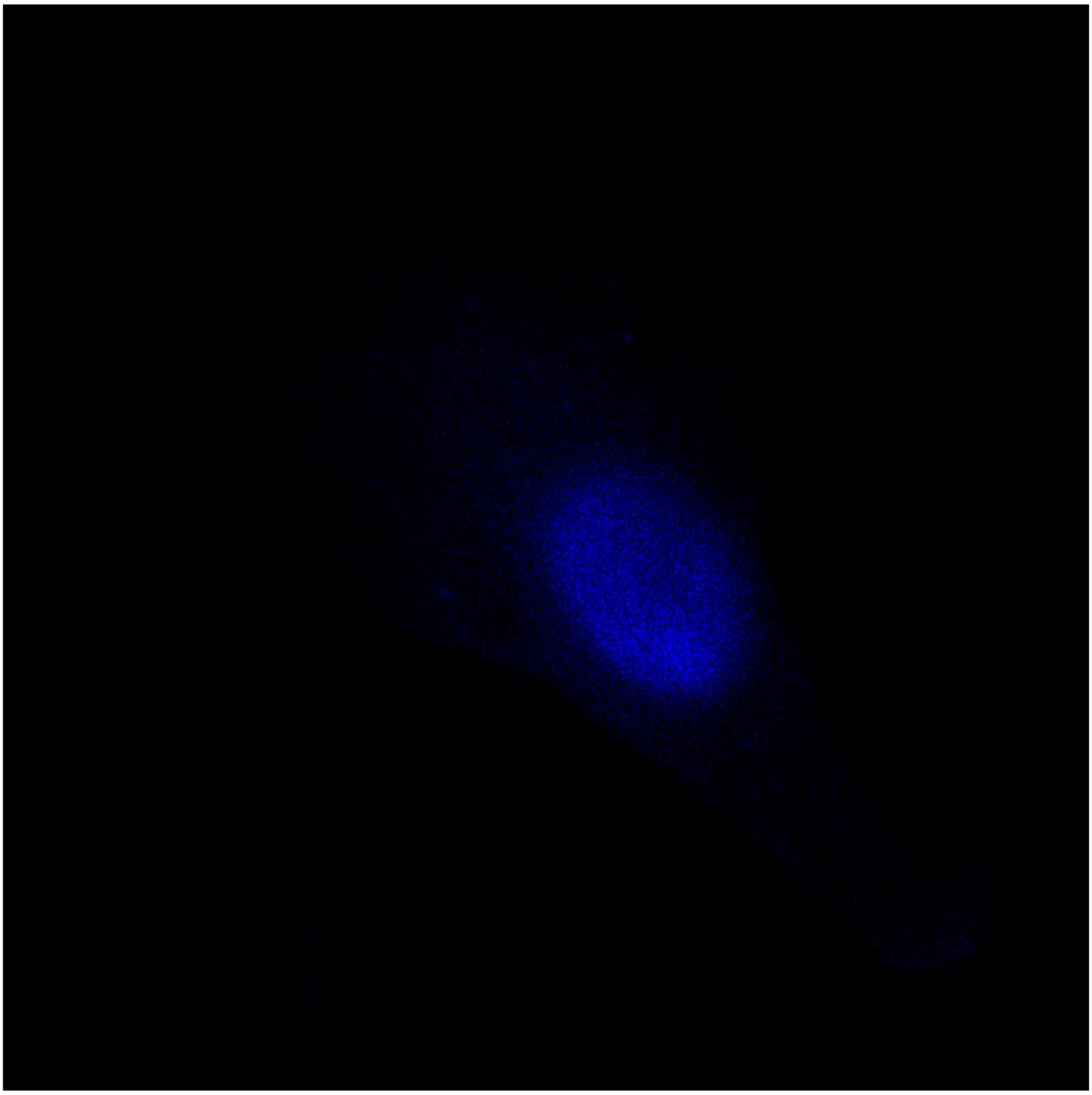

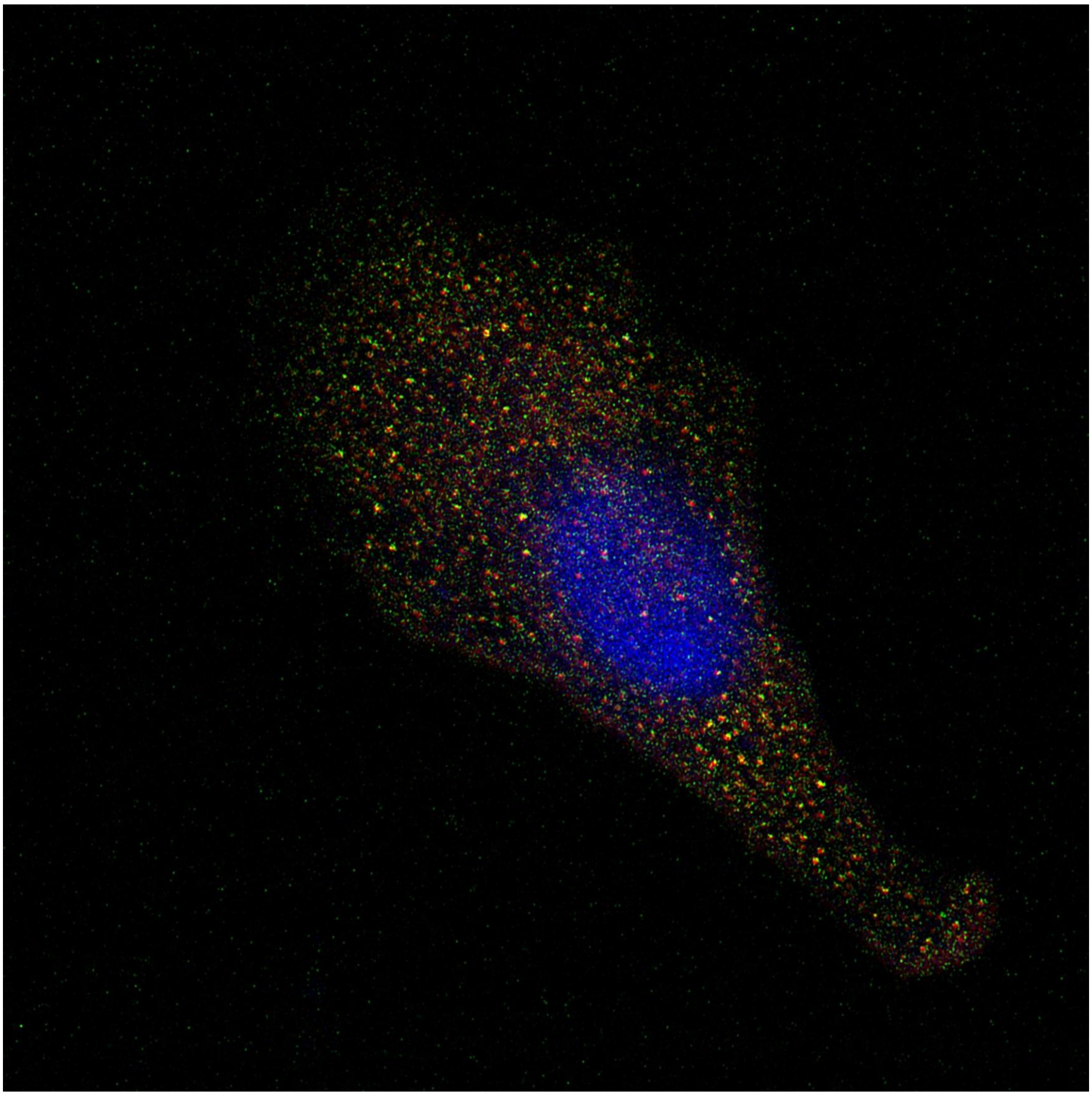

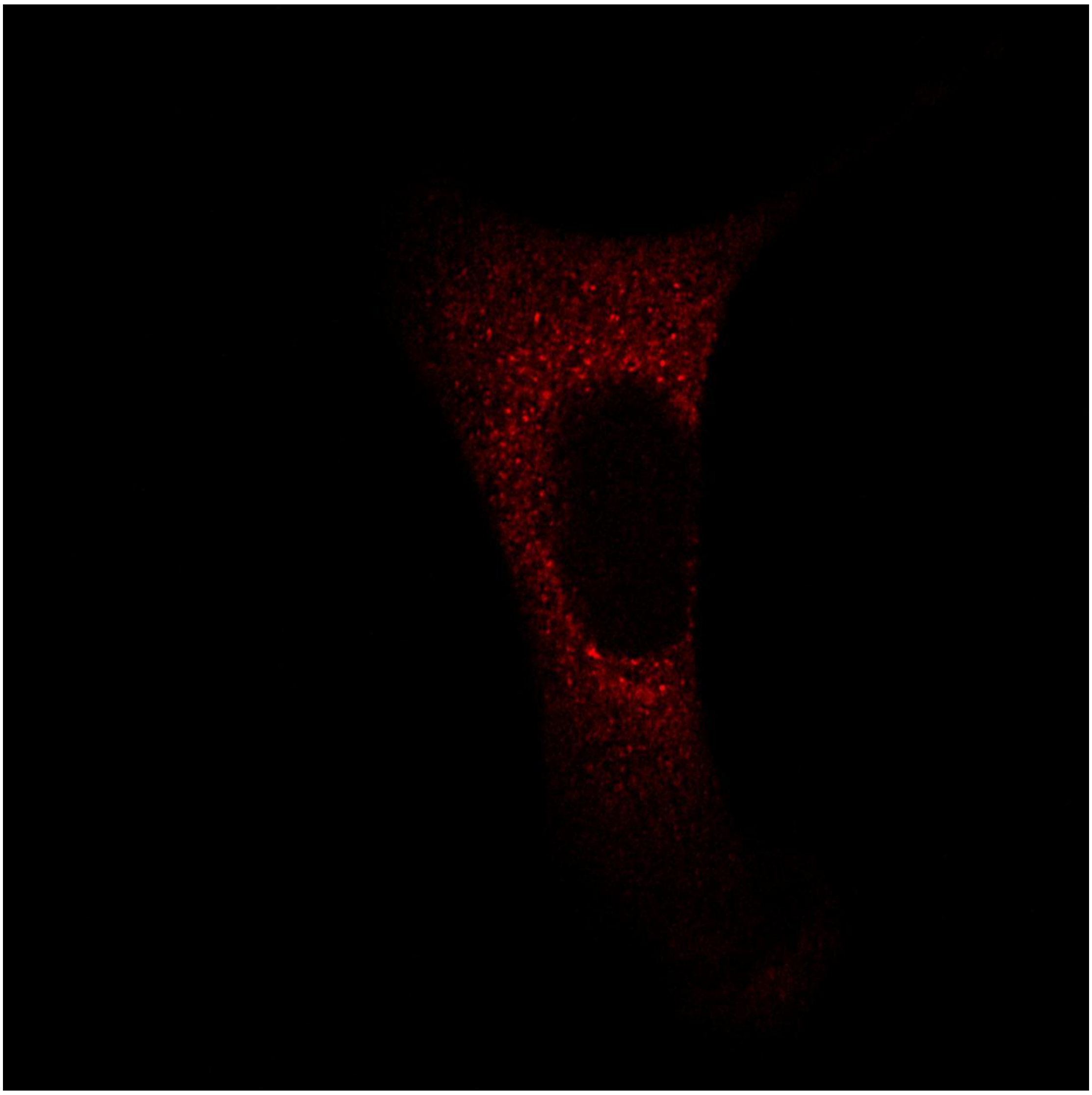

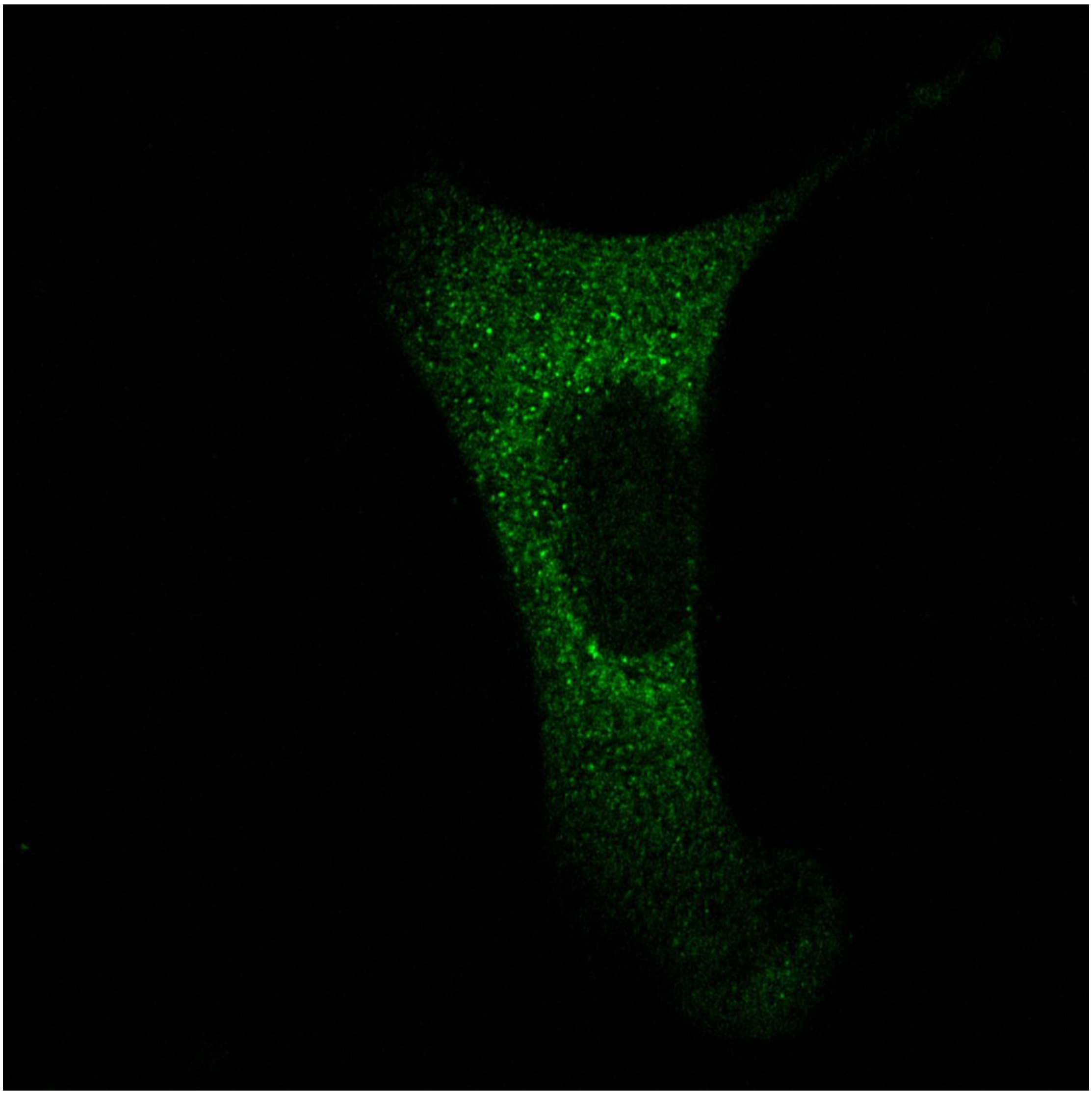

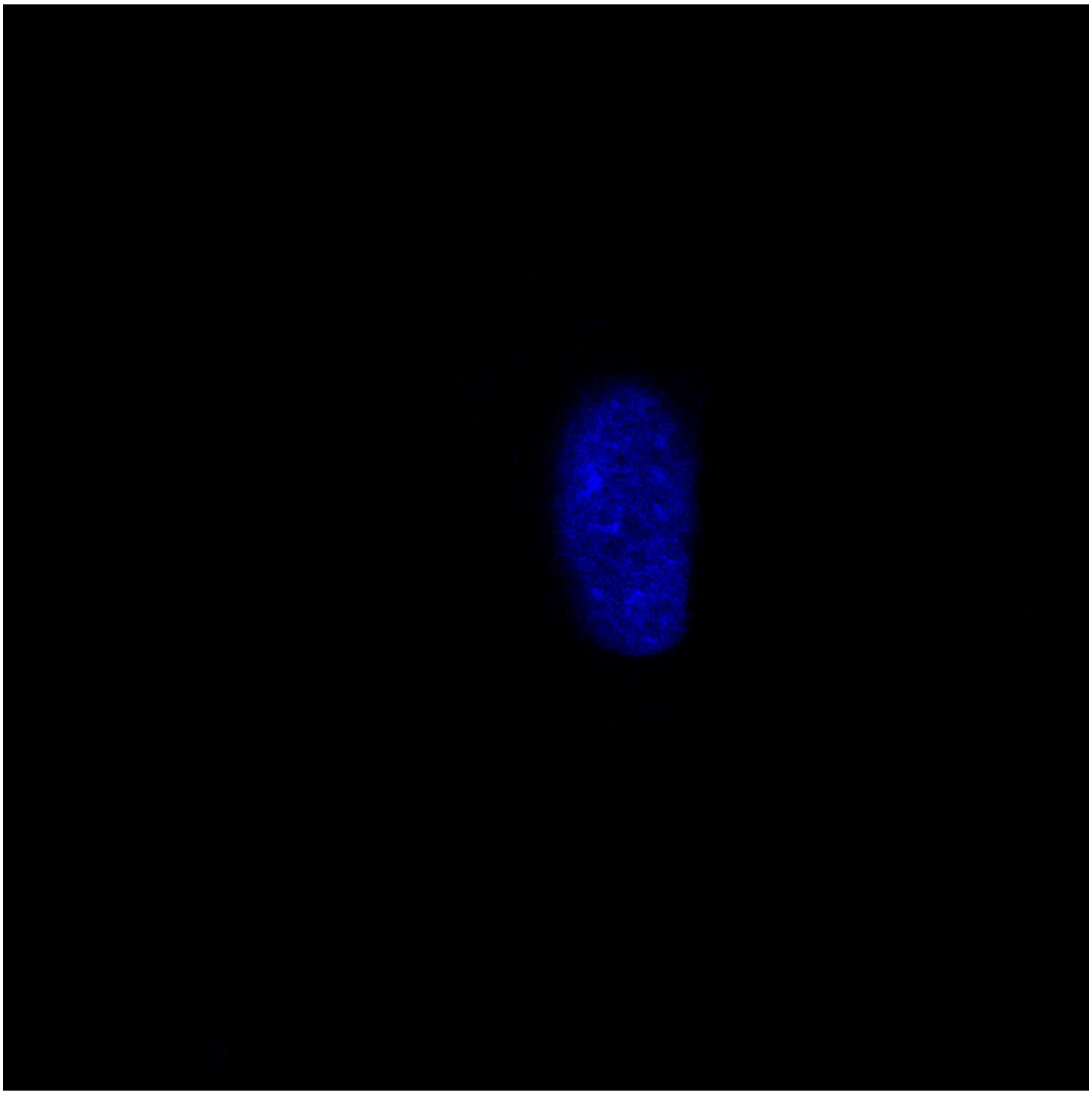

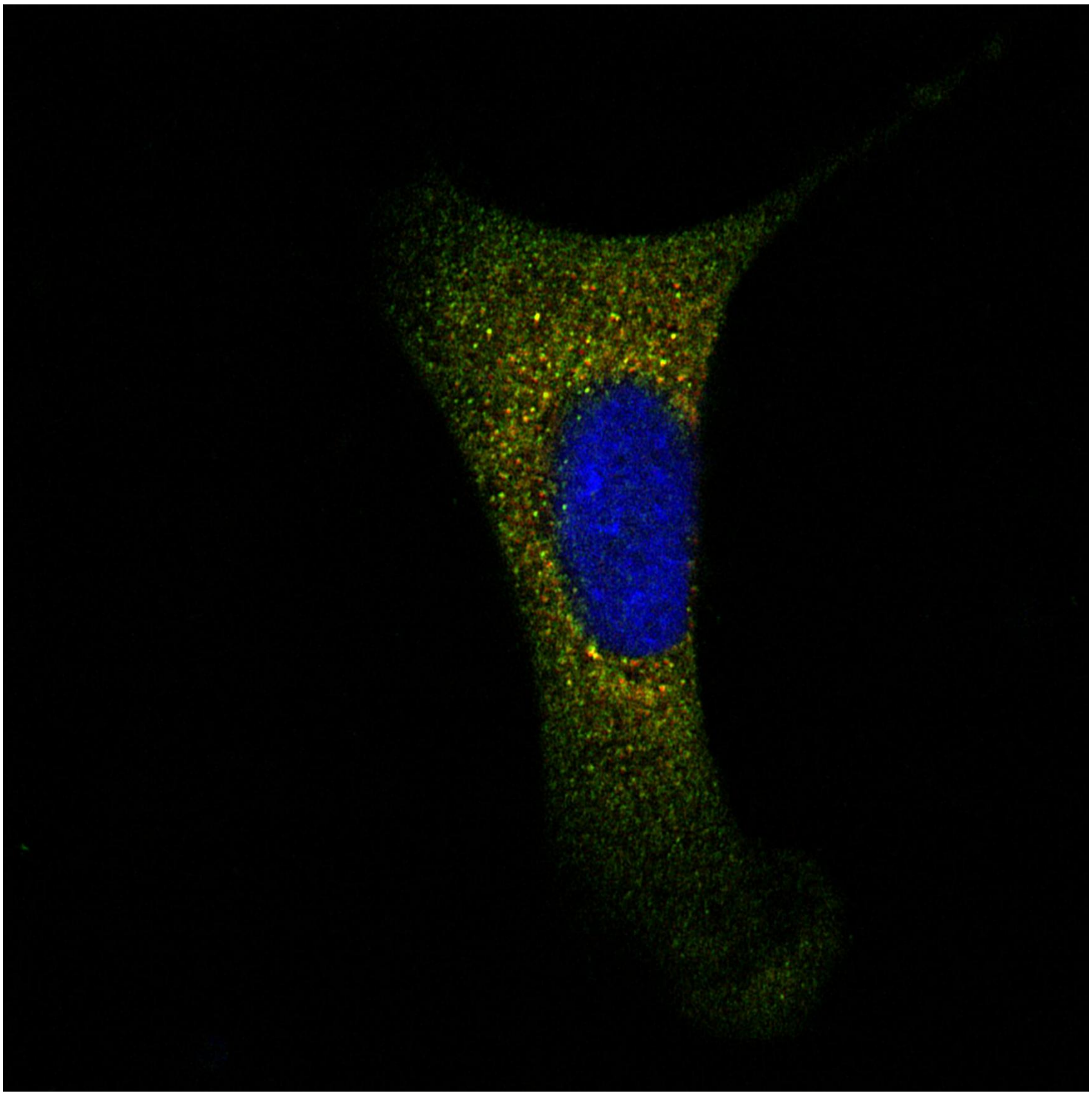

Supplement: S3 File — (PDF) [file pone.0329488.s003.pdf]
